# Rhodanine–Piperazine Hybrids as Potential VEGFR, EGFR, and HER2 Targeting Anti-Breast Cancer Agents

**DOI:** 10.3390/ijms252212401

**Published:** 2024-11-19

**Authors:** Jacek Szczepański, Dmytro Khylyuk, Agnieszka Korga-Plewko, Mariola Michalczuk, Sławomir Mańdziuk, Magdalena Iwan, Nazar Trotsko

**Affiliations:** 1 Chair and Department of Organic Chemistry, Medical University of Lublin, 4A Chodzki Street, 20-093 Lublin, Poland; jaacek.szczepanski.93@gmail.com (J.S.); dmytro.khylyuk@umlub.pl (D.K.); 2 Independent Medical Biology Unit, Medical University of Lublin, 8B Jaczewski Street, 20-090 Lublin, Poland; agnieszka.korga-plewko@umlub.pl (A.K.-P.); mariola.michalczuk@umlub.pl (M.M.); 3 Department of Clinical Oncology and Chemotherapy, Medical University of Lublin, 8 Jaczewski Street, 20-090 Lublin, Poland; slawomir.mandziuk@umlub.pl; 4 Department of Toxicology, Medical University of Lublin, 8B Jaczewski Street, 20-090 Lublin, Poland; magdalena.iwan@umlub.pl

**Keywords:** rhodanine–piperazine hybrids, anti-breast cancer activity, molecular docking, VEGFR, EGFR, HER2

## Abstract

Breast cancer is one of the most common malignancies affecting women worldwide, with a significant need for novel therapeutic agents to target specific molecular pathways involved in tumor progression. In this study, a series of rhodanine–piperazine hybrids were designed, synthesized, and evaluated for their anticancer activity, targeting key tyrosine kinases such as VEGFR, EGFR, and HER2. Biological screening against breast cancer cell lines (MCF-7, MDA-MB-231, T47D, and MDA-MB-468) revealed 3 of the 13 tested compounds as the most potent, with 5-({4-[bis(4-fluorophenyl)methyl]piperazin-1-yl}methylidene)-2-thioxo-1,3-thiazolidin-4-one (**12**) showing the strongest activity, particularly against the MCF-7 and MDA-MB-468 cell lines. Molecular docking studies indicated favorable binding interactions of compound **12** and its 3-phenyl-2-thioxo-1,3-thiazolidin-4-one analogue (**15**) with HER2, VEGFR, and EGFR, and molecular dynamics simulations further confirmed their stable binding to HER2. These findings highlight the potential of rhodanine–piperazine hybrids as promising leads for developing new anticancer agents targeting breast cancer, particularly HER2-positive subtypes. Further structural optimization could enhance their efficacy and therapeutic profile.

## 1. Introduction

Breast cancer is a serious disease in which abnormal breast cells grow out of control and form tumors. If left unchecked, the tumors can spread throughout the body and become fatal. The earliest form (in situ) is not deadly, but cancer cells can spread into nearby breast tissue (invasion). This creates tumors that cause lumps or cell thickening. Invasive cancers can cause metastasis by spreading to nearby lymph nodes or other organs. Metastasis can be fatal.

Treatment depends on the individual, and it is heavily determined by the type of cancer and its spread. Treatment usually combines surgery, radiation therapy, and chemotherapy. As of the end of 2020, there were 7.8 million women alive who were diagnosed with breast cancer in the past 5 years, making it the world’s most prevalent cancer. In 2020 alone, there were 2.3 million women diagnosed with breast cancer, which caused 685,000 deaths globally [1]. A similar trend continued in 2022 (2.3 million women diagnosed/670,000 deaths) [2].

Breast cancer occurs worldwide in women at any age after puberty but with increasing rates in later stages of life. Breast cancer mortality changed only by a small amount from the 1930s through to the 1970s, when surgery alone was the main mode of treatment (radical mastectomy). Improvements in survival began in the 1990s, when the majority of countries established breast cancer early detection programs that were coupled with comprehensive treatment programs, including effective pharmacological treatment. To date, scientists worldwide have been actively searching for new, effective, and selective agents to target breast cancer cells, as successful breast cancer treatment is one of the three main pillars in reducing global mortality from this serious disease [3].

To combat various cancer-based diseases, considerable efforts have been directed toward developing small molecule inhibitors that specifically target and inhibit various tyrosine kinases (TK) or their signaling pathways. The success of first-generation EGFR inhibitors, such as gefitinib and erlotinib, in the treatment of EGFR-mutated non-small cell lung cancer has underscored the therapeutic potential of EGFR-targeted agents [4].

However, the emergence of resistance mechanisms, such as the T790M mutation, has necessitated the development of second- and third-generation EGFR inhibitors, including osimertinib and rociletinib [5,6]. HER2 is a transmembrane receptor protein with an intracellular tyrosine kinase domain. Unlike other erythroblastic oncogene B (ERBB) family members, HER2 does not directly bind to known ligands. Instead, HER2-mediated signaling is activated through heterodimerization with ligand-activated EGFR or ERBB3, or through homodimerization when HER2 is highly expressed, which is often observed in breast cancer patients [7,8,9].

**Figure 1:**
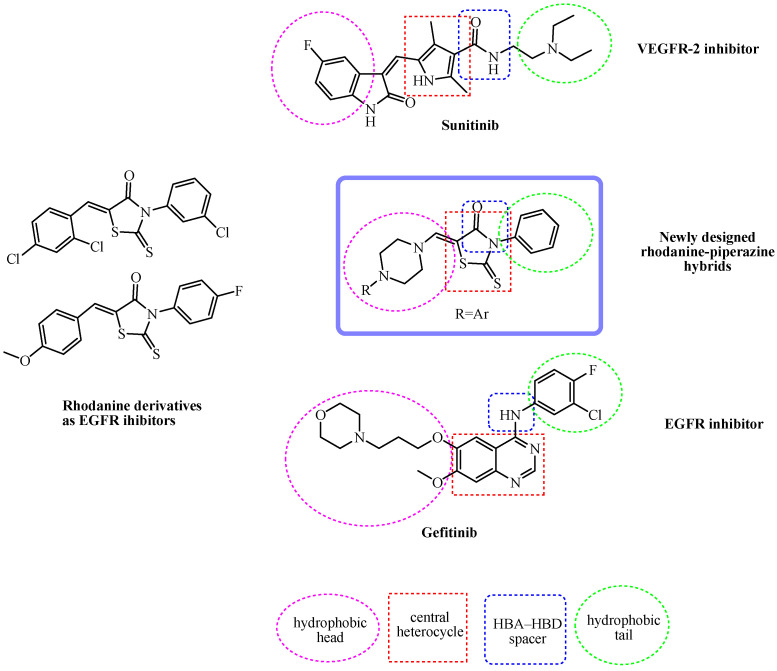
Design of new rhodanine–piperazine hybrids. Abbreviations: HBA—hydrogen bond acc−eptor; HBD—hydrogen bond donor; and Ar—aryl.

Rhodanine derivatives, a class of heterocyclic compounds, have demonstrated a wide range of bioactivities across various therapeutic areas, including anticancer properties. The structural diversity inherent to the rhodanine scaffold has piqued the interest of medicinal chemists, making them attractive candidates for the development of multitargeted agents [10,11]. The strategic design of compounds capable of inhibiting EGFR, VEGFR, and HER2 offers the potential to simultaneously disrupt key signaling pathways involved in both tumorigenesis and angiogenesis. Rhodanine derivatives are intriguing heterocyclic compounds that have garnered attention due to their structural diversity and the pharmacophoric potential inherent in their ring system. Over the past few decades, they have been investigated for their bioactivity across various therapeutic areas, including for antiviral [11,12], anti-inflammatory [13,14], and antitumor properties [15,16,17]. Recent research has identified rhodanine derivatives, which express their anticancer activity through EGFR inhibition [18,19]. In addition, the rhodanine scaffold is suitable for strait drug design of the new VEGFR/ERGF inhibitors according to the pharmacophore model, which consists of four parts: (1) hydrophobic tail, (2) central heteroaromatic ring, (3) spacer, and (4) hydrophobic head [20]. Bearing in mind the above mentioned base structure features of EGFR inhibitors, we utilized the rhodanine core as a central heteroaromatic ring for designing the new anticancer agents with a possible affinity to the VEGFR/EGFR tyrosine kinase and HER2 receptors (Figure 1).

**Figure 2:**
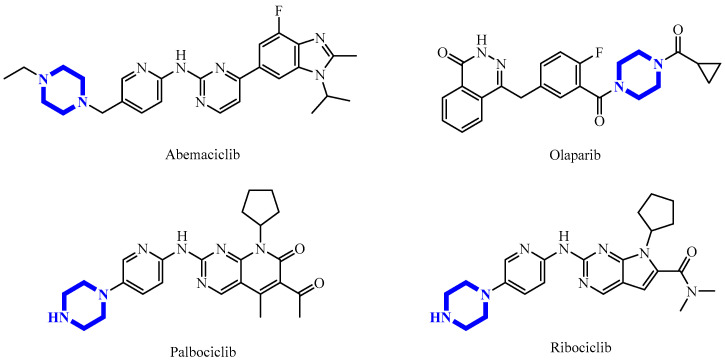
Examples of kinase inhibitors containing a piperazine ring and used as anti-breast cancer drugs.

In addition, drugs officially approved by the US Food and Drug Administration agency containing the piperazine system (marked blue in Figure 2) and being used in the treatment of certain kinds of breast cancer are as follows: abemaciclib, olaparib, palbociclib, and ribociclib. All of these drugs are kinase inhibitors. For example, palbociclib was developed for the treatment of HR-positive and HER2-negative breast cancer [21]. It was the first cyclin-dependent kinases CDK4 and CDK6 inhibitor registered as a cancer therapy [22].

## 2. Results and Discussion

### 2.1. Chemistry

Based on the literature data regarding anticancer activity of rhodanine derivatives [17] and the pharmacophore model for VEGFR/EGFR inhibitors described in the scientific literature [18], new rhodanine–piperazine hybrids were designed as potential VEGFR/EGFR inhibitors (Figure 1).

Rhodanine (**1**) and 3-phenylrhodanine (**2**) were used as starting compounds in the present work. Compound **1** was obtained from potassium chloroacetate and ammonium thiocyanate according to the method described earlier in [23]. Compound **2** was obtained by the reaction of aniline with carbon disulfide in a basic medium and subsequent alkylation by potassium chloroacetate. The resulting intermediate was then cyclized by aqueous hydrochloric acid. This reaction was carried out according to the previously published method in [24]. Rhodanine and 3-phenylrhodanine (**1**, **2**) were then reacted with triethyl orthoformate in acetic anhydride according to the method described earlier in [25].

**Scheme 1:**
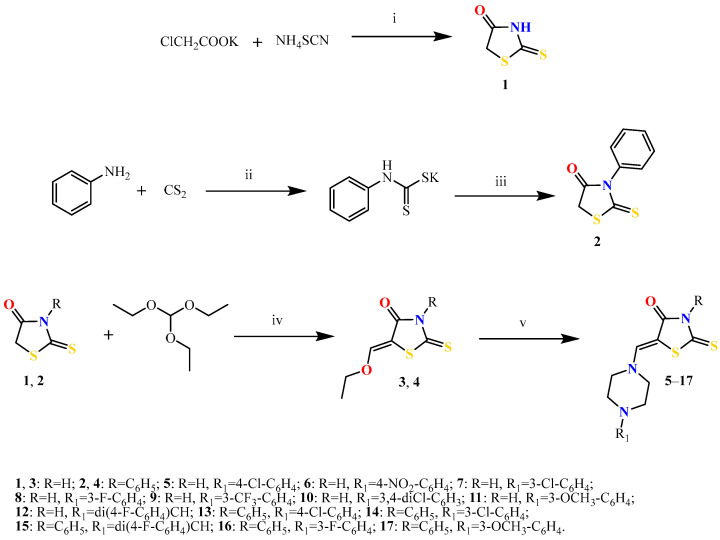
Synthesis of rhodanine–piperazine hybrids (**5**–**17**). Reagents and conditions: (i) HCl at r.t., then 90 °C, 5 min; (ii) KOH, 0–5 °C; (iii) ClCH_2_COOK, 0–5 °C; HCl, 90 °C, 5 min; (iv) Ac_2_O, reflux, 3 h; (v) anhydrous ethanol, reflux, 30 min.

As a result of the reaction, 5-ethoxymethylidene-rhodanine (**3**) and 5-ethoxymethylidene-3-phenylrhodanine (**4**) were obtained. Compounds **3** and **4** were used for obtaining rhodanine–piperazine hybrids (**5**–**17**) by nucleophilic substitution of ethoxide with *N*-arylpiperazines. Scheme 1 illustrates the synthesis process.

The structures of the title compounds (**5**–**17**) were confirmed by spectral characteristics (IR, ^1^H NMR, and ^13^C NMR).

The presence of an aromatic system as well as aliphatic fragments for rhodanine–piperazine hybrids was confirmed based on the absorption bands observed for CH_ar_. and CH_al_. bonds in the ranges of 3009–3091 cm^−1^ and 2727–3003 cm^−1^, respectively. It is worth noting that the absorption bands originating from C=O and C=S groups of derivatives **5**–**17** were observed in the characteristic ranges for these groups: 1660–1683 cm^−1^ and 1210–1235 cm^−1^, respectively.

The ^1^H NMR spectra of the rhodanine–piperazine hybrids (**5**–**17**) confirm the presence of the piperazine system, which was demonstrated by signals originating from the protons of the four CH_2_ groups in the range of 3.16–3.72 ppm. These signals occurred in the form of multiplets, singlets, or triplets. The exceptions are derivatives **12** and **15**, which show signals of the protons of the four CH_2_ groups in a slightly higher magnetic field in the range of 2.36–3.63 ppm. It is also worth noting that the proton signals originating from the aromatic groups of the rhodanine–piperazine hybrids containing para substitution (**5**, **6**, and **13**) give characteristic two doublets in the range of 6.97–8.08 ppm with a coupling constant of J = 9.0–9.6 Hz.

The characteristic signal of the methylidene proton CH= for the rhodanine–piperazine derivatives was observed as a singlet in the range of 7.62–7.66 ppm for the 3-unsubstituted rhodanine derivatives (**5**–**11**) and a singlet in the lower magnetic field of 7.81–7.84 ppm for the 3-phenyl rhodanine derivatives (**13**, **14**, **16**, and **17**). Exceptions are derivatives **12** and **15**, which show the methylidene proton signal at 7.48 and 7.68 ppm, respectively. The 3-unsubstituted rhodanine–piperazine hybrids (**5**–**12**) show the characteristic signal of the -NH-rhodanine proton in the range of 12.91–12.97 ppm in the form of a singlet.

^13^C NMR spectra confirmed the presence of all carbon atoms in the molecules of the newly obtained compounds **5**–**17**.

The signal of carbon originating from the C=S group of the rhodanine–piperazine hybrids was observed in the range of 192–192.6 ppm for the 3-unsubstituted rhodanine derivatives (**5**–**12**) and 190.6–190.7 ppm for the 3-phenyl derivatives (**13**–**17**).

The signal originating from the carbon atom of the C=O carbonyl group of the heterocyclic system (rhodanine) was observed in the range of 167.2–171.0 ppm for compounds **5**–**17**.

The rhodanine–piperazine compounds showed the signal of the carbon atom originating from the CH= group at δ = 90.4–90.8 ppm (**5**–**12**) or 87.7–88.0 ppm (**13**–**17**).

The presence of the piperazine system for the rhodanine–piperazine derivatives was confirmed by the carbon atom signal from the four CH_2_ groups of this system in the chemical shift range of 46.2–50.6 ppm. The exceptions are derivatives **12** and **15**, which show this signal at 51.4 and 51.5 ppm, respectively. It is worth noting that the derivatives containing a fluorine atom (**8**, **9**, **12**, **15**, and **16**) give carbon atom signals in the form of doublets in the range of 102.7–129.3 ppm and 111.9–152.4 ppm with coupling constants of 20.3–25.0 Hz and 8.0–13.8 Hz, respectively. The coupling constants for the ^13^C and ^19^F nuclei in these derivatives correspond to the literature data for this type of derivatives [26].

Detailed information from IR, ^1^H NMR, and ^13^C NMR spectra is presented in the experimental part and Supplementary Materials (Figures S1–S39).

### 2.2. In Silico ADMET Studies

The ADME parameters were calculated using the SwissADME resource [27]. All tested structures meet the assumptions of Lipinski’s rule of five [28], except for compound **15** (Table 1), which is a 4,4-difluorobenzhydrylpiperazine derivative of 3-phenylrhodanine with the calculated highest value of LogP(MLOGP) = 4.17, where according to the rule this value should not exceed 4.15. This compound also has a molar mass (M) of 507.62 g/mol, which is higher than the maximum allowed in the context of the discussed rule (molar mass ≤ 500 g/mol).

Moreover, most of the tested compounds also meet the rules of Ghose [29], Veber [30], Egan [31], and Muegge [32]. The exception is compound **15**, which showed 2 violations of the Ghose rule M > 480 (M for **15** = 507.62) and molar refractivity (MR) > 130 (MR for **15** = 150.62). In addition, compound **15** showed 1 violation with Muegge’s rule XLOGP3 > 5 (XLOGP3 = 6.02 for **15**). Compounds **13** and **14** also do not meet Muegge’s rule XLOGP3 > 5 (XLOGP3 = 5.04 for **13** and **14**). Furthermore, compound **6** showed TPSA = 138.79 A^2^ which does not meet Egan rule (TPSA > 131.6 A^2^).

All rhodanine–piperazine derivatives should be absorbed from the gastrointestinal tract to a high degree. Compounds **5**–**17** (Table 2) are unlikely to permeate the blood–brain barrier and are also not substrates of P-glycoprotein. Based on the predicted results presented above, the degree of solubility in water for most of the structures tested was determined to be moderately soluble, except compound **15**, which is the largest of the entire group and probably less soluble because of that.

**Figure 3:**
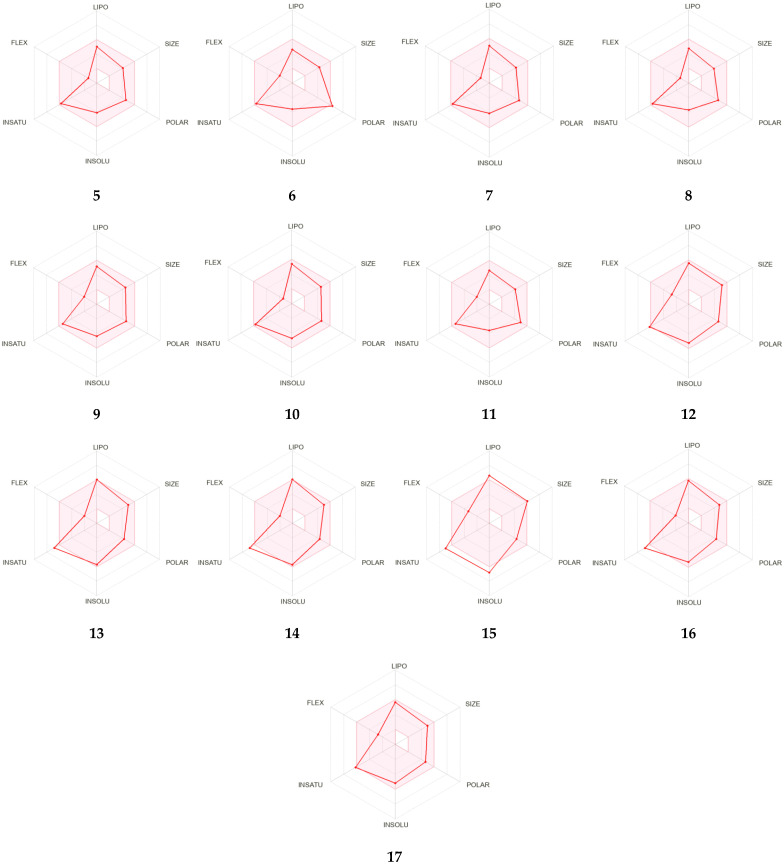
Bioavailability radar for rhodanine–piperazine hybrids (**5**–**17**). The pink area represents the optimal range for each of the properties (lipophilicity: XLOGP3 between −0.7 and +5.0, size: M between 150 and 500 g/mol, polarity: TPSA between 20 and 130 Å^2^, solubility: log S not higher than six, saturation: fraction of carbons in the sp^3^ hybridization not less than 0.25, and flexibility: no more than nine rotatable bonds).

In contrast, the rhodanine–piperazine derivatives **5**–**17** (Figure 3) generally meet all parameters on the bioavailability radar [27], thus fitting into the appropriate physicochemical space (pink field) defined for the expected, correct, oral bioavailability. The exception is structure **15**, which probably does not fit into this space due to its highest molar mass. Data obtained using the SwissADME server suggest that this group of compounds has good potential for being identified as drug candidates.

The ProTox 3.0 server, a virtual laboratory for predicting the toxicity of small molecules [33], was used to predict selected parameters: potential acute toxicity per os, organ toxicity (hepatotoxicity, neurotoxicity, nephrotoxicity, and cardiotoxicity), carcinogenicity, and mutagenicity for compounds **5**–**17**. Toxic doses were given as LD_50_ values in mg/kg body weight, where LD_50_ is the median lethal dose, meaning the dose at which 50% of the tested individuals die after exposure to the compound. Toxicity classes in the ProTox 3.0 server are defined in accordance with the Globally Harmonized System for Classification and Labeling of Chemicals (GHS). LD_50_ values are given in [mg/kg] and classified as follows:

- Class I: fatal if swallowed (LD_50_ ≤ 5);
- Class II: fatal if swallowed (5 < LD_50_ ≤ 50);
- Class III: toxic if swallowed (50 < LD_50_ ≤ 300);
- Class IV: harmful if swallowed (300 < LD_50_ ≤ 2000);
- Class V: may be harmful if swallowed (2000 < LD_50_ ≤ 5000);
- Class VI: non-toxic (LD_50_ > 5000).

The entire series of piperazine derivatives (**5**–**17**) showed expected oral toxicity above 1000 mg/kg (range of values from 1024 to 1233 mg/kg); in addition, these compounds are unlikely to be toxic to the liver, kidneys, and heart (Table 3). The lack of neurotoxic effect is predicted only for structure **6**, which is a 5-substituted rhodanine–piperazine hybrid containing a nitro group. In turn, only this derivative, as the only one from this group, can cause carcinogenicity and mutagenicity, while all other compounds should not show these effects.

### 2.3. Anti-Breast Cancer Activity

In the first stage of evaluation of cytotoxic activity, the cytotoxicity of the obtained derivatives was assessed against normal human fibroblast (BJ) cell lines. The tested compounds showed a percentage of BJ cell growth inhibition in the range of 87–99% at a 200 µM concentration. The half maximal inhibitory concentration for all tested compounds (**5**–**17**) was over 200 µM (IC_50_ > 200 µM).

Next, the cytotoxic activity of all obtained compounds was tested against selected breast cancer cell lines. The research panel consisted of four cancer lines: MDA-MB-468, MDA-MB-231, MCF-7, and T47D. The values of IC_50_ and toxicity curves of the tested compounds based on MTT test results are presented in Table 4 and Figures S40–S52 in the Supplementary Materials.

The MDA-MB-468 cell line, characterized by high EGFR expression and the absence of HER2, showed the highest sensitivity to the tested compounds [34]. Compounds **5**, **6**, **9**, **10**, **12**, **15**, and **17** exhibited low IC_50_ values ranging from 37 to 168 µM, suggesting that their cytotoxic effects are likely associated with EGFR inhibition (Table 4). Gefitinib, a known EGFR inhibitor (IC_50_ = 10 µM), also demonstrated significant activity in this line, supporting the role of EGFR in mediating the effects of these compounds. In the MDA-MB-231 cell line, which expresses moderate levels of EGFR and lacks HER2, compounds such as **6**, **10**, **15**, and **17** showed higher IC_50_ values (ranging from 76 to 118 µM). This suggests that while EGFR may still play a role in the activity of these compounds, the moderate expression of EGFR in this cell line results in a reduced sensitivity compared to MDA-MB-468. Additionally, imatinib (IC_50_ = 5.50 µM), an inhibitor of ABL and PDGFR kinases, suggests that alternative kinase pathways may also contribute to the cytotoxicity in this line. The MCF-7 cell line, characterized by low EGFR expression and moderate HER2 expression, showed lower sensitivity to most compounds. Some compounds (e.g., **10**, **12**, and **15**) had lower IC_50_ values (31 µM, 36 µM, and 45 µM, respectively). These findings suggest that HER2 expression may contribute to the observed activity of these compounds. Imatinib (IC_50_ = 6.33 µM) also highlights the potential involvement of other kinase pathways in the MCF-7 cell line. The T-47D cell line, with low EGFR expression and moderate HER2 expression, exhibited the highest IC_50_ values for most compounds, indicating lower sensitivity. However, compound **10** (IC_50_ = 91 µM) showed moderate effectiveness, possibly due to interactions with HER2. This suggests that HER2 may play a more significant role in mediating the effects of the compounds in this cell line.

In conclusion, the cytotoxicity of the tested compounds varies depending on the EGFR and HER2 expression profiles of the cell lines (Human Protein Atlas, www.proteinatlas.org). The compounds demonstrated greater activity in MDA-MB-468 cells, which express high levels of EGFR, suggesting that EGFR inhibition is a key mechanism of action. In cell lines like MCF-7 and T-47D, the effectiveness of certain compounds may be attributed to moderate HER2 expression. The compounds may also interact with other cellular mechanisms or targets beyond EGFR and HER2.

**Table 4:** IC_50_ values for rhodanine–piperazine hybrids (**5**–**17**) against MDA-MB-468, MDA-MB-231, MCF-7, and T47D cell lines.

Compound	Values of IC_50_ (µM)			
	Cell Lines			
	MDA-MB-468	MDA-MB-231	MCF-7	T47D
**5**	168	>200	>200	>200
**6**	54	92	>200	186
**7**	95	157	>200	>200
**8**	>200	155	>200	>200
**9**	63	>200	73	>200
**10**	55	105	31	91
**11**	92	160	>200	>200
**12**	37	147	36	>200
**13**	179	145	67	>200
**14**	>200	>200	>200	>200
**15**	47	76	45	189
**16**	>200	179	>200	>200
**17**	58	118	169	>200
Gefitinib	10.00 ^1^	-	32.2 ^3^	-
Imatinib	-	5.50 ^2^	6.33 ^4^	5.14 ^4^

^1^ Data derived from reference [35], ^2^ data derived from reference [36], ^3^ data derived from reference [37], and ^4^ data derived from reference [38].

**Figure 4:**
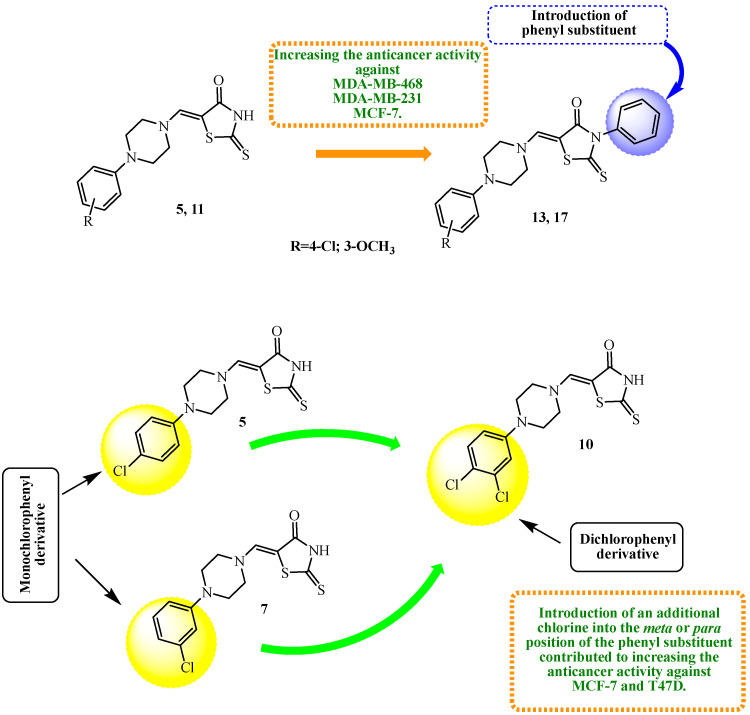
The structure–activity relationship of the tested rhodanine–piperazine hybrids against breast cancer cell lines.

Compound **12**, having a 4,4-difluorobenzhydryl substituent, showed the best inhibitory effect against the MDA-MB-468 cell line (IC_50_ = 37 µM) and was also the most active against MCF-7 (IC_50_ = 36 µM). Its analogue containing a phenyl group in the third position of the rhodanine system, hybrid **15**, caused a very similar cytotoxic effect against the MDA-MB-468 and MCF-7 lines and was almost twice as potent against MDA-MB-231 (IC_50_ = 147 vs. 76 µM). This compound showed no selectivity for the selected breast cancer cell lines, which was similar to **10**. In addition, in the case of the breast cancer lines tested, the advantage of 3,5-disubstituted over 5-substituted rhodanine derivatives can be observed (Figure 4). The proof here is compound **13**, which is definitely more active against MDA-MB-231 (IC_50_ = 145 µM) and MCF-7 (IC_50_ = 67 µM) cell lines, compared to the 5-monosubstituted derivative **5**.

Another example is the 3,5-disubstituted derivative **17**, which showed a much stronger inhibitory effect against MDA-MB-468, MDA-MB-231, and MCF-7 cell lines than its 5-substituted analogue **11** with IC_50_ values of 58 vs. 92 µM, 118 vs. 160 µM, and 169 vs. >200 µM, respectively (Table 4). Similar trends in anticancer activity can be observed in the literature reports [39,40]. Unfortunately, none of the tested compounds were more effective than the reference drugs gefitinib or imatinib. Nevertheless, compounds **10** and **12** showed comparable cytotoxic effects to gefitinib against MCF-7 cells (Table 4). For example, a reported series of thiazol-4-one derivatives containing piperazine moieties showed antiproliferative activity against the MCF-7 cell line with IC_50_ values in the range of 36.2–84.2 µM. The most effective compound from this series was active against MCF-7 with a concentration comparable to the reference gefitinib (36.2 vs. 32.2 µM) [37]. Similar results were observed against MCF-7 for compounds **10** and **12** with IC_50_ values 31 and 36 µM, respectively. In addition, the reported cytotoxic activity of 3,5-disubstituted thiazolidine-2,4-dione derivatives as potential EGFR and VEGFR-2 inhibitors against MCF-7 cells shows IC_50_ values in the range 5.1–79.33 µM [41,42].

To estimate the potential of the obtained compounds for therapeutic use, the selectivity index (SI) was calculated. It is worth noticing that all tested compounds (**5**–**17**) exhibited an SI equal to or more than one (Table 5). SI values higher than one indicate that the tested compound showed higher selectivity for targeting cancer cells over normal cells [43]. The results presented in Table 5 revealed high selectivity for compounds **10**, **12**, and **15**. Compound **10** was most selective for the MCF-7 and T47D cell lines with SI = 6.45 and 2.2, respectively. High selectivity against MCF-7 cells was also showed by compound **12** (SI = 5.56). This hybrid (**12**) was shown to be most effective and safe against MDA-MB-468 cells (SI = 5.41), whereas compound **15** exhibited the highest selectivity index against MDA-MB-231 cells (SI = 2.63).

In summary, the best anticancer potential was shown by compounds **10**, **12**, and **15**, with hybrids **10** and **15** showing activity against the entire breast cancer cells panel at concentrations 31–105 µM and 45–189 µM, respectively.

### 2.4. Molecular Docking

The assessed compounds exhibited binding energies ranging from −6.5 kcal/mol to −9.9 kcal/mol for all examined TKs (Table 6). Compound **15** yielded the highest docking score with HER2, aligning with its strong performance in the biological assays. Furthermore, **15** exhibited the most pronounced affinity towards all three protein structures, with binding results between those of gefitinib and imatinib. However, the co-crystallized ligands from EGFR and VEGFR showed more favorable binding energies compared to all the examined compounds, including **15**.

Compound **12**, identified as the most potent anticancer agent among the set, demonstrated favorable but not superior docking scores for all tested enzymes. Nevertheless, the notable docking scores across all enzymes suggest that **12** and **15** may exert multiple modes of action, even those not connected with the inhibition of TKs. Such diverse affinities may potentially mitigate the development of resistance and expand their utility against various cancer cell lines. In addition, it explains the results of the biological assay where triple negative cancer cell lines (MDA-MB-231) are less sensitive to **12** and **15**, compared to the HER2 positive cell line (MCF-7).

**Figure 5:**
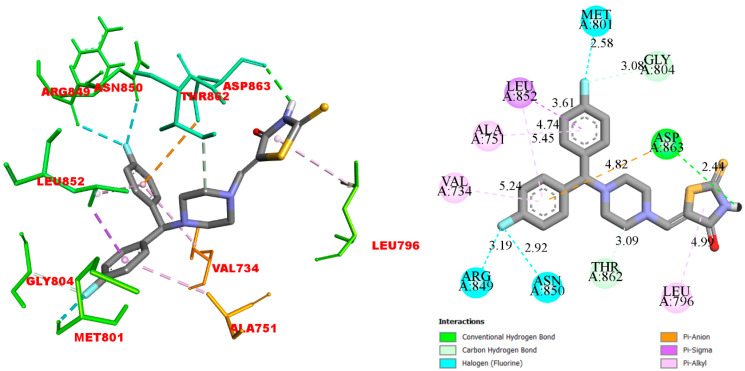
The 3D and 2D schemes of the HER2–compound **12** complex.

Compound **12** forms a hydrogen bond with Asp863 with a length of 2.44 Å. Additionally, the 4-fluorophenyl substituents of compound **12** engage in halogen non-covalent interactions with Met801, Arg849, and Asn850. It has to be mentioned that there is an interaction with Met801, which is significant for inhibition activity of the HER2 tyrosine kinase [44,45]. The two phenyl rings of **12** interact with Leu852, Ala751, and Val734 through π–σ and π–alkyl interactions. Furthermore, a weak carbon–hydrogen bond with Thr862 stabilizes the ligand’s position within the ATP binding cavity (Figure 5).

**Figure 6:**
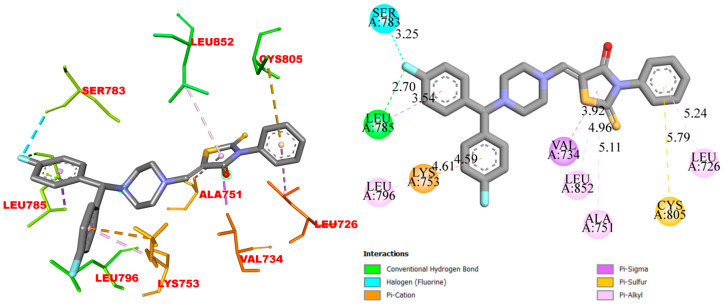
The 3D and 2D schemes of the HER2–compound **15** complex.

With the HER2 receptor, **15** establishes a hydrogen bond with Leu785 (at a distance of 2.70 Å) through the p-fluoro-phenyl core. Furthermore, this same fluorine atom engages in a halogen bond interaction with Ser783. The structural characteristics of the rhodanine ring, along with its phenyl substituent, are well-suited to fit within the channel formed by a cluster of lipophilic amino acids, including Val734, Ala751, Leu852, Cys805, and Leu726. Additionally, another p-fluorophenyl core establishes non-covalent Pi–alkyl and Pi–sulfur interactions with Leu796 and Lys753, respectively. Notably, there is no predicted interaction with Met801, which may significantly influence the inhibitory activity of **15**, despite it having the best binding energy among all tested compounds (Figure 6).

### 2.5. Molecular Dynamics

**Figure 7:**
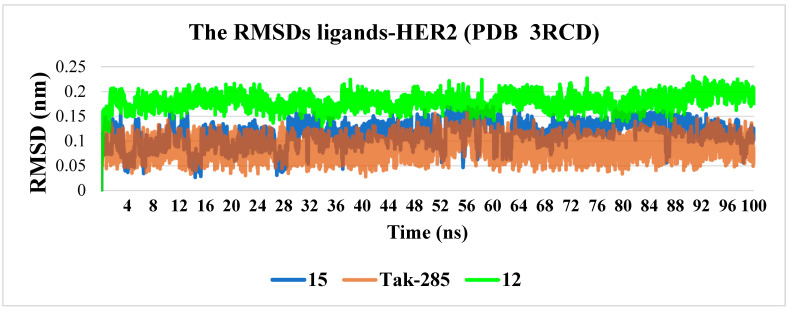
The comparison of the RMSD values for the **12**, **15**, and Tak-285 complexes with HER2 (PDB 3RCD).

In silico simulations demonstrated comparable stability levels for the HER2–Tak-285, HER2–**12**, and HER2–**15** complexes during the 100 ns simulations. Root mean square deviation (RMSD) analysis provides insight into the ligands’ fluctuations over time, enabling the measurement of local fluctuations, such as identifying residues with the highest mobility during the MD simulation (Figure 7). The RMSD values ranged from 0.10 to 0.22 nm, indicating no significant fluctuations. However, the HER2–Tak-285 complex exhibited less fluctuation within the ATP binding site compared to complexes with **12** and **15**. This observation can be attributed to the smaller size of **12** and **15** compared to Tak-285 and the larger amount of hydrogen bonds.

**Figure 8:**
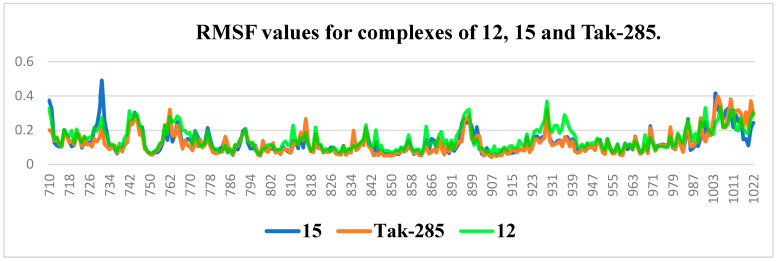
The calculated average RMSF for HER2 complexes with **12**, **15**, and Tak-285.

The average RMSF plots in Figure 8 reveal minimal fluctuations in specific loop regions of the protein structures for all complexes. The low flexibility observed in the complexes with compounds **12** and **15** indicates comparable stability of ligand binding at the binding site when compared to the reference ligand, Tak-285. However, the RMSF plot for the native ligand also showed a slight preference for Tak-285 over **12** and **15**.

**Figure 9:**
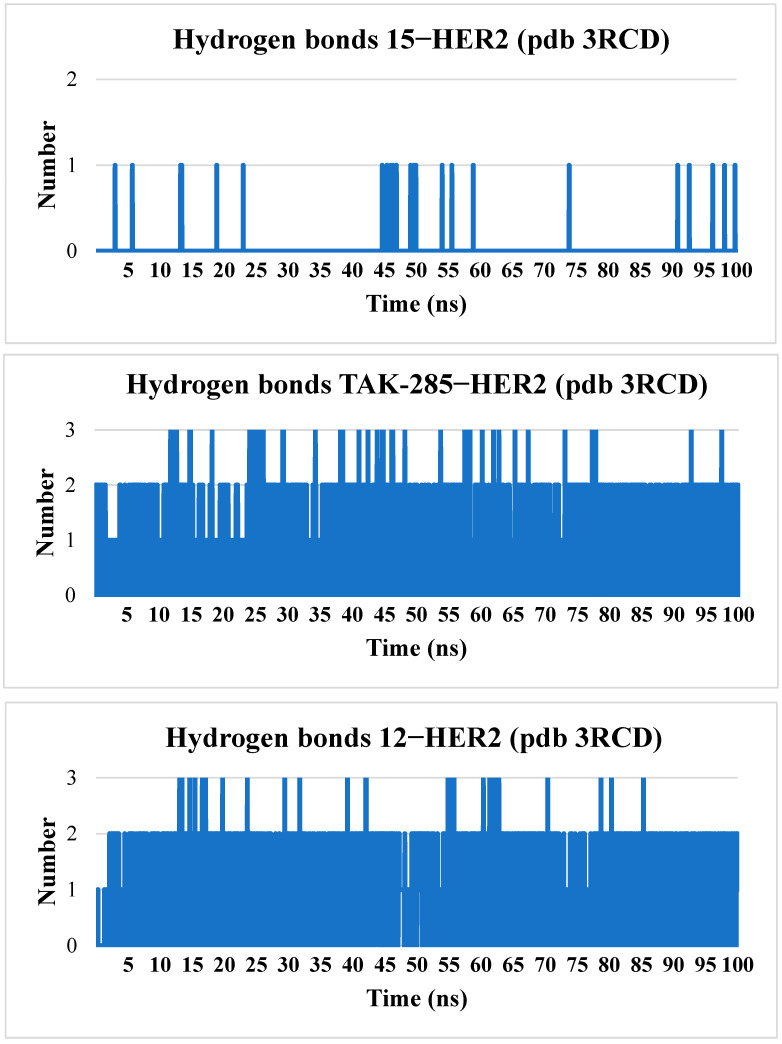
The number of hydrogen bonds during the simulation for complexes of HER2 with **15**, Tak-285, and **12**.

The number of hydrogen bonds in the **15**–HER2 complex was not stable and was often absent, which was expected due to the more lipophilic nature of **15**, compared to the other ligands. In contrast, the complex with **12** demonstrated a stable number of hydrogen bonds, typically ranging from two to three, during the 100 ns simulation. Similarly, the native ligands also showed a stable number of hydrogen bonds, maintaining approximately two to three hydrogen bonds throughout the dynamics procedure (Figure 9).

**Figure 10:**
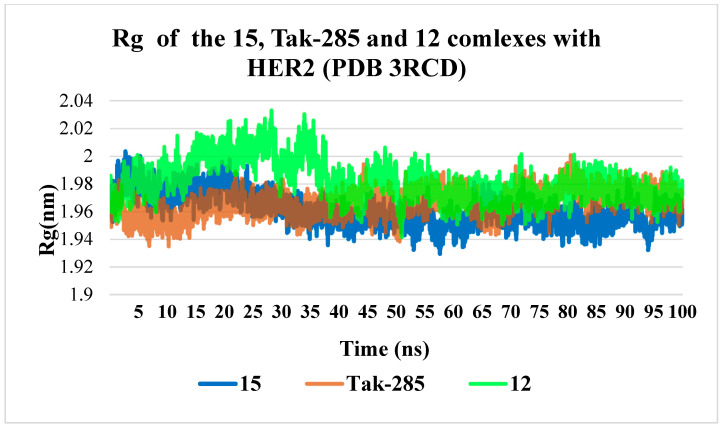
The Rg values for **15**, Tak-285, and **12** complexes with HER2.

The radius of gyration (Rg) is defined as the mass-weighted root mean square distance of a collection of atoms from their common center of mass. A lower Rg indicates a more compact structure. In the context of enzyme inhibition, a more compact inhibitor may fit better into the enzyme’s active site, leading to more effective inhibition. This compactness often correlates with higher stability and potentially better binding affinity to the enzyme. According to the obtained data, the Rg values of all complexes range from 1.93 to 2.03, with a slightly lower Rg for the native ligand compared to the compounds **12** and **15**. Notably, after 100 ns simulations, the Rg values converge, indicating similar compactness and suggesting stability for the compound–HER2 complexes during molecular dynamics simulations (Figure 10).

In silico docking and molecular dynamics assays have demonstrated a correlation between computational predictions and the expression of anticancer activity. However, the ligand with the highest binding energy does not necessarily exhibit the best inhibitory activity. This discrepancy arises because the highest binding energy does not always equate to the most stable complex or optimal interactions with crucial residues essential for inhibition.

Notably, the bis(4-fluorophenyl)methyl piperazine scaffold is integral to the interaction within the ATP binding site of HER2, playing a critical role in the expression of inhibitory activity. The presence or absence of a substituent at the third position of the rhodanine core significantly influences the number of hydrogen bonds and supports interaction with crucial residues for HER2 inhibition.

Overall, compounds **12** and **15** present interesting and potential scaffolds for further structural design of new anticancer agents with possible affinity to HER2.

## 3. Materials and Methods

### 3.1. Chemistry

#### 3.1.1. General

All the chemical reagents and solvents were obtained from commercial companies: Merck Co. (Darmstadt, Germany), Thermo Fisher (Kandel, Germany), the Tokyo Chemical Industry Co. (Tokyo, Japan), and POCH (Gliwice, Poland). They were used without further purification. The purity of the obtained compounds was checked by TLC on plates with sica gel HPTLC Si60F_254_ (Merck Co. (Darmstadt, Germany)). The mobile phase used was a mixture of chloroform and methanol in a volume ratio of 10:1. The melting points were measured by using an Electrothermal Standard 120 VAC apparatus (Cole-Parmer, Wertheim, Niemcy) and are uncorrected. The IR spectra were recorded by a Nicolet 6700 spectrometer (Thermo Scientific, Philadelphia, PA, USA). The ^1^H NMR and ^13^C NMR spectra were recorded by a Bruker Avance DPX 600 instrument (Billerica, MA, USA) using DMSO-*d*_6_ as a solvent and tetramethylsilane as an internal standard.

The starting materials (compounds **1**–**4**) were synthesized by methods described previously in the scientific literature [23,24,25].

#### 3.1.2. General Method for the Synthesis of Rhodanine–Piperazine Hybrids (**5**–**17**)

To 0.01 mol of 5-ethoxymethylidenerhodanine (**3**) or 5-ethoxymethylidene-3-phenylrhodamine (**4**), 0.01 mol of the appropriate piperazine derivatives and 5–10 mL of anhydrous ethanol was added. The reaction mixture was heated under reflux for 30 min. After that, the reaction mixture was cooled. The resulting precipitate was filtered off and dried. The obtained compounds were crystallized from butanol.

5-{[4-(4-Chlorophenyl)piperazin-1-yl]methylidene}-2-thioxo-1,3-thiazolidin-4-one (**5**)
Yield 55%, m.p. = 248 °C–249 °C. FT-IR (ν, cm^−1^): 3078 (CH_ar_); 2958, 2937, 2819 (CH_al._); 1668 (C=O); 1226 (C=S). ^1^H NMR (600 MHz, DMSO-*d*_6_) δ (ppm): 3.25–3.27 m, 3.65 s (8H, 4 × CH_2_); 6.97 d, 7.25 d (4H, 4-Cl-C_6_H_4_, *J* = 9.1 Hz); 7.62 s (1H, CH=); and 12.96 s (1H, NH). ^13^C NMR (150 MHz, DMSO-*d*_6_) δ (ppm): 48.5, 90.5, 117.8, 123.5, 129.2, 143.9, 149.5, 170.0, 192.5.
5-{[4-(4-Nitrophenyl)piperazin-1-yl]methylidene}-2-thioxo-1,3-thiazolidin-4-one (**6**)
Yield 49%, m.p. = 278 °C–279 °C. FT-IR (ν, cm^−1^): 3091 (CH_ar_); 2998, 2903, 2840 (CH_al._); 1678 (C=O); 1228 (C=S). ^1^H NMR (600 MHz, DMSO-*d*_6_) δ (ppm): 3.63–3.65 m, 3.72 s (8H, 4 × CH_2_); 7.03 d, 8.08 d (4H, 4-NO_2_-C_6_H_4_, *J* = 9.6 Hz); 7.66 s (1H, CH=); and 12.97 s (1H, NH). ^13^C NMR (150 MHz, DMSO-*d*_6_) δ (ppm): 46.2, 90.8, 113.2, 126.2, 137.7, 144.1, 154.4, 169.9, 192.6.
5-{[4-(3-Chlorophenyl)piperazin-1-yl]methylidene}-2-thioxo-1,3-thiazolidin-4-one (**7**)
Yield 57%, m.p. = 238 °C–239 °C. FT-IR (ν, cm^−1^): 3061 (CH_ar_); 2996, 2965, 2830 (CH_al._); 1660 (C=O); 1227 (C=S). ^1^H NMR (600 MHz, DMSO-*d*_6_) δ (ppm): 3.31–3.33 m, 3.65 s (8H, 4 × CH_2_); 6.82 ddd (*J* = 0.7, 1.9, 7.9 Hz), 6.92 dd (*J* = 2.4, 7.9 Hz), 6.98 t (*J* = 2.2 Hz), 7.23 t (*J* = 8.0 Hz) (4H, 3-Cl-C_6_H_4_); 7.63 s (1H, CH=); and 12.96 s (1H, NH). ^13^C NMR (150 MHz, DMSO-*d*_6_) δ (ppm): 48.1, 90.5, 114.5, 115.5, 119.1, 131.0, 134.4, 143.9, 151.9, 170.0, 192.5.
5-{[4-(3-Fluorophenyl)piperazin-1-yl]methylidene}-2-thioxo-1,3-thiazolidin-4-one (**8**)
Yield 48%, m.p. = 248 °C–251 °C. FT-IR (ν, cm^−1^): 3069 (CH_ar_); 2979, 2830, 2750 (CH_al._); 1671 (C=O); 1235 (C=S). ^1^H NMR (600 MHz, DMSO-*d*_6_) δ (ppm): 3.31–3.33 m, 3.65 s (8H, 4 × CH_2_); 6.57–6.60 m, 6.76–6.79 m, 7.24 q (*J* = 7.9 Hz) (4H, 3-F-C_6_H_4_); 7.64 s (1H, CH=); and 12.96 s (1H, NH). ^13^C NMR (150 MHz, DMSO-*d*_6_) δ (ppm): 48.1, 90.6, 102.7 d (*J* = 25.0 Hz), 105.8 d (*J* = 21.4 Hz), 111.7, 131.0 d (*J* = 9.7 Hz), 143.9, 152.4 d (*J* = 9.9 Hz), 162.9, 164.5, 170.0, 192.5.
5-{[4-(3-Fluoromethylphenyl)piperazin-1-yl]methylidene}-2-thioxo-1,3-thiazolidin-4-one (**9**)
Yield 53%, m.p. = 251°C–252 °C. FT-IR (ν, cm^−1^): 3042 (CH_ar_); 2917, 2849, 2727 (CH_al._); 1679 (C=O); 1224 (C=S). ^1^H NMR (600 MHz, DMSO-*d*_6_) δ (ppm): 3.36–3.40 m, 3.67 s (8H, 4 × CH_2_); 7.11 d (*J* = 7.6 Hz), 7.21 s, 7.25 dd (*J* = 2.4, 8.4 Hz), 7.44 t (*J* = 8.0 Hz) (4H, 3-CF_3_-C_6_H_4_); 7.64 s (1H, CH=); and 12.96 s (1H, NH). ^13^C NMR (150 MHz, DMSO-*d*_6_) δ (ppm): 48.0, 90.6, 111.9 d (*J* = 13.8 Hz), 115.7 d (*J* = 10.0 Hz), 119.6, 123.9, 125.8, 130.6, 143.9, 150.9, 170.0, 192.5.
5-{[4-(3,4-Dichlorophenyl)piperazin-1-yl]methylidene}-2-thioxo-1,3-thiazolidin-4-one (**10**)
Yield 62%, m.p. = 278 °C–279 °C. FT-IR (ν, cm^−1^): 3063 (CH_ar_); 2974, 2913, 2837 (CH_al._); 1663 (C=O); 1223 (C=S). ^1^H NMR (600 MHz, DMSO-*d*_6_) δ (ppm): 3.33–3.35 m, 3.64 s (8H, 4 × CH_2_); 6.95 dd (*J* = 2.9, 9.0 Hz), 7.17 d (*J* = 2.9 Hz), 7.42 d (*J* = 9.0 Hz) (3H, 3,4-diCl-C_6_H_3_); 7.63 s (1H, CH=); and 12.94 s (1H, NH). ^13^C NMR (150 MHz, DMSO-*d*_6_) δ (ppm): 47.9, 90.6, 116.1, 117.1, 120.6, 131.1, 132.1, 143.9, 150.3, 170.1, 192.5.
5-{[4-(3-Methoxyphenyl)piperazin-1-yl]methylidene}-2-thioxo-1,3-thiazolidin-4-one (**11**)
Yield 56%, m.p. = 198 °C–200 °C. FT-IR (ν, cm^−1^): 3041 (CH_ar_); 2996, 2957, 2838 (CH_al._); 1668 (C=O); 1229 (C=S). ^1^H NMR (600 MHz, DMSO-*d*_6_) δ (ppm): 3.25–3.27 m, 3.65 s (8H, 4 × CH_2_); 3.72 s (3H, OCH_3_); 6.41 dd (*J* = 2.3, 8.1 Hz), 6.49 t (*J* = 2.3 Hz), 6.55 dd (*J* = 2.0, 8.0 Hz), 7,13 t (*J* = 8.2 Hz) (4H, 3-OCH_3_-C_6_H_4_); 7.63 s (1H, CH=); and 12.96 s (1H, NH). ^13^C NMR (150 MHz, DMSO-*d*_6_) δ (ppm): 48.7, 55.4, 90.4, 102.5, 105.4, 108.9, 130.3, 143.9, 152.0, 160.7, 170.1, 192.5.
5-({4-[Bis(4-fluorophenyl)methyl]piperazin-1-yl}methylidene)-2-thioxo-1,3-thiazolidin-4-one (**12**)
Yield 55%, m.p. = 260 °C–263 °C. FT-IR (ν, cm^−1^): 3067 (CH_ar_); 2999, 2958, 2850, 2800 (CH_al._); 1670 (C=O); 1215 (C=S). ^1^H NMR (600 MHz, DMSO-*d*_6_) δ (ppm): 2.36–2.38 m, 3.53 s (8H, 4 × CH_2_); 4.48 s (1H, CH); 7.15 t (*J* = 8.9 Hz), 7.44 dd (*J* = 5.6, 8.7 Hz) (8H, 2 × 4-F-C_6_H_4_); 7.48 s (1H, CH=); and 12.91 s (1H, NH). ^13^C NMR (150 MHz, DMSO-*d*_6_) δ (ppm): 51.4, 72.6, 90.3, 115.9 d (*J* = 21.2 Hz), 129.9 d (*J* = 8.0 Hz), 138.5, 143.7, 160.8, 162.5, 170.0, 192.5.
5-{[4-(4-Chlorophenyl)piperazin-1-yl]methylidene}-3-phenyl-2-thioxo-1,3-thiazolidin-4-one (**13**)
Yield 50%, m.p. = 252 °C–253 °C. FT-IR (ν, cm^−1^): 3048 (CH_ar_); 3003, 2904, 2825 (CH_al._); 1683 (C=O); 1224 (C=S). ^1^H NMR (600 MHz, DMSO-*d*_6_) δ (ppm): 3.30–3.32 m, 3.75 s (8H, 4 × CH_2_); 6.99 d, 7.27 d (4H, 4-Cl-C_6_H_4_, *J* = 9.0 Hz); 7.25 d, 7.47 t, 7.52 t (5H, C_6_H_5_, *J* = 7.2 Hz); and 7.83 s (1H, CH=). ^13^C NMR (150 MHz, DMSO-*d*_6_) δ (ppm): 48.6, 87.9, 117.8, 123.5, 129.2, 129.3, 129.4, 129.6, 136.7, 144.8, 149.5, 167.8, 190.6.
5-{[4-(3-Chlorophenyl)piperazin-1-yl]methylidene}-3-phenyl-2-thioxo-1,3-thiazolidin-4-one (**14**)
Yield 65%, m.p. = 242 °C–243 °C. FT-IR (ν, cm^−1^): 3024 (CH_ar_); 2996, 2924, 2843 (CH_al._); 1674 (C=O); 1227 (C=S). ^1^H NMR (600 MHz, DMSO-*d*_6_) δ (ppm): 3.37 t, 3.74 t (8H, 4 × CH_2_, *J* = 5.2 Hz); 6.84 dd (*J* = 1.9, 7.8 Hz), 6.94 dd (*J* = 2.4, 8.3 Hz), 7.01 t (*J* = 2.2 Hz), 7.45–7.49 m (4H, 3Cl-C_6_H_4_); 7.23–7.27 m, 7.51–7.54 m (5H, C_6_H_5_); and 7.83 s (1H, CH=). ^13^C NMR (150 MHz, DMSO-*d*_6_) δ (ppm): 48.5, 88.0, 114.5, 115.5, 119.2, 129.2, 129.4, 129.6, 131.1, 134.4, 136.7, 144.9, 151.8, 167.8, 190.7.
5-({4-[Bis(4-fluorophenyl)methyl]piperazin-1-yl}methylidene)-3-phenyl-2-thioxo-1,3-thiazolidin-4-one (**15**)
Yield 57%, m.p. = 257 °C–260 °C. FT-IR (ν, cm^−1^): 3041 (CH_ar_); 3000, 2914, 2813 (CH_al._); 1675 (C=O); 1219 (C=S). ^1^H NMR (600 MHz, DMSO-*d*_6_) δ (ppm): 2.41 s, 3.63 s (8H, 4 × CH_2_); 4.51 s (1H, CH); 7.16 t (*J* = 8.8 Hz), 7.21–7.24 m, 7.38–7.60 m (13H, 2 × 4-F-C_6_H_4_ and C_6_H_5_); and 7.68 s (1H, CH=). ^13^C NMR (150 MHz, DMSO-*d*_6_) δ (ppm): 51.5, 72.6, 87.7, 115.9 d (*J* = 21.1 Hz), 129.3 d (*J* = 20.3 Hz), 129.6, 130.0 d (*J* = 8.0 Hz), 136.7, 138.5, 144.7, 160.9, 162.5, 167.8, 190.7.
5-{[4-(3-Fluorophenyl)piperazin-1-yl]methylidene}-3-phenyl-2-thioxo-1,3-thiazolidin-4-one (**16**)
Yield 51%, m.p. = 237 °C–238 °C. FT-IR (ν, cm^−1^): 3009 (CH_ar_); 2996, 2918, 2842 (CH_al._); 1675 (C=O); 1231 (C=S). ^1^H NMR (600 MHz, DMSO-*d*_6_) δ (ppm): 3.37 t, 3.74 t (8H, 4 × CH_2_, *J* = 5.1 Hz); 6.60 td (*J* = 1.9, 7.3, 7.8 Hz), 6.76–6.83 m, 7.19–7.30 m, 7.41–7.45 m (9H, 3-F-C_6_H_4_ and C_6_H_5_); and 7.84 s (1H, CH=). ^13^C NMR (150 MHz, DMSO-*d*_6_) δ (ppm): 48.2, 88.0, 102.7 d (*J* = 25.4 Hz), 105.8 d (*J* = 21.4 Hz), 129.2, 129.4, 129.6, 131.0 d (*J* = 10.3 Hz), 136.7, 144.9, 152.3 d (*J* = 10.6 Hz), 163.0, 164.6, 167.2, 190.6.
5-{[4-(3-Methoxyphenyl)piperazin-1-yl]methylidene}-3-phenyl-2-thioxo-1,3-thiazolidin-4-one (**17**)
Yield 46%, m.p. = 197 °C–199 °C. FT-IR (ν, cm^−1^): 3069 (CH_ar_); 2998, 2955, 2899, 2826 (CH_al._); 1682 (C=O); 1222 (C=S). ^1^H NMR (600 MHz, DMSO-*d*_6_) δ (ppm): 3.30–3.31 m (4H, 2 × CH_2_); 3.73–3.75 m (7H, 2 × CH_2_ and OCH_3_); 6.42 dd (*J* = 2.3, 8.1 Hz), 6.50 t (*J* = 2.3 Hz), 6.56 dd (*J* = 2.3, 8.3 Hz), 7.15 t (*J* = 8.2 Hz) (4H, 3-OCH_3_-C_6_H_4_); 7.23–7.28 m, 7.45–7.55 m (5H, C_6_H_5_); and 7.83 s (1H, CH=). ^13^C NMR (150 MHz, DMSO-*d*_6_) δ (ppm): 48.8, 55.4, 87.9, 102.5, 105.4, 108.9, 129.2, 129.4, 129.6, 130.3, 136.7, 144.8, 152.0, 160.7, 167.8, 190.6.

### 3.2. ADMET Studies

The prediction of ADME parameters was performed using the online SwissADME program [27]. Toxicity prediction was performed using the ProTox 3.0 program [33]. Acute oral toxicity and organ toxicity (liver, heart, kidney, CNS), as well as carcinogenicity and mutagenicity were taken into account.

### 3.3. Cell Lines and Culture Conditions

Cytotoxicity of tested compounds was evaluated against breast cancer cell lines (MDA-MB-468, MDA-MB-231, MCF-7, and T47D) and normal human fibroblast BJ cell line as a control. All tested cell lines were obtained from the American Type Culture Collection (ATCC, Manassas, VA, USA). MDA-MB-468 and MDA-MB-231 were cultured in F12-K medium (Corning, New York City, NY, USA), MCF-7 and BJ in EMEM medium (Corning, New York City, NY, USA), and T47D in RPMI medium (Corning, New York City, NY, USA). All of these culture media were supplemented with 10% heat-inactivated FBS (PAN-Biotech, Aidenbach, Germany) and antibiotics (100 U/mL of penicillin, 100 μg/mL of streptomycin, and 0.25 μg/mL amphotericin B, respectively; Sigma-Aldrich, St. Louis, MO, USA) in a humidified 5% CO_2_ atmosphere at 37 °C. The cells were used exclusively for 10 passages.

### 3.4. Cytotoxic Activity

The cells were seeded in 96-well plates at a concentration of 5 × 10^4^ cells/mL and incubated at 37 °C with 5% CO_2_ air. When 70–80% culture confluence was achieved the tested compounds were added. The cells were incubated for 48 h with the tested compounds in concentrations in the range of 1–200 μM or with DMSO (dimethyl sulfoxide, POCH, Poland) as a vehicle in control cultures (DMSO concentration in the cultures did not exceed 0.5%). The cell viability assessment was measured using the MTT assay (Invitrogen, Waltham, MA, USA) based on the ability of living cells to reduce orange tetrazolium salt, by cellular dehydrogenases, to water-insoluble purple formazan crystals. MTT solution (4 mg/mL) was added to the culture after 48 h of incubation with the tested compounds. Following 4 h of incubation, the medium with MTT salt was removed and the obtained crystals were dissolved in DMSO. The solution absorbency was measured at 570 nm using a PowerWave™ microplate spectrophotometer (Bio-Tek Instruments, Winooski, VT, USA). Each experiment was performed twice with measurement in triplicate. The IC_50_ values were determined using the AAT Bioquest IC_50_ calculator with a four parameter logistic regression model (AAT Bioquest, Inc. Pleasanton, CA, USA (14 April 2024). Quest Graph™ IC_50_ Calculator. AAT Bioquest. https://www.aatbio.com/tools/ic50-calculator, accessed on 12 October 2024).

### 3.5. Molecular Docking

In order to examine the theory about the possible antitumor activity through interaction with the different types of tyrosine kinases docking research was performed. As the target enzymes, we chose EGFR—PDB ID 7ZYQ [46], VEGFR—PDB ID 1YWN [47], and HER2—PDB ID 3RCD [44]. AutoDock Vina 1.2.5 was selected as the docking software solution according to its ability to reproduce the positions of co-crystalized ligands inside the selected enzymes [48]. Structure energy minimizations of the tested ligands were performed by Avogadro with the implementation of the force field MMFF94 (20,000 steps) [49]. AutoDock Tools 1.5.6 was used for preparing protein structures for the simulation. The preparatory protocols entail the elimination of water molecules and the introduction of polar hydrogen atoms. Kollman charges were ascribed to the respective residues and distributed across the amino acid constituents. A cubic grid enclosure, measuring 60 Å in each dimension, was appropriately positioned within all the investigated tyrosine kinases. The validation of the designated parameters and software implementations encompassed a conventional methodology involving re-docking the ligand’s structures as extracted from the spectra [50]. In addition, the structure of gefitinib [51] and imatinib [52], as clinically approved drugs were used in the cross-docking studies. The computed binding energies were employed to estimate the affinities of the synthesized compounds concerning the experimentally determined inhibitors. The outcomes of the simulations were derived from the positions of the ligands that achieved the highest docking scores within the examined enzymes. The data visualization and interpretation were conducted using Discovery Studio Visualizer version 21.1 [53].

### 3.6. Molecular Dynamics

Molecular dynamics (MD) simulations were conducted using GROMACS, as accessed via the SiBioLead server (https://sibiolead.com/) to evaluate the stability of the most promising compound’s complexes with the HER2. For comparative analysis of stability, molecular dynamics (MD) simulation was also conducted with the native ligand Tak-285. The obtained complexes during the simulations were corrected by the Swiss-PDB Viewer [54]. The HER2-ligand complexes were solvated in a triclinic box containing water, adding 0.15 M NaCl to neutralize the system’s charge. The simulation system was parameterized using the OPLS/AA force field. Before the production run, the system was equilibrated using the NVT and NPT ensembles over 300 ps. The MD simulation was performed for 100 ns, employing the leap-frog integrator. Trajectory frames were recorded at 20 ps intervals throughout the 100 ns simulation, yielding 5000 frames. The resultant data were analyzed using the built-in trajectory analysis tools in GROMACS (https://sibiolead.com, accessed on 12 October 2024) and Microsoft Excel.

## 4. Conclusions

In this study, novel rhodanine–piperazine hybrids were designed and synthesized as potential inhibitors of VEGFR, EGFR, and HER2 tyrosine kinases, with the aim of developing new anticancer agents targeting breast cancer. All synthesized compounds were evaluated for their anti-breast cancer activity. Compound **10** demonstrated the highest cytotoxic potential against the MCF-7 and T47D breast cancer cell lines. Compound **12**, with a 4,4-difluorobenzhydryl substituent, showed the strongest activity and selectivity against the MDA-MB-468 and MCF-7 cell lines. Furthermore, the 3-phenyl analogue of compound **12**, derivative **15**, although effective against all tested breast cancer cell lines, was particularly potent against MDA-MB-231. Compounds **12** and **15** exhibited favorable binding energies towards HER2, VEGFR, and EGFR tyrosine kinases. The docking studies revealed strong interactions, especially for compound **15**, which formed stable complexes with HER2, potentially contributing to its inhibitory activity. Molecular dynamics simulations demonstrated stable binding of compounds **12** and **15** to HER2, with similar stability to the reference drug Tak-285. Despite the high binding energy of **15**, it did not always translate to superior biological activity, highlighting that stability and interaction with key residues are critical for effective inhibition. Compounds **12** and **15** present promising scaffolds for the development of future anticancer agents targeting HER2, with potential applications in treating various subtypes of breast cancer. Their multitarget activity, particularly against HER2, suggests a possible advantage in overcoming resistance mechanisms seen against traditional TK inhibitors. Overall, the rhodanine–piperazine hybrids synthesized in this study exhibit potential for further structural optimization and development as anticancer agents, particularly for breast cancer treatment.

## Figures and Tables

**Table 1:** Physicochemical properties for compounds **5**–**17** calculated using SwissADME.

Compound	Lipinski’s Rule of Five				
	HBA	HBD	M [g/mol]	LogP (MLOGP)	Violation
**5**	1	1	339.86	1.49	0
**6**	3	1	350.42	0.00	0
**7**	1	1	339.86	1.49	0
**8**	2	1	323.41	1.36	0
**9**	4	1	373.42	1.86	0
**10**	1	1	374.31	2.01	0
**11**	2	1	335.44	0.66	0
**12**	4	1	431.52	2.89	0
**13**	1	0	415.96	2.94	0
**14**	1	0	415.96	2.94	0
**15**	4	0	507.62	4.17	2
**16**	2	0	399.50	2.83	0
**17**	2	0	411.54	2.11	0

HBA—hydrogen bond acceptor; HBD—hydrogen bond donor; M—molar mass; and LogP—partition coefficient.

**Table 2:** Predicted pharmacokinetic parameters of compounds **5**–**17** were obtained using the SwissADME server.

Compound	Pharmacokinetics and Water Solubility			Water Solubility
	GI Absorption	BBB Permeation	P-gp Substrate	
**5**	High	No	No	Moderately soluble
**6**	High	No	No	Soluble
**7**	High	No	No	Moderately soluble
**8**	High	No	No	Soluble
**9**	High	No	No	Moderately soluble
**10**	High	No	No	Moderately soluble
**11**	High	No	No	Soluble
**12**	High	No	No	Moderately soluble
**13**	High	No	No	Moderately soluble
**14**	High	No	No	Moderately soluble
**15**	High	No	Yes	Poorly soluble
**16**	High	No	No	Moderately soluble
**17**	High	No	No	Moderately soluble

**Table 3:** Predicted toxicity values for compounds **5**–**17**, obtained using the ProTox 3.0 server.

Compound	Acute Oral Toxicity LD_50_, mg/kg	Organ Toxicity				Carcinogenicity Prediction/ Probability	Mutagenicity Prediction/ Probability
		Hepatotoxicity Prediction/ Probability	Neurotoxicity Prediction/ Probability	Nephrotoxicity Prediction/ Probability	Cardiotoxicity Prediction/ Probability		
**5**	1233	i*/0.63	a**/0.70	i/0.70	i/0.83	i/0.56	i/0.64
**6**	1233	i/0.61	i/0.69	i/0.61	i/0.74	a/0.66	a/0.82
**7**	1233	i/0.63	a/0.70	i/0.70	i/0.83	i/0.56	i/0.64
**8**	1233	i/0.63	a/0.68	i/0.71	i/0.82	i/0.57	i/0.65
**9**	1233	i/0.65	a/0.66	i/0.73	i/0.83	i/0.56	i/0.63
**10**	1233	i/0.67	a/0.71	i/0.71	i/0.82	i/0.58	i/0.65
**11**	1233	i/0.65	a/0.65	i/0.62	i/0.71	i/0.54	i/0.65
**12**	1024	i/0.67	a/0.73	i/0.73	i/0.82	i/0.59	i/0.67
**13**	1125	i/0.65	a/0.75	i/0.72	i/0.82	i/0.57	i/0.67
**14**	1125	i/0.65	a/0.75	i/0.72	i/0.82	i/0.57	i/0.67
**15**	1125	i/0.66	a/0.73	i/0.73	i/0.82	i/0.58	i/0.68
**16**	1125	i/0.66	a/0.73	i/0.73	i/0.82	i/0.58	i/0.68
**17**	1125	i/0.68	a/0.70	i/0.64	i/0.67	i/0.54	i/0.64

i*—inactive; the compound does not exhibit a given toxic effect with a certain probability. a**—active; the compound exhibits a given toxic effect with a certain probability.

**Table 5:** Selectivity index (SI) for rhodanine–piperazine hybrids (**5**–**17**).

Compound	Selectivity index (SI)			
	SI_MDA-MB-468_	SI_MDA-MB-231_	SI_MCF-7_	SI_T47D_
**5**	1.19	1	1	1
**6**	3.7	2.17	1	1.08
**7**	2.1	1.27	1	1
**8**	1	1.29	1	1
**9**	3.17	1	2.74	1
**10**	3.63	1.90	6.45	2.2
**11**	2.17	1.25	1	1
**12**	5.41	1.36	5.56	1
**13**	1.12	1.38	2.99	1
**14**	1	1	1	1
**15**	4.26	2.63	4.44	1.06
**16**	1	1.12	1	1
**17**	3.45	1.69	1.18	1

SI—selectivity index, which means the IC_50_(BJ)/IC_50_(cancer cells) ratio; SI_MDA-MB-468_ = IC_50_(BJ)/IC_50_(MDA-MB-468); SI_MDA-MB-231_ = IC_50_(BJ)/IC_50_(MDA-MB-231); SI_MCF-7_ = IC_50_(BJ)/IC_50_(MCF-7); and SI_T47D_ = IC_50_(BJ)/IC_50_(T47D).

**Table 6:** Vina docking score of the assessed compounds.

Compounds	EGFR (PDB 7ZYQ)	VEGFR (PDB 1YWN)	HER2 (PDB 3RCD)
**5**	−6.9	−6.7	−7.1
**6**	−7.1	−6.5	−7.6
**7**	−7.2	−6.8	−8.3
**8**	−6.9	−6.8	−8.2
**9**	−7.5	−7.2	−8.8
**10**	−6.9	−6.9	−7.9
**11**	−7.0	−6.6	−8.0
**12**	−8.2	−8.2	−8.9
**13**	−7.6	−7.0	−8.5
**14**	−8.4	−7.6	−9.0
**15**	−8.4	−9.0	−9.9
**16**	−7.6	−7.4	−9.1
**17**	−8.3	−7.3	−8.9
CHEMBL5270693	−11.3	−8.1	−9.1
Gefitinib	−7.8	−	−8.3
Imatinib	−9.2	−	−10.5
GW768505A	-	−11.7	-
Tak-285			−9.9

## Data Availability

Data are contained within the article.

## Supplementary Materials

The following supporting information can be downloaded at: https://www.mdpi.com/article/10.3390/ijms252212401/s1.
