# Supplementary material for: Rhodanine–Piperazine Hybrids as Potential VEGFR, EGFR, and HER2 Targeting Anti-Breast Cancer Agents
Source: Int J Mol Sci. 2024 Nov 19;25(22):12401. doi: 10.3390/ijms252212401 (PMC11594716; doi:10.3390/ijms252212401)
Supplement: Supplementary file 1 [file ijms-25-12401-s001.zip › ijms-3283929-supplementary.pdf]

Supplemental materials for:

Rhodanine-piperazine hybrids as potential VEGFR, EGFR, and HER2 targeting anti-breast cancer agents.

Jacek Szczepański [1], Dmytro Khylyuk [1], Agnieszka Korga-Plewko [2], Mariola Michalczuk[2], Sławomir Mańdziuk [3], Magdalena Iwan[4] and Nazar Trotsko [1,*]

[1] Chair and Department of Organic Chemistry, Medical University of Lublin, 4A Chodzki Street, 20-093 Lublin, Poland; jaacek.szczepanski.93@gmail.com (J.S.); dmytro.khylyuk@umlub.pl (D.K.); nazar.trotsko@umlub.pl (N.T.)

[2] Independent Medical Biology Unit, Medical University of Lublin, 8B Jaczewski Street, 20-090 Lublin, Poland; agnieszka.korga-plewko@umlub.pl (A.K.-P.), mariola.michalczuk@umlub.pl (M.M.)

[3] Department of Clinical Oncology and Chemotherapy, Medical University of Lublin, 8 Jaczewski Street, 20-090 Lublin, Poland; slawomir.mandziuk@umlub.pl (S.M.)

[4] Department of Toxicology, Medical University of Lublin, 8B Jaczewski Street, 20-090 Lublin, Poland; Magdalena.iwan@umlub.pl (M.I.)

* Correspondence: nazar.trotsko@umlub.pl (N.T.)

Table of content:

1. FT-IR spectra of rhodanine-piperazine hybrids (**5**–**17**)...S2
2. ^{1}H NMR spectra of rhodanine-piperazine hybrids (**5**–**17**)..................................S9
3. ^{13}C NMR spectra of rhodanine-piperazine hybrids (**5**–**17**)................................S22
4. Toxicity curves of tested compounds (**5**–**17**) based on MTT test results….....S35

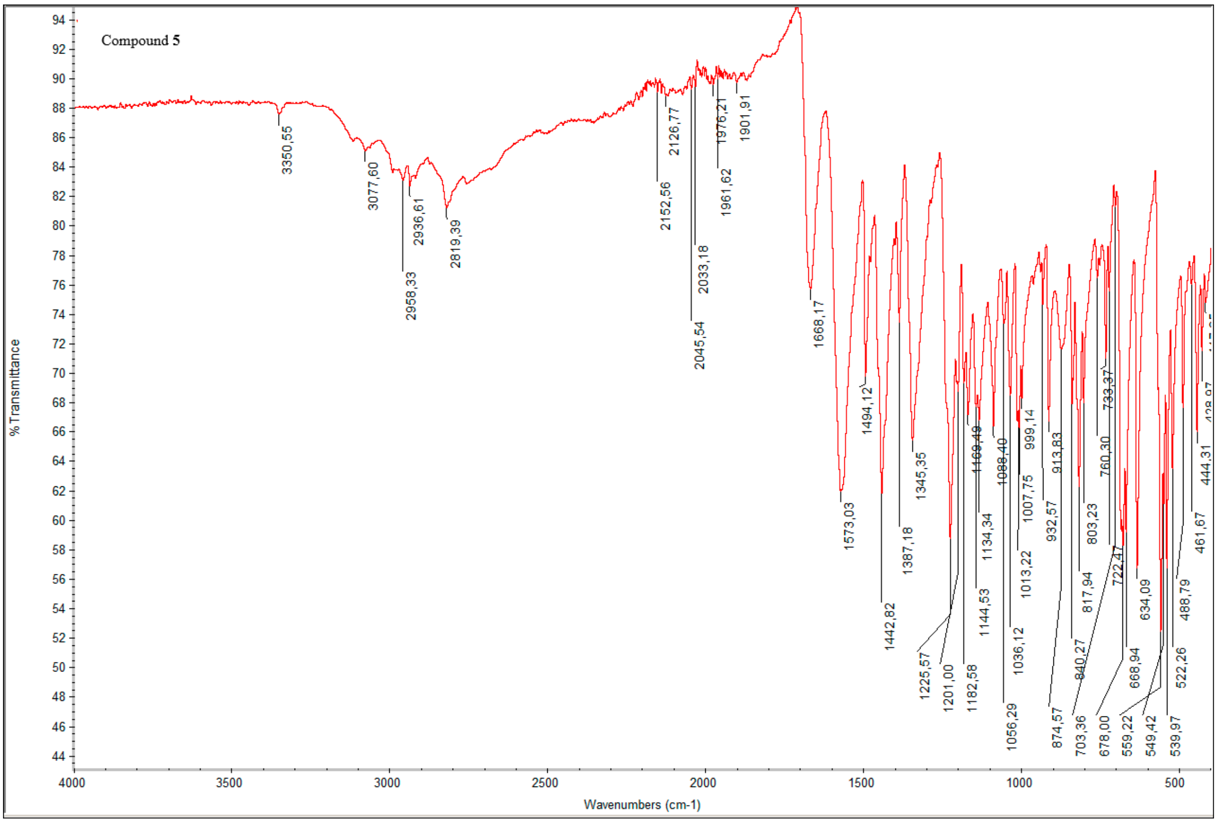

Figure S1. FT-IR spectrum of compound **5**.

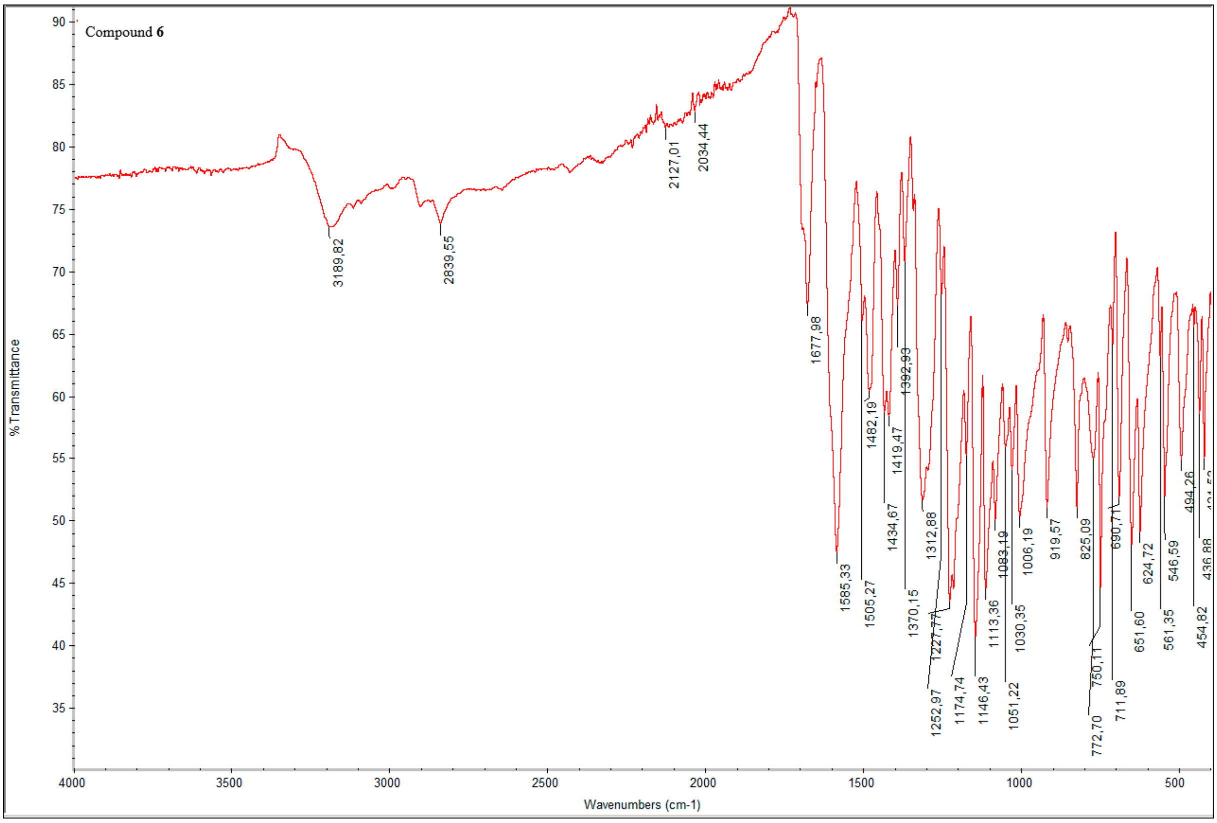

Figure S2. FT-IR spectrum of compound **6**.

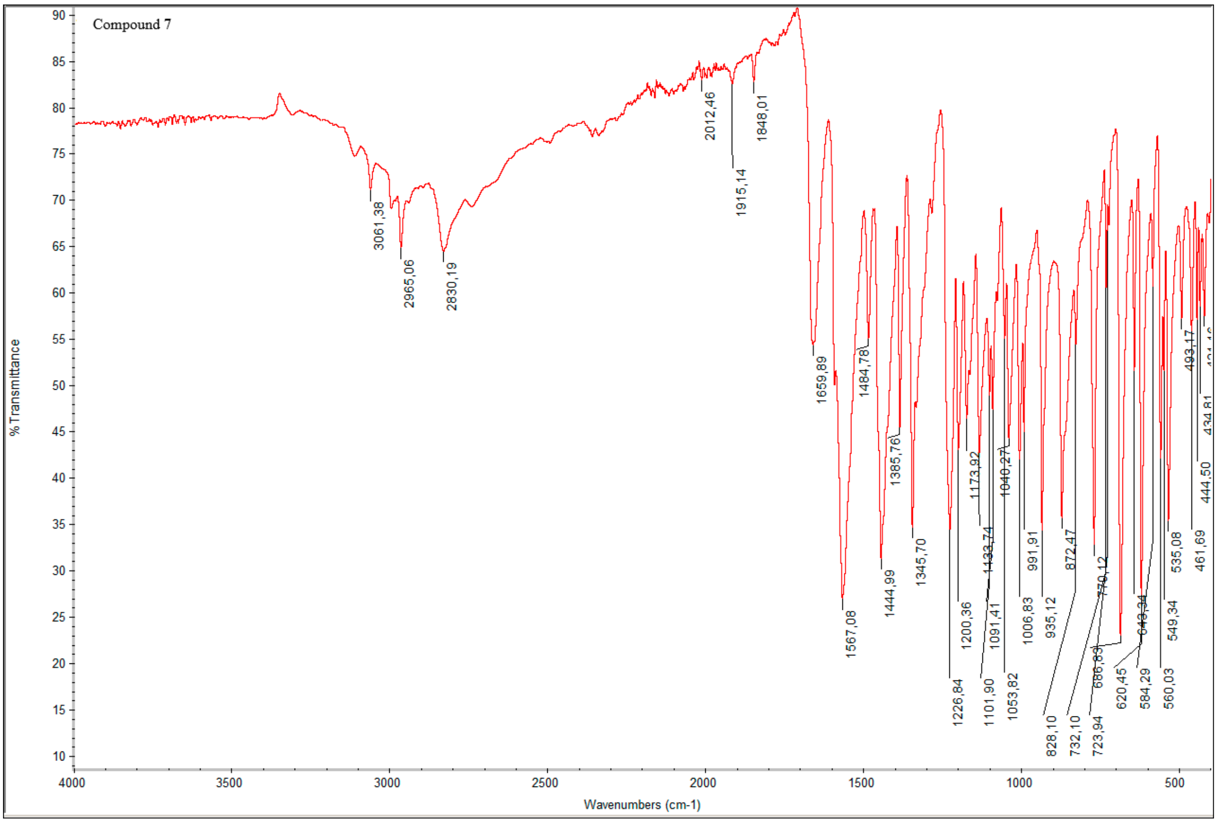

Figure S3. FT-IR spectrum of compound **7**.

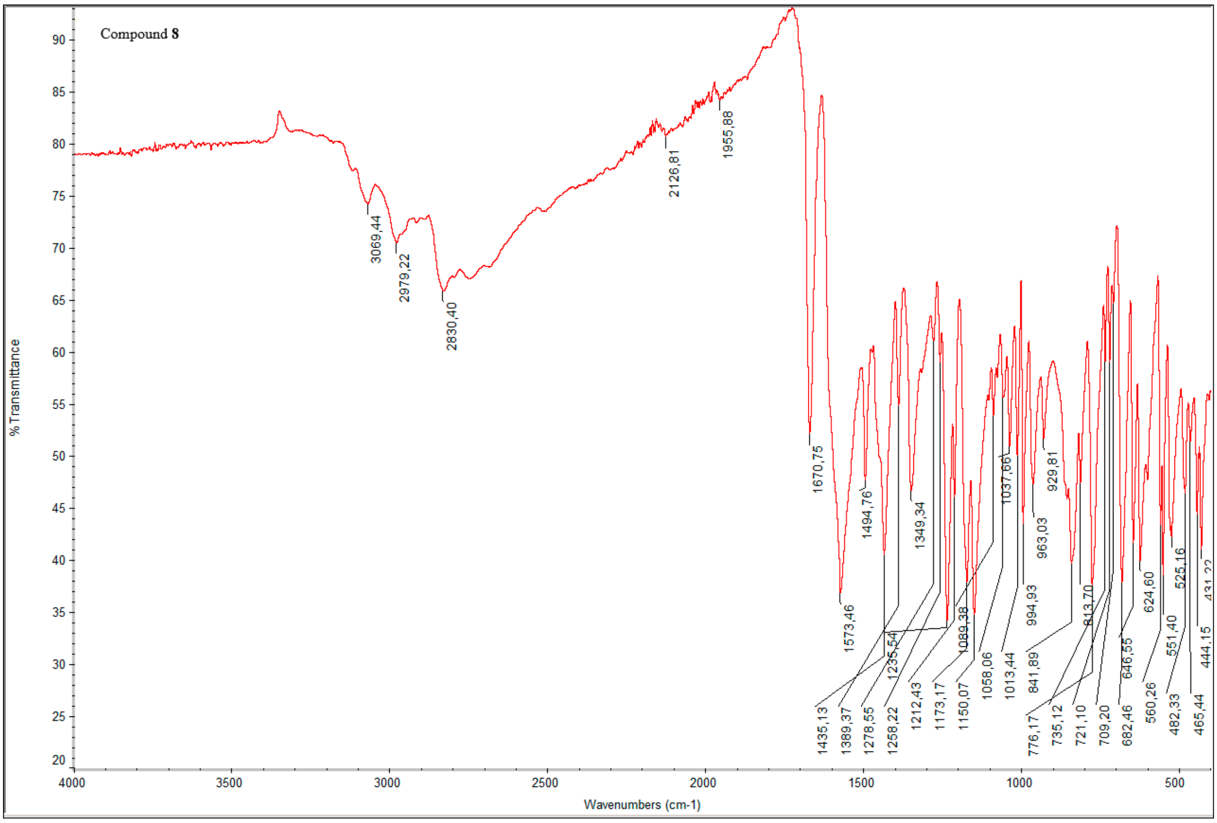

Figure S4. FT-IR spectrum of compound **8**.

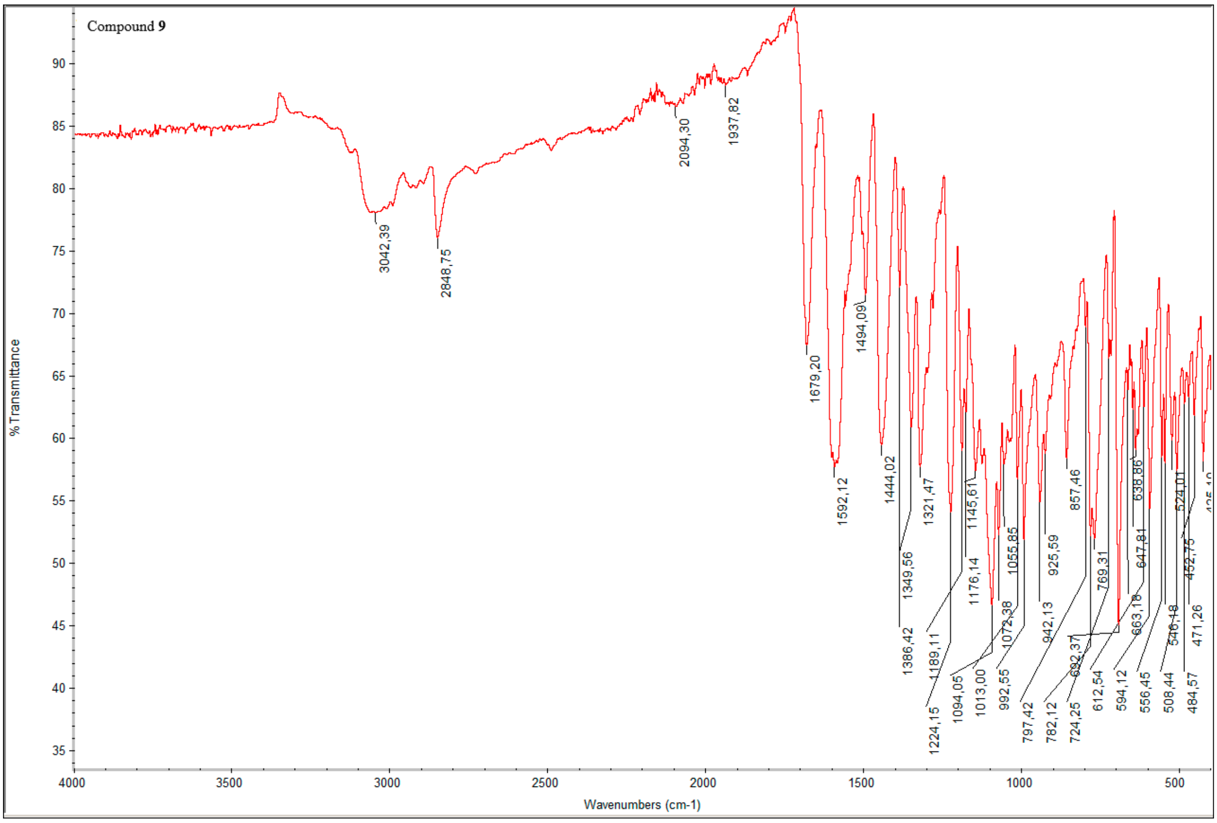

Figure S5. FT-IR spectrum of compound **9**.

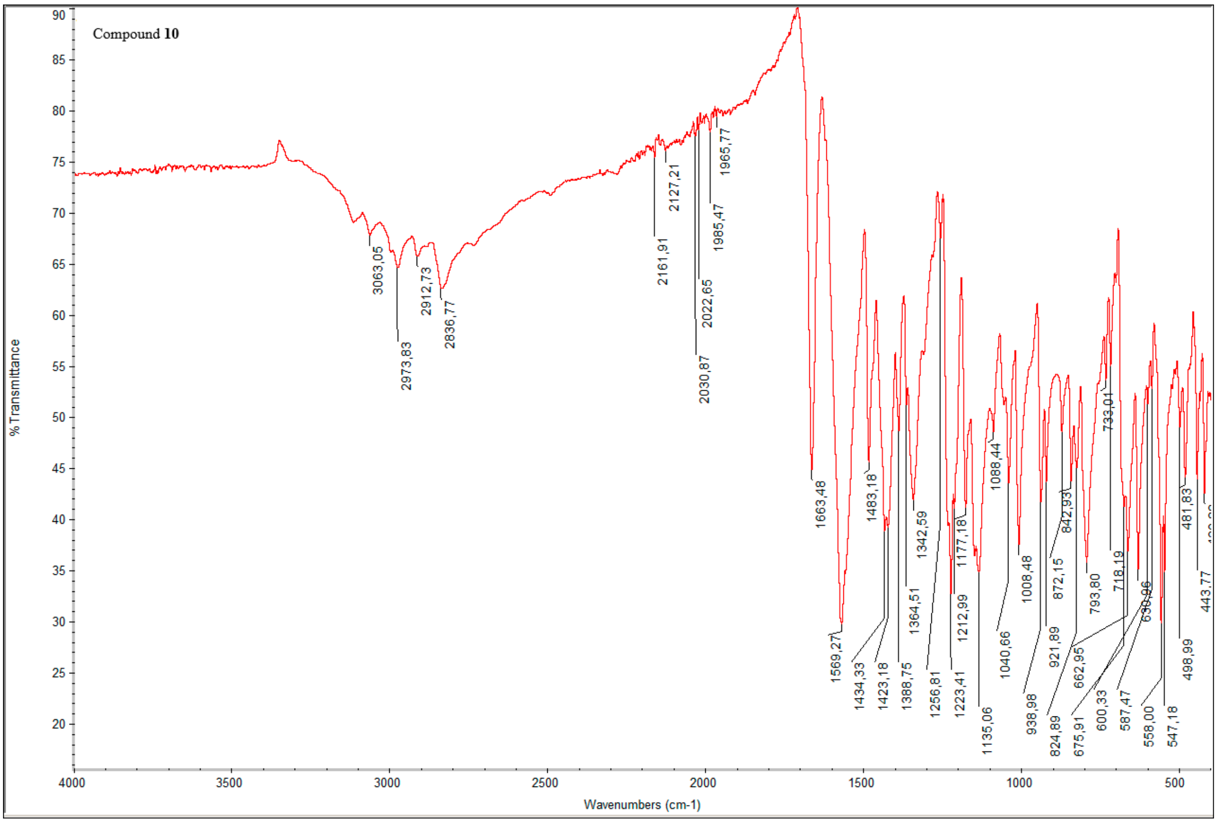

Figure S6. FT-IR spectrum of compound **10**.

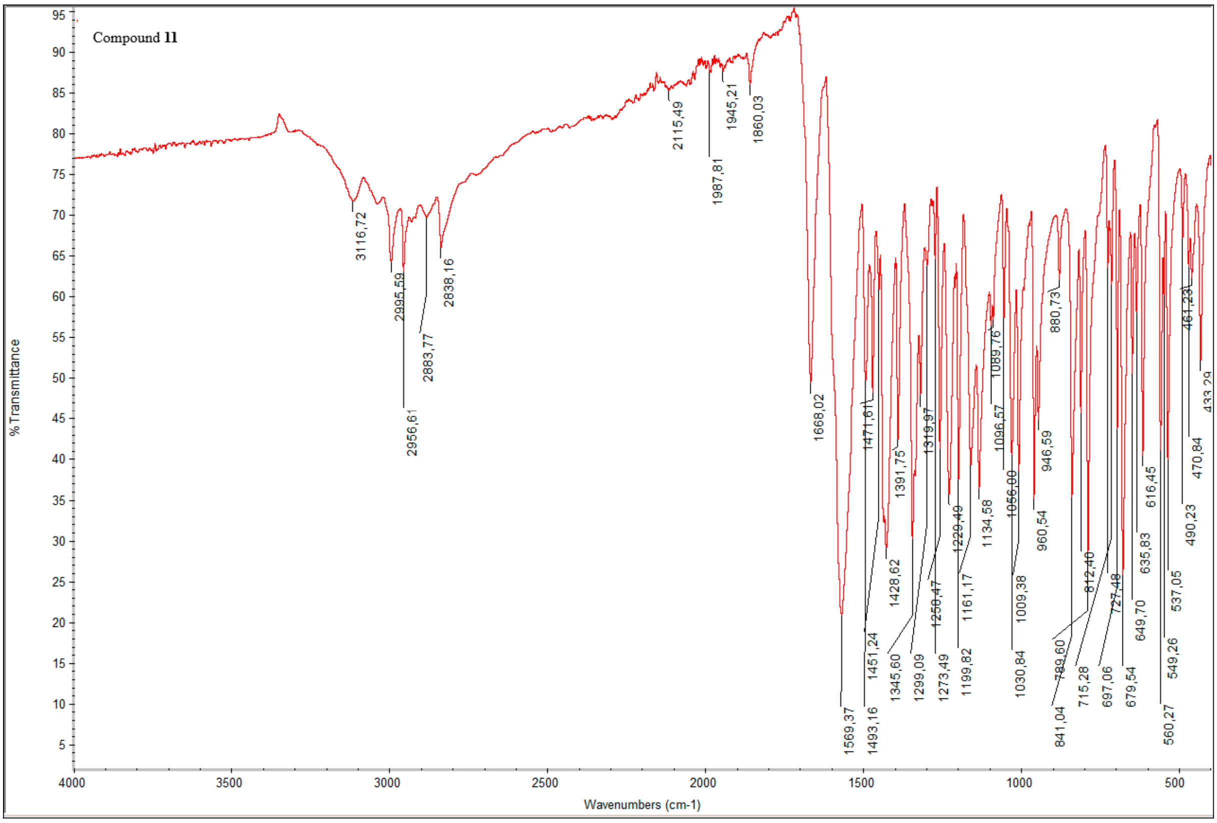

Figure S7. FT-IR spectrum of compound **11**.

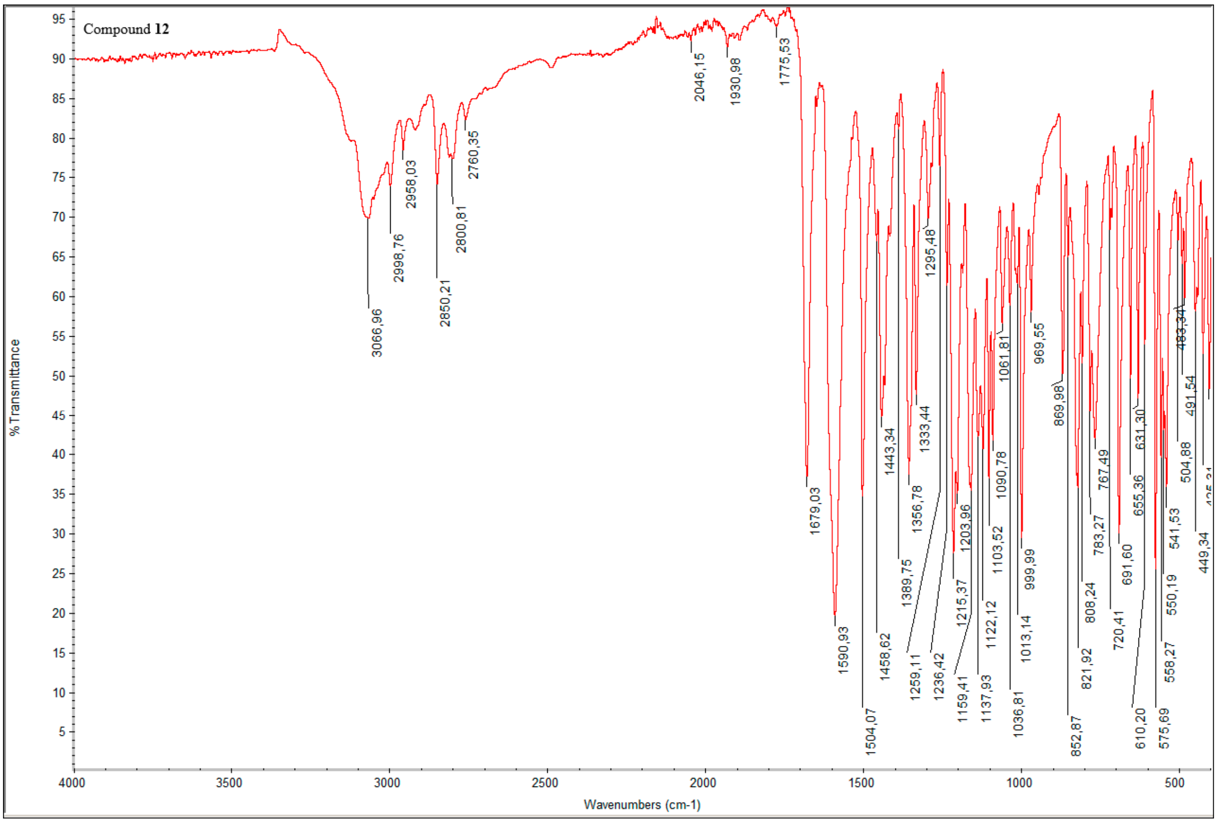

Figure S8. FT-IR spectrum of compound **12**.

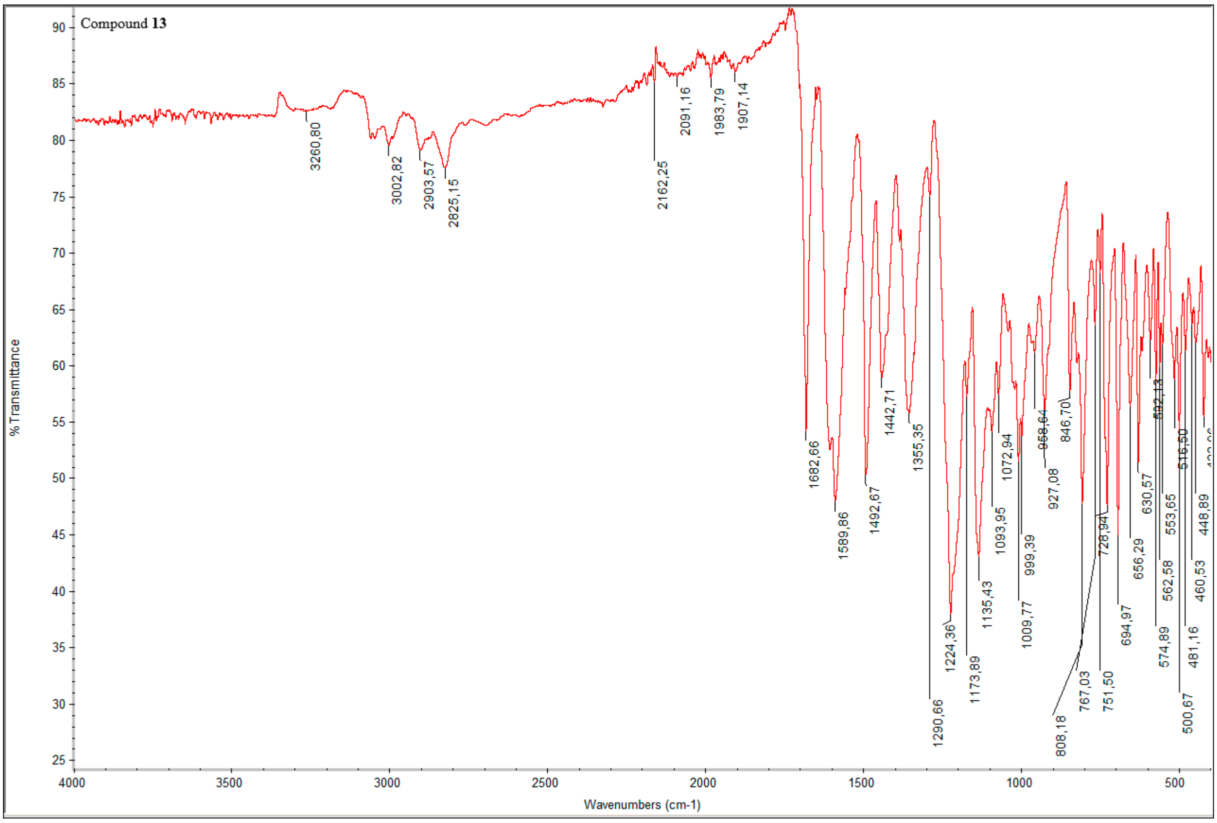

Figure S9. FT-IR spectrum of compound **13**.

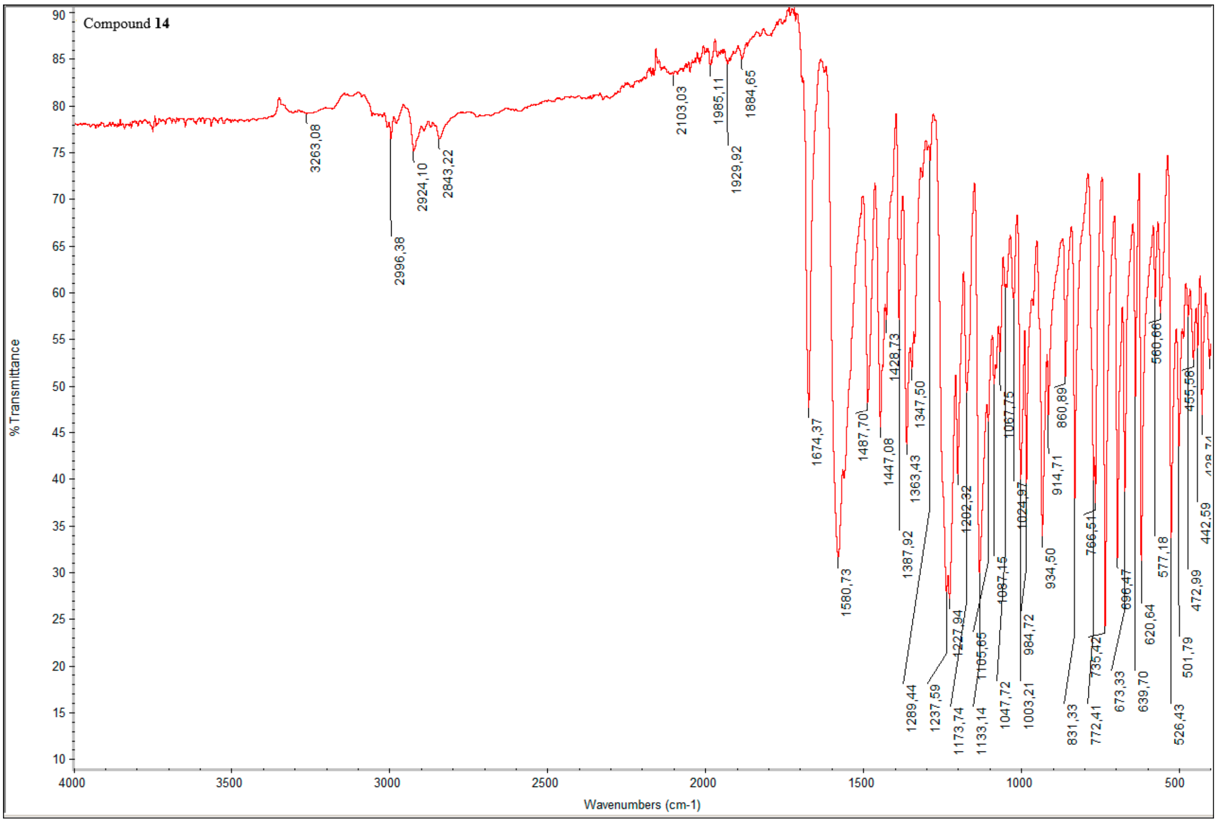

Figure S10. FT-IR spectrum of compound **14**.

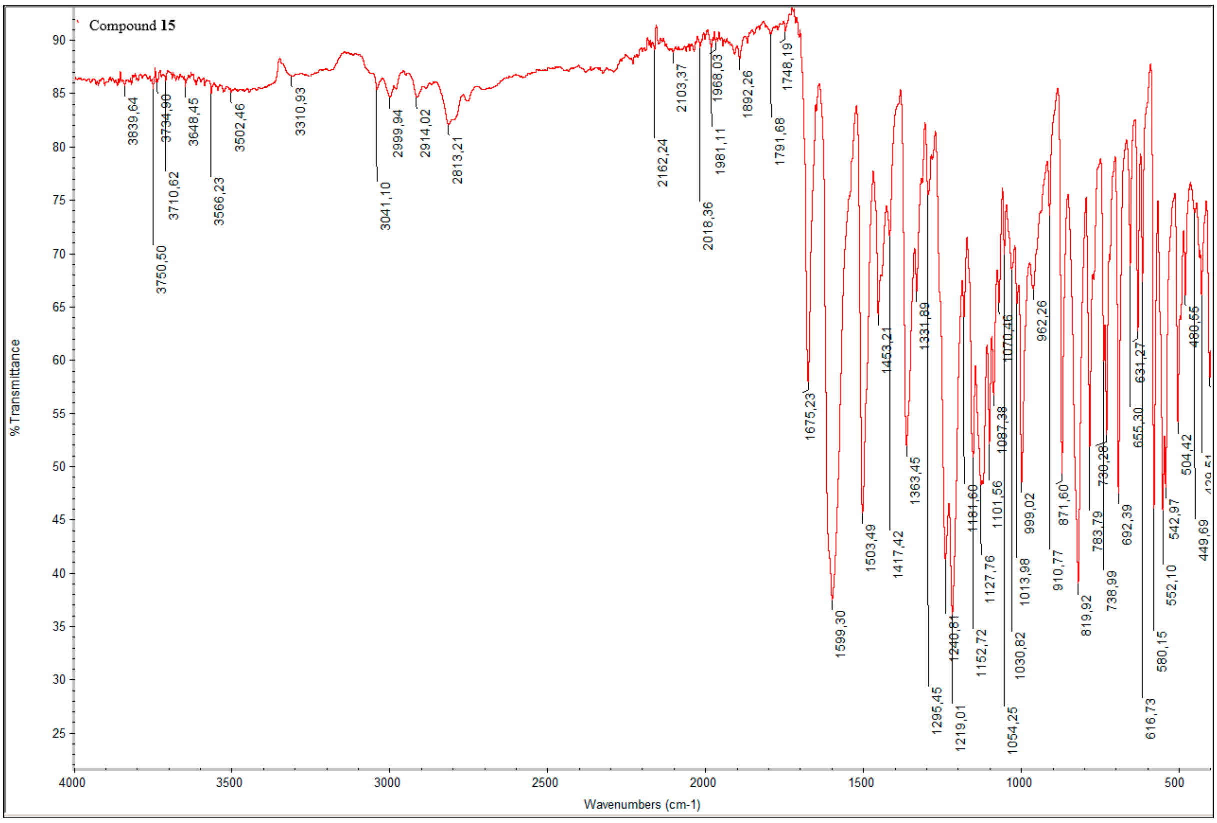

Figure S11. FT-IR spectrum of compound **15**.

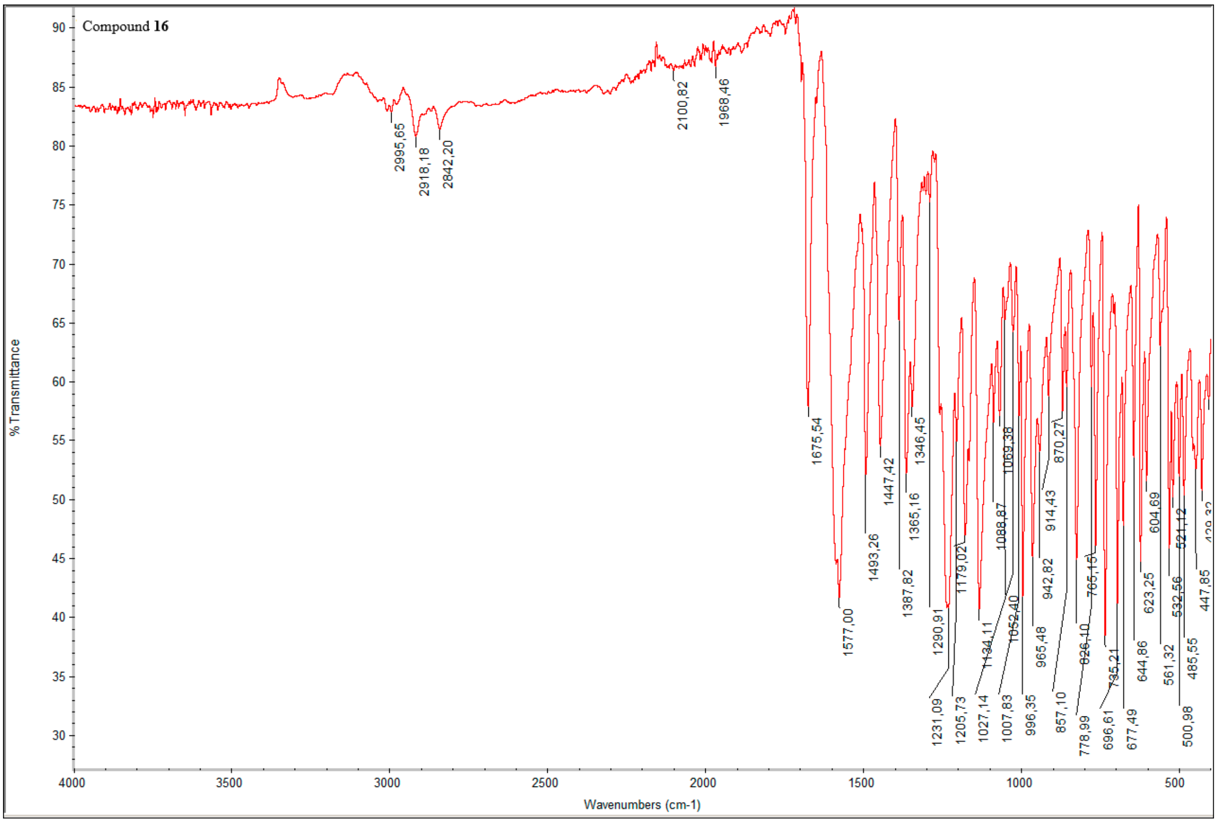

Figure S12. FT-IR spectrum of compound **16**.

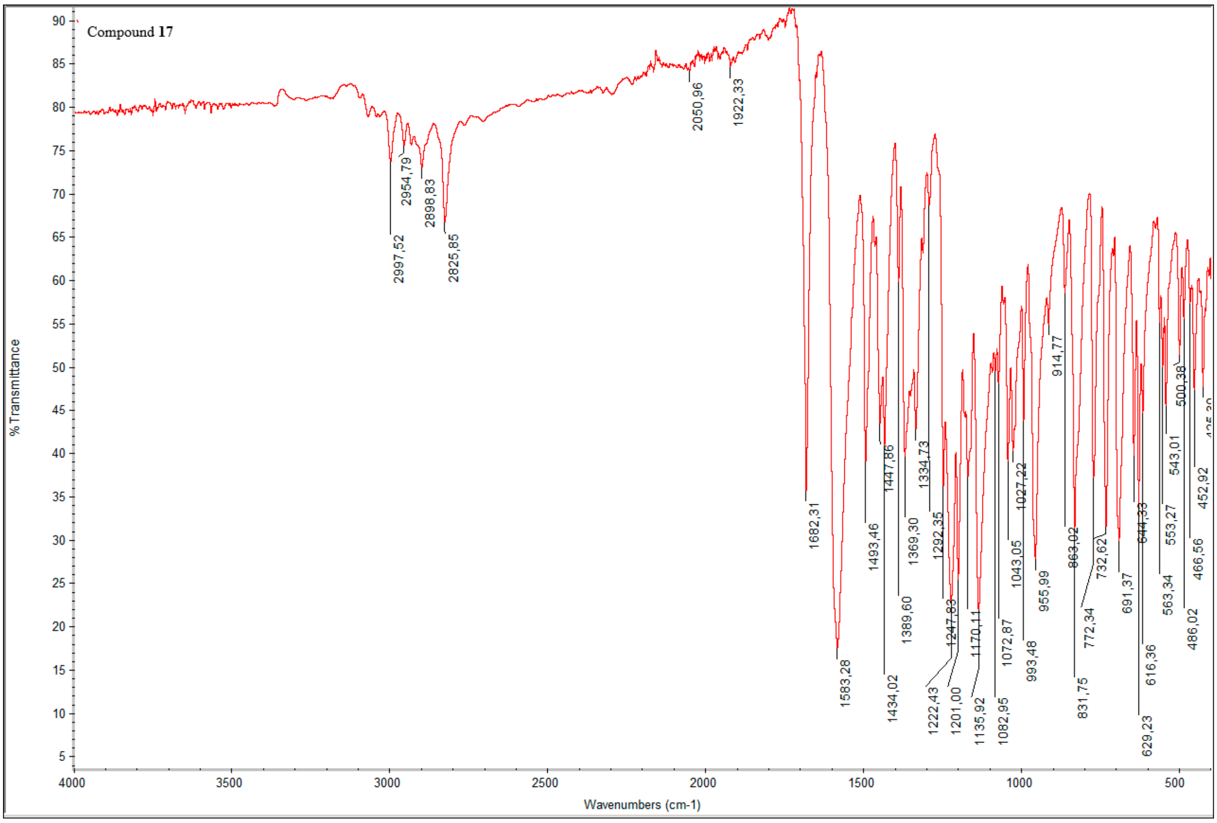

Figure S13. FT-IR spectrum of compound **17**.

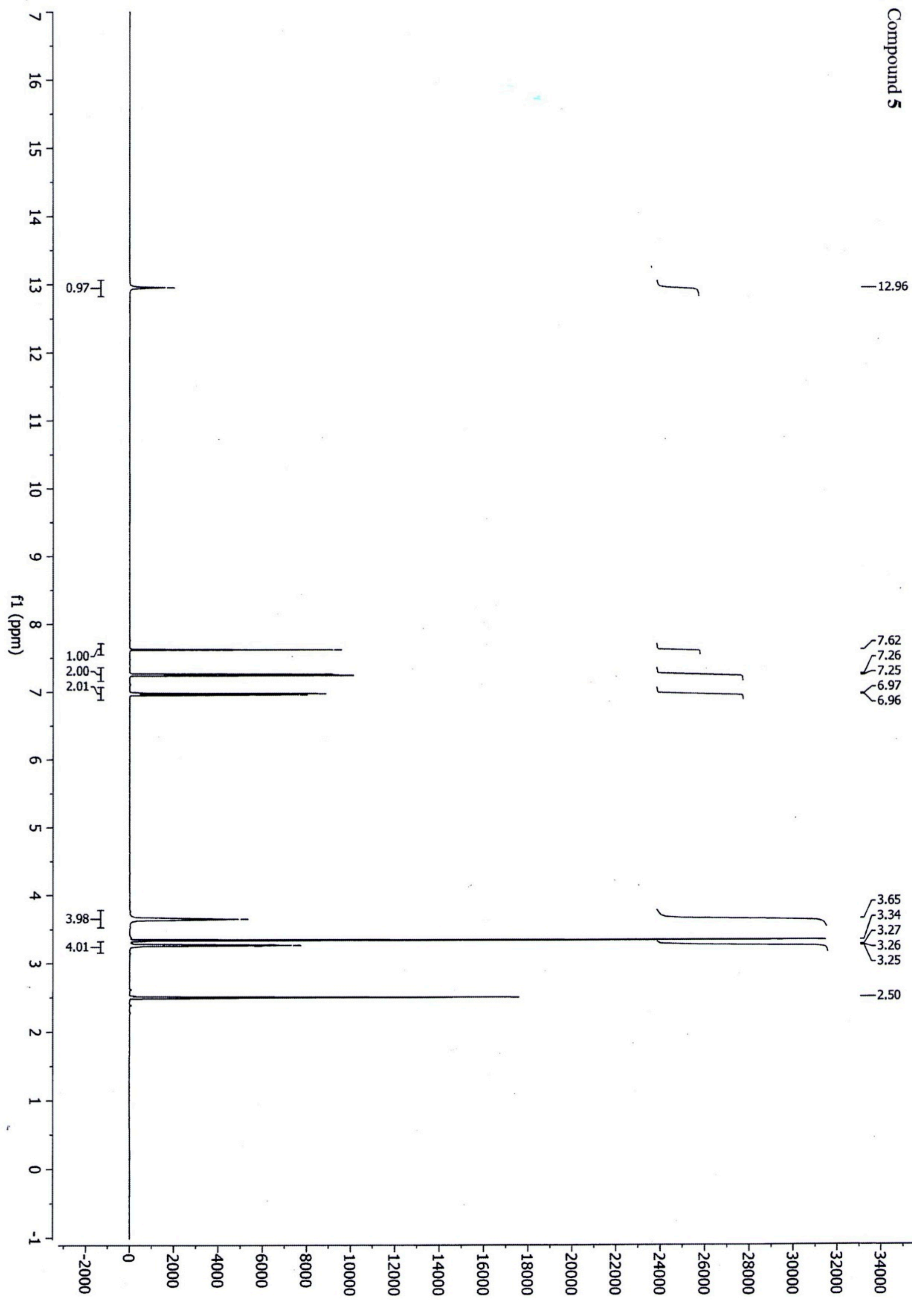

Figure S14. ¹H NMR spectrum of compound **5**.

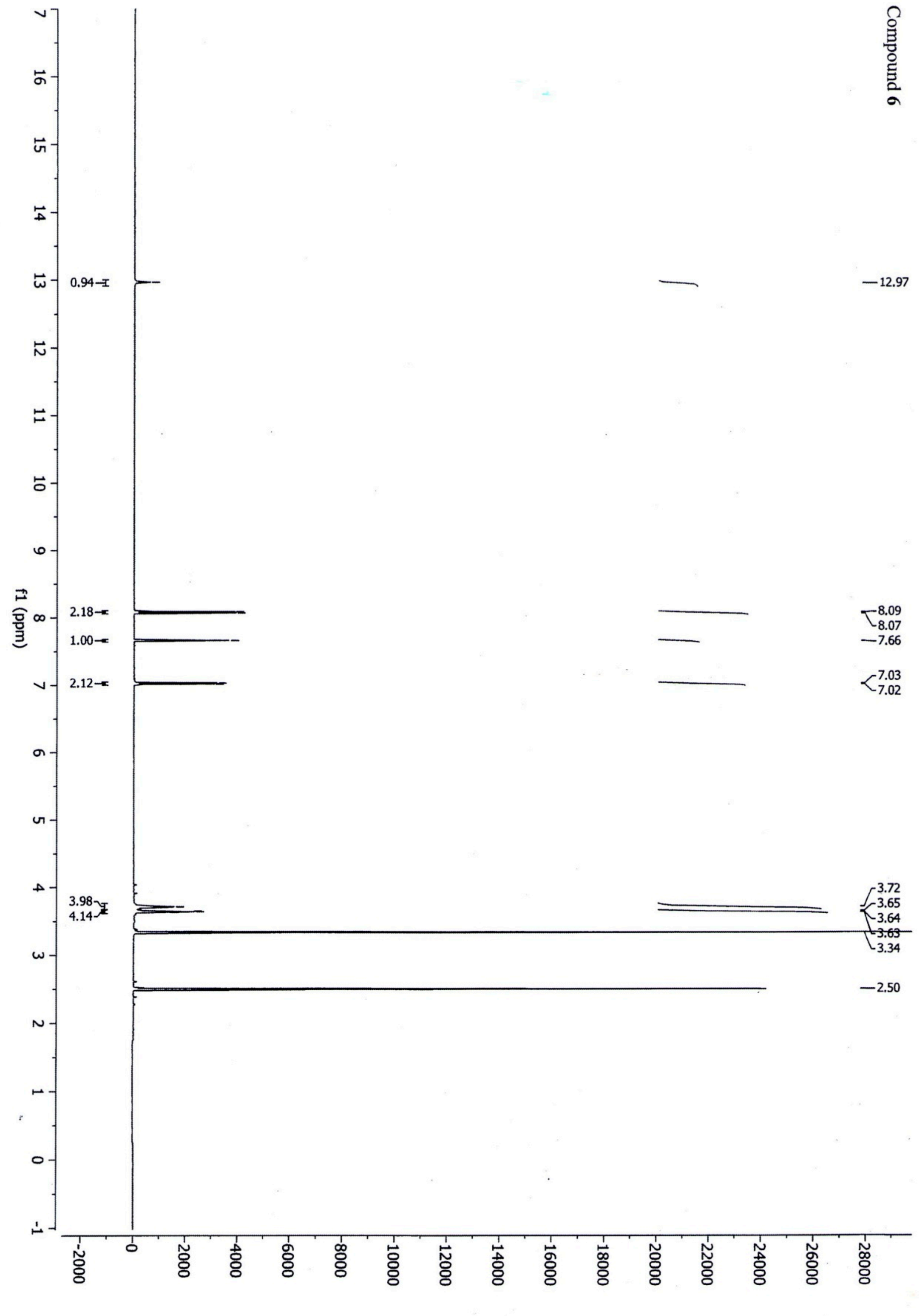

Figure S15. ^{1}H NMR spectrum of compound **6**.

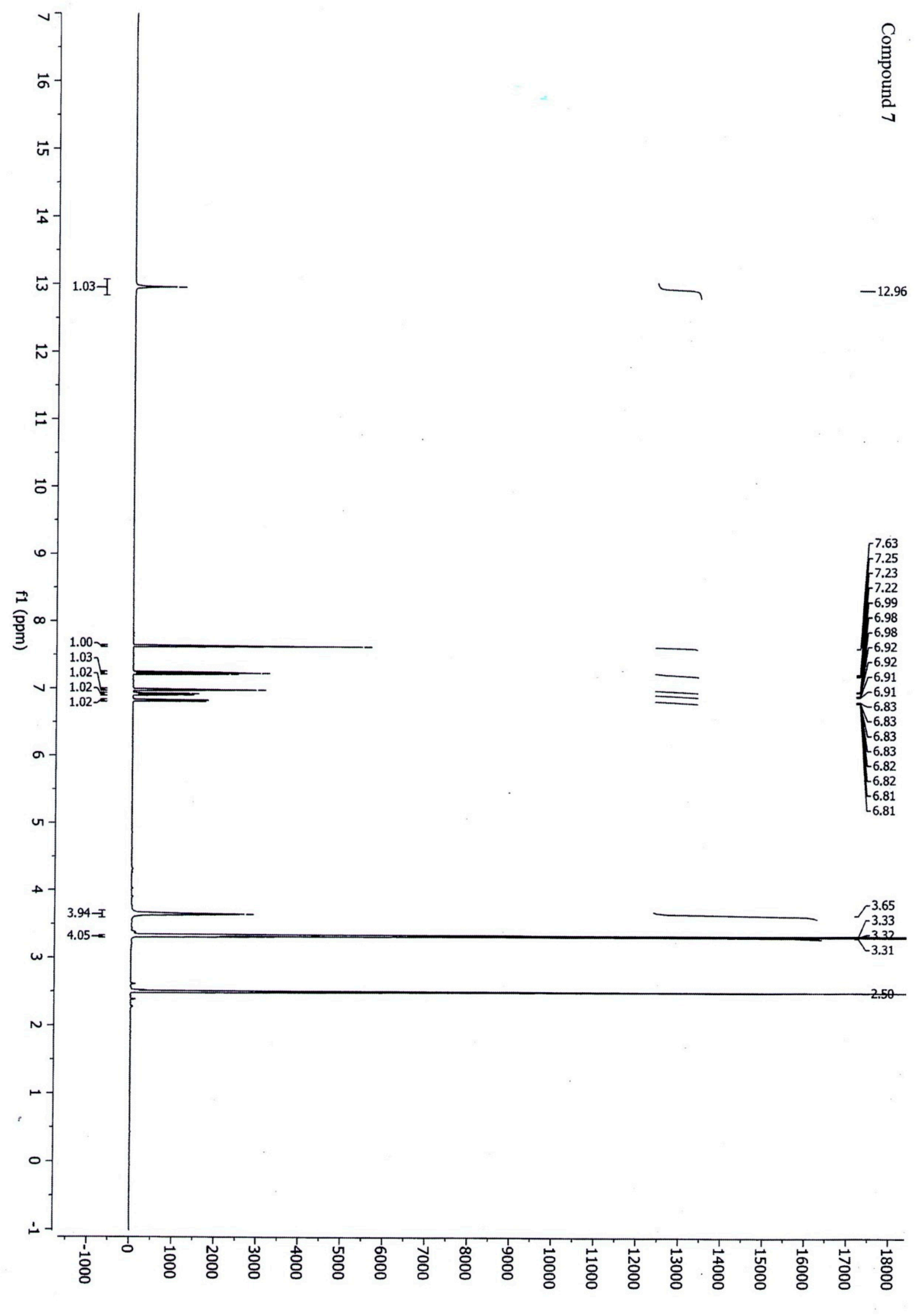

Figure S16. ^1H NMR spectrum of compound **7**.

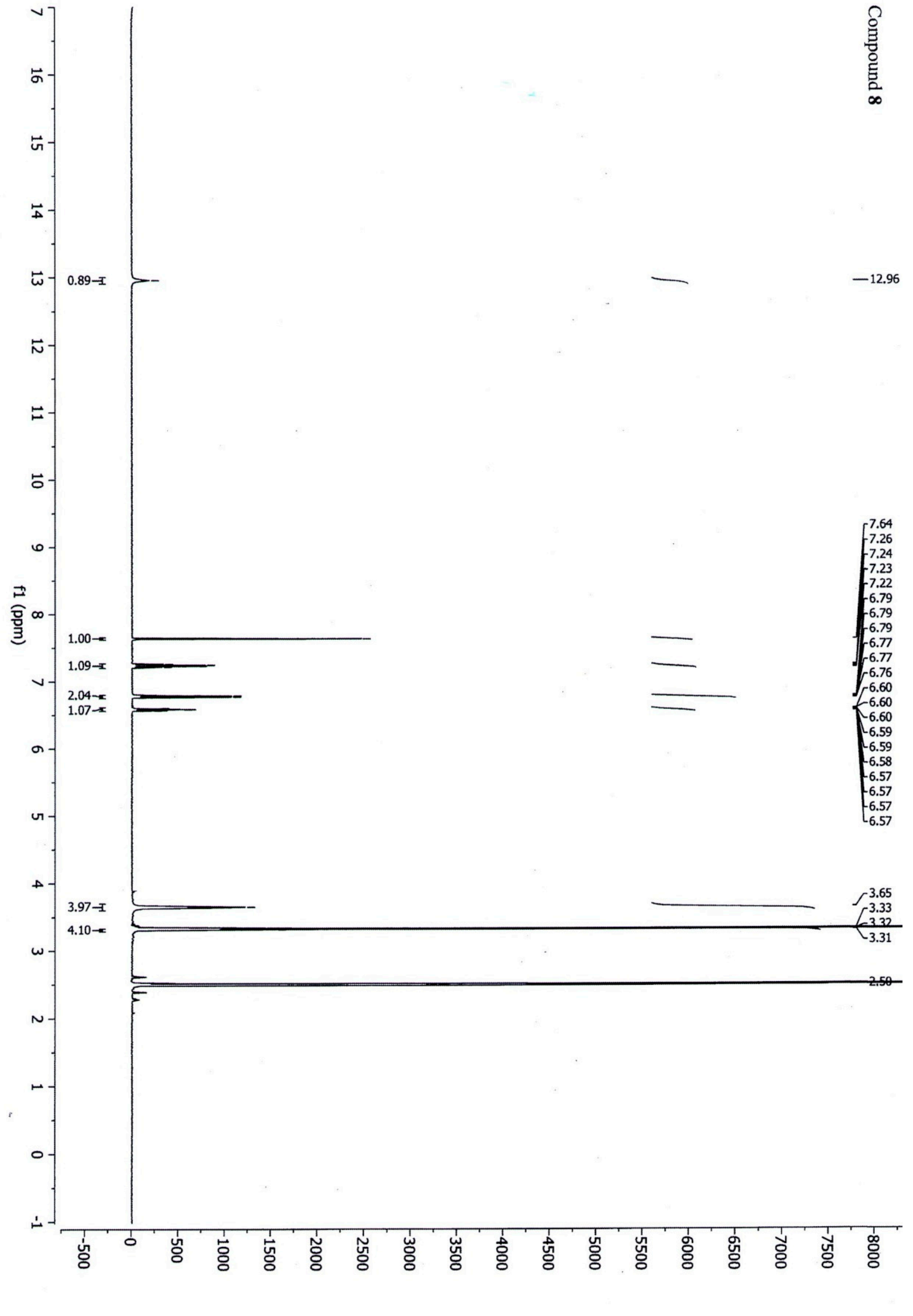

Figure S17. ^{1}H NMR spectrum of compound **8**.

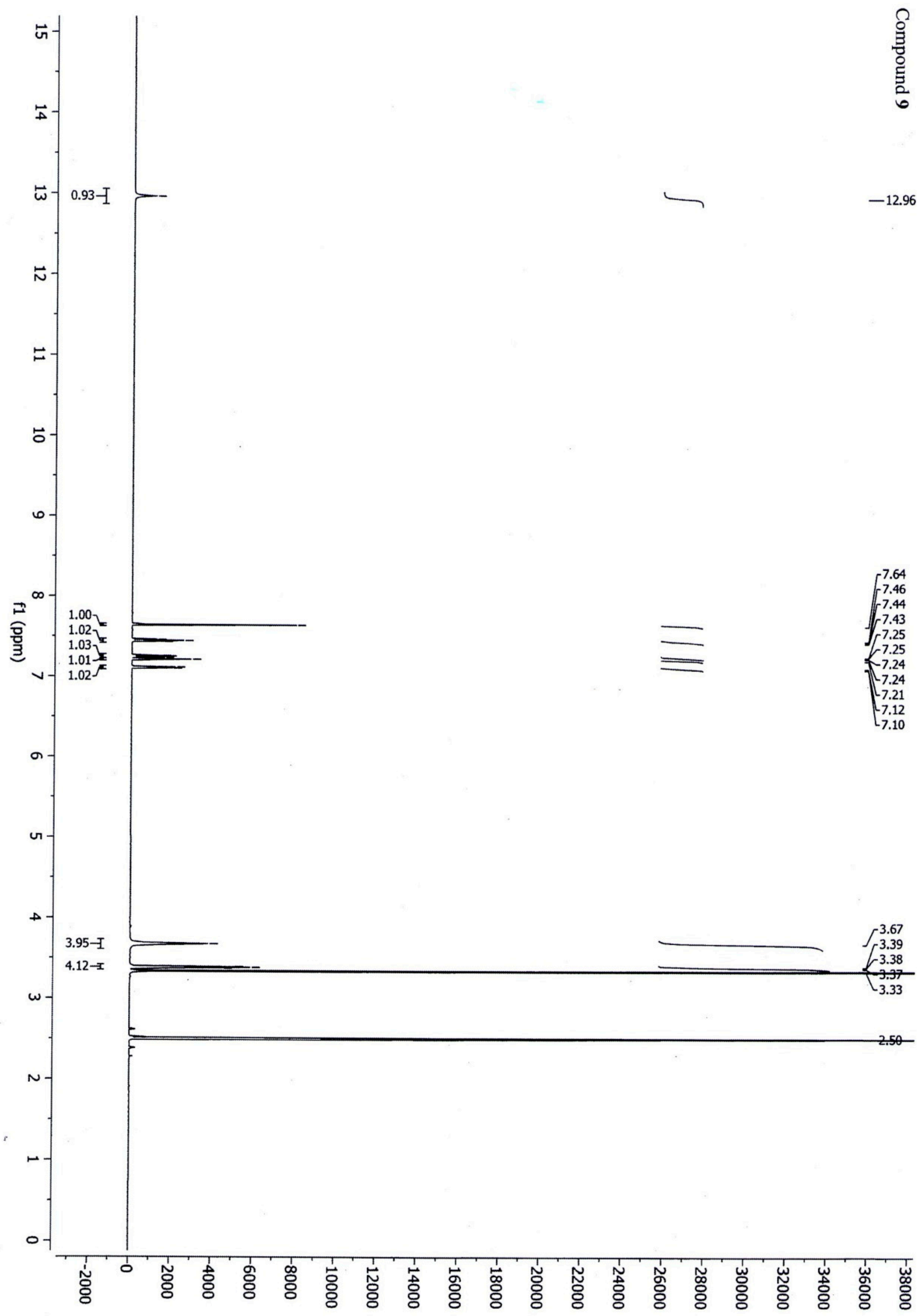

Figure S18. ^{1}H NMR spectrum of compound **9**.

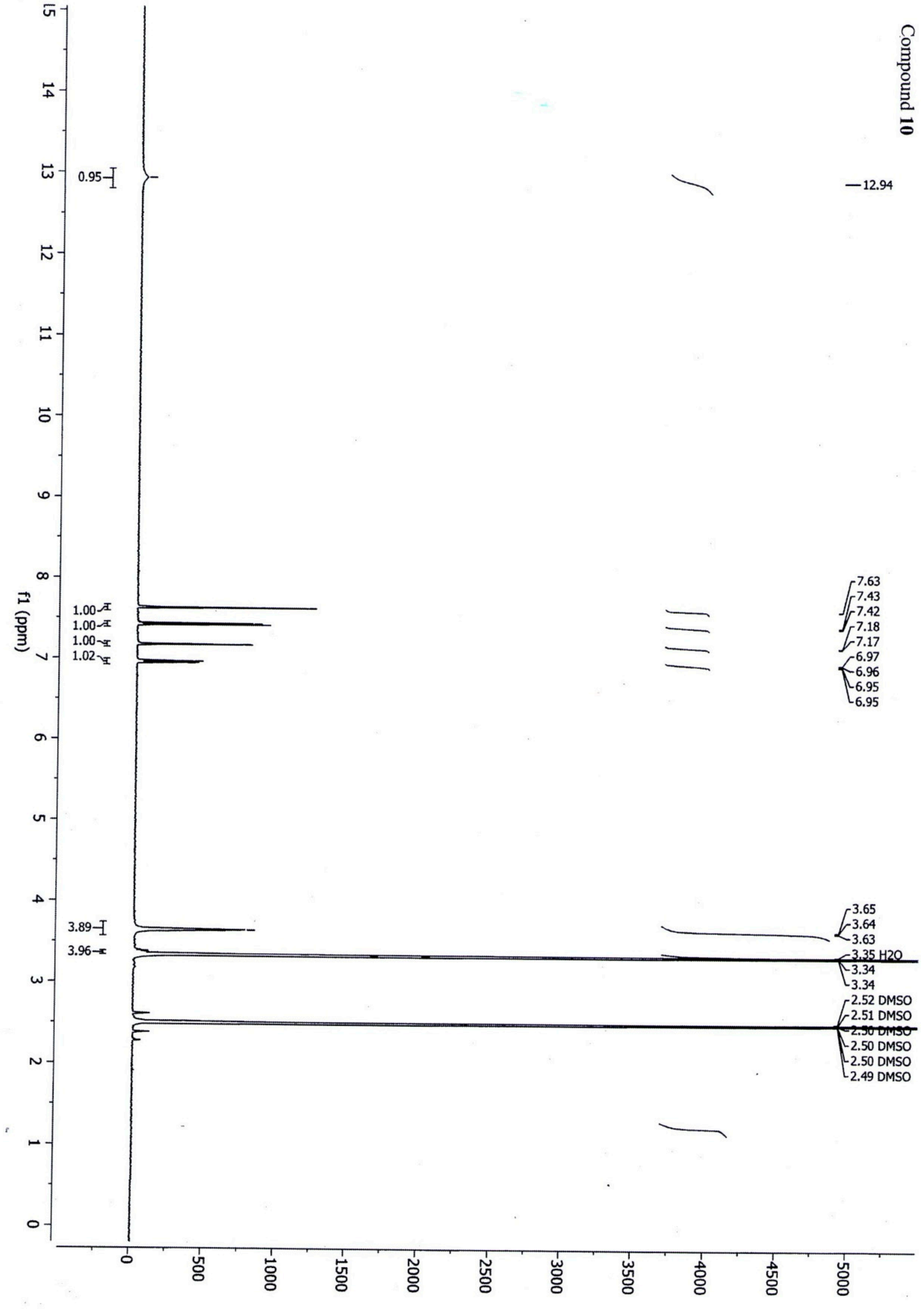

Figure S19. ^{1}H NMR spectrum of compound **10**.

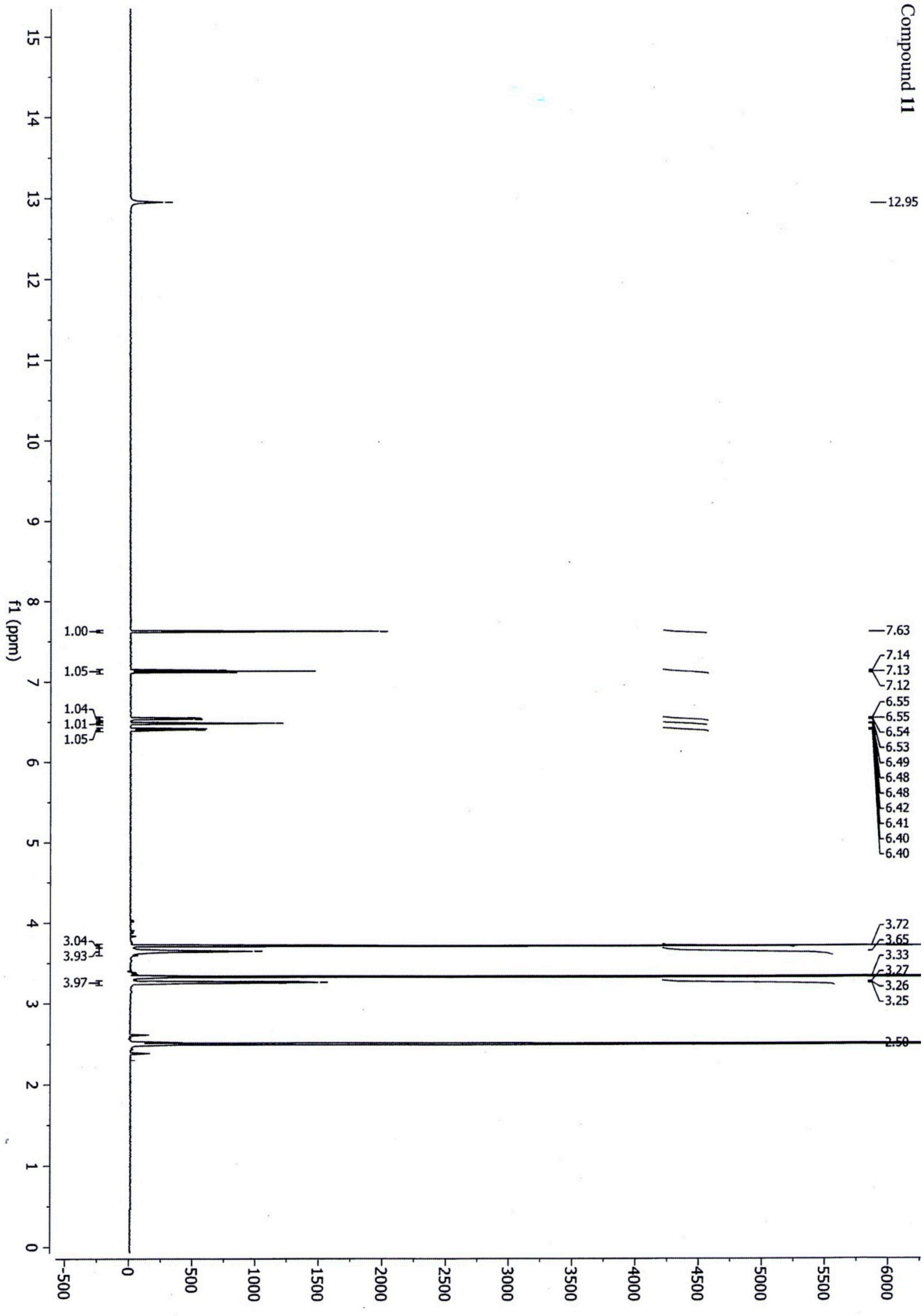

Figure S20. ^{1}H NMR spectrum of compound **11**.

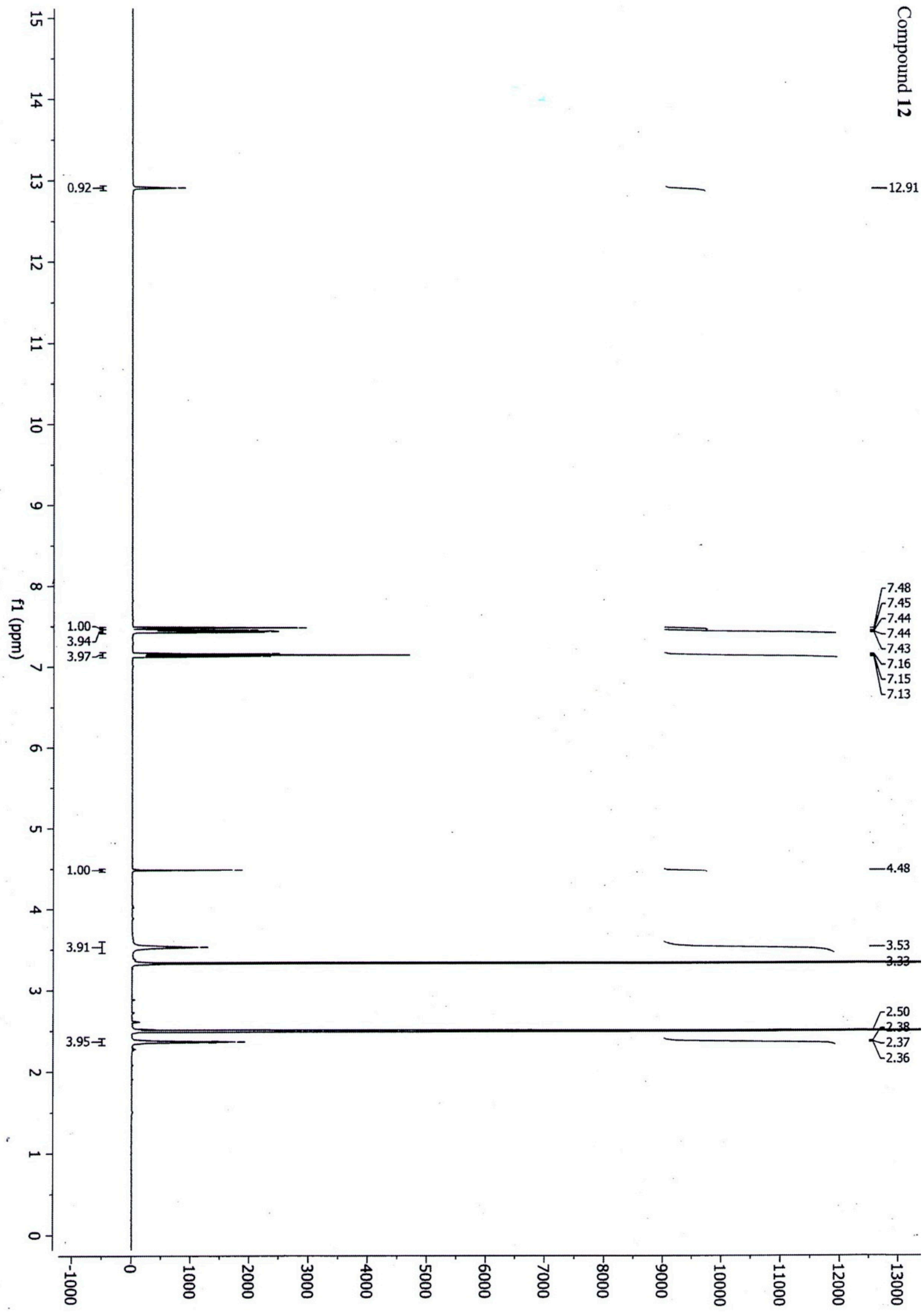

Figure S21. ^{1}H NMR spectrum of compound **12**.

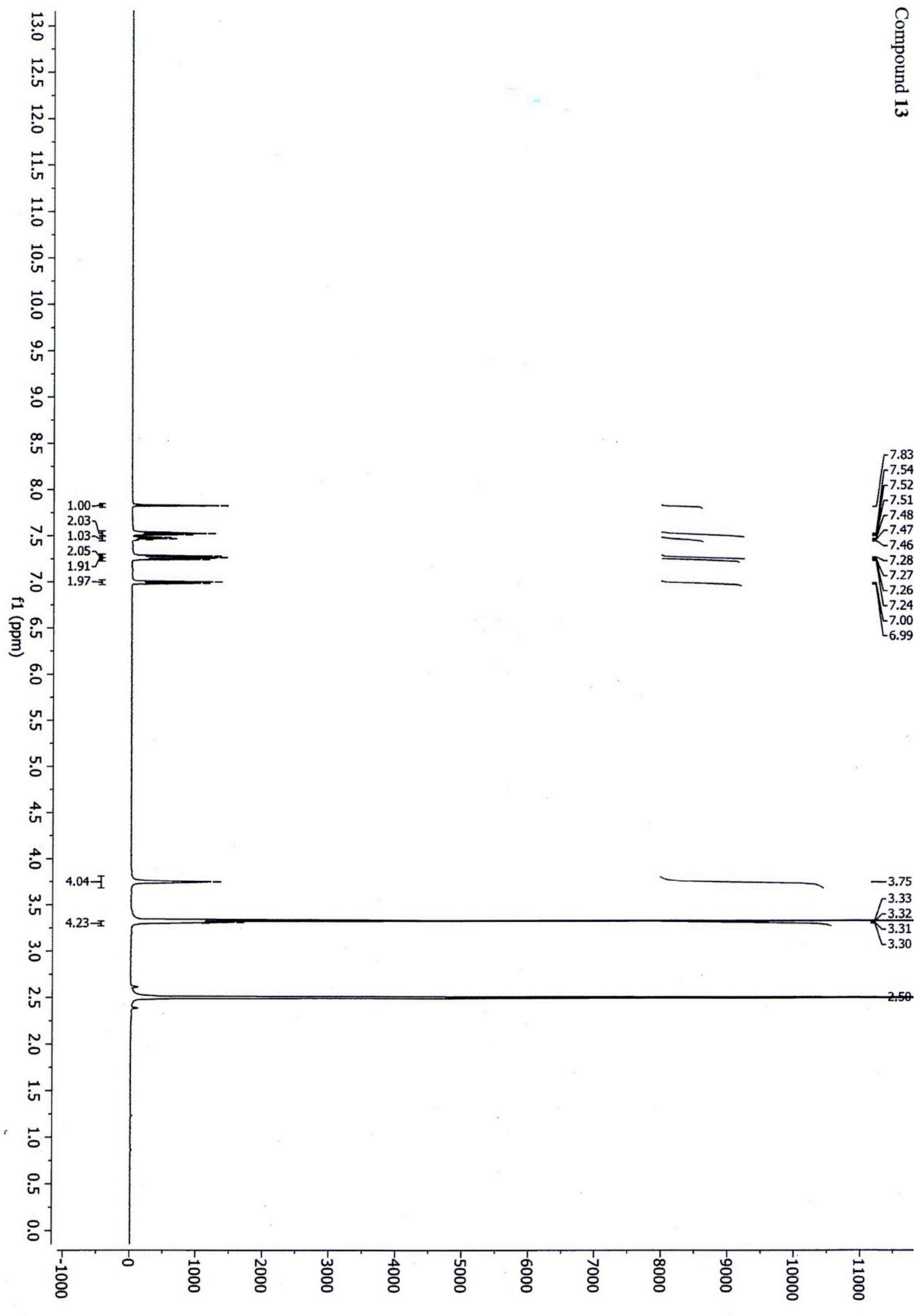

Figure S22. ^{1}H NMR spectrum of compound **13**.

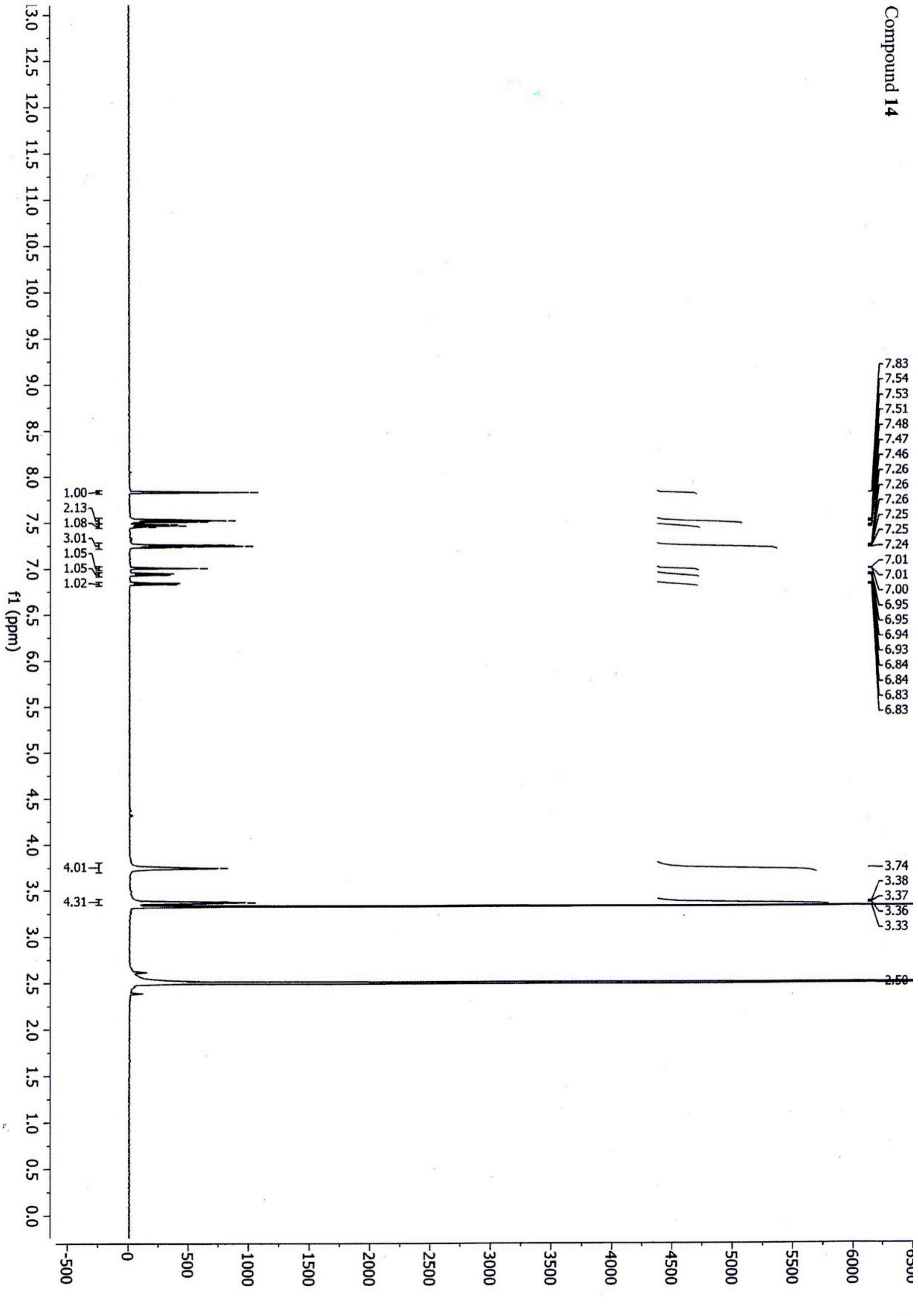

Figure S23. ^{1}H NMR spectrum of compound **14**.

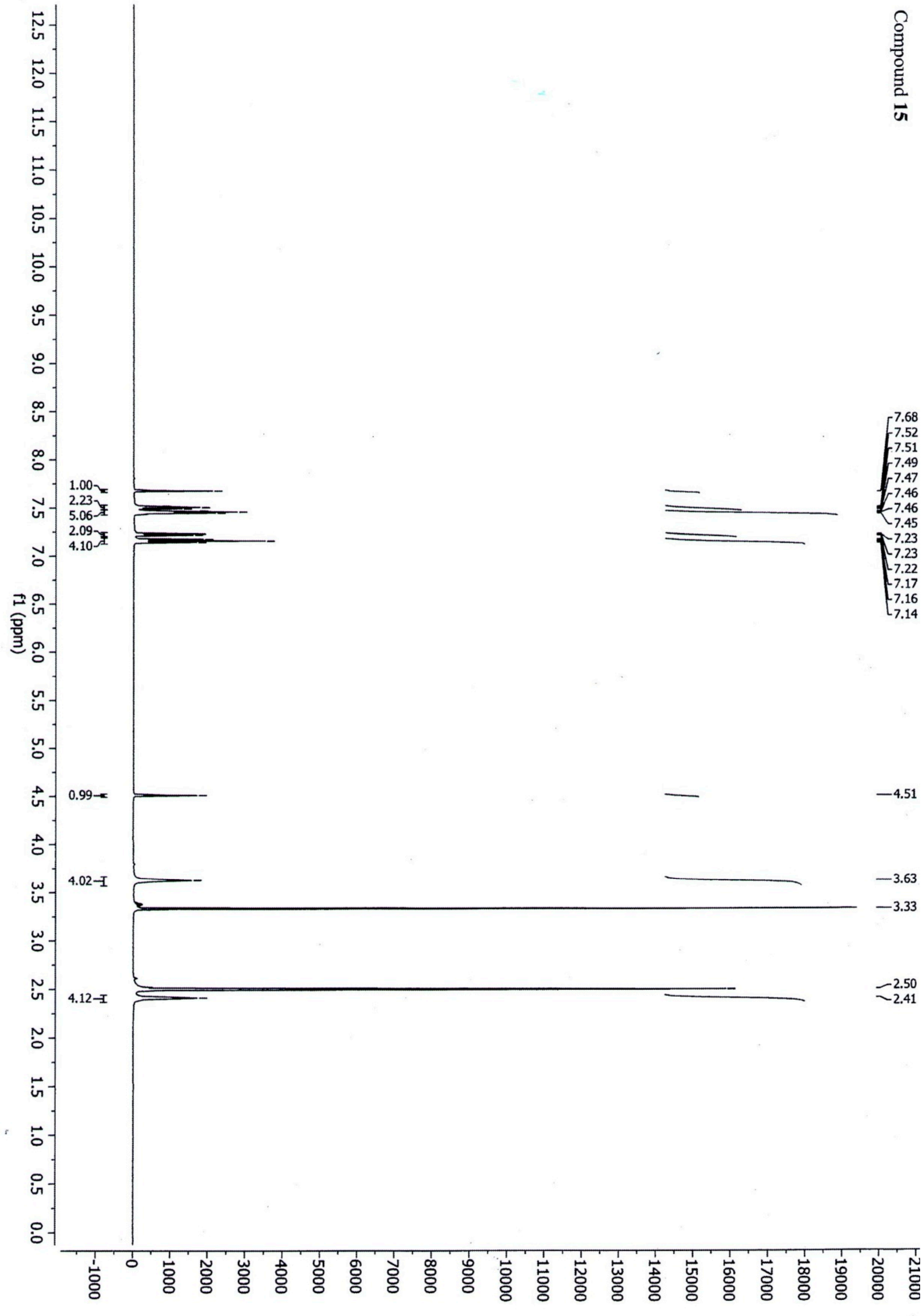

Figure S24. ^{1}H NMR spectrum of compound **15**.

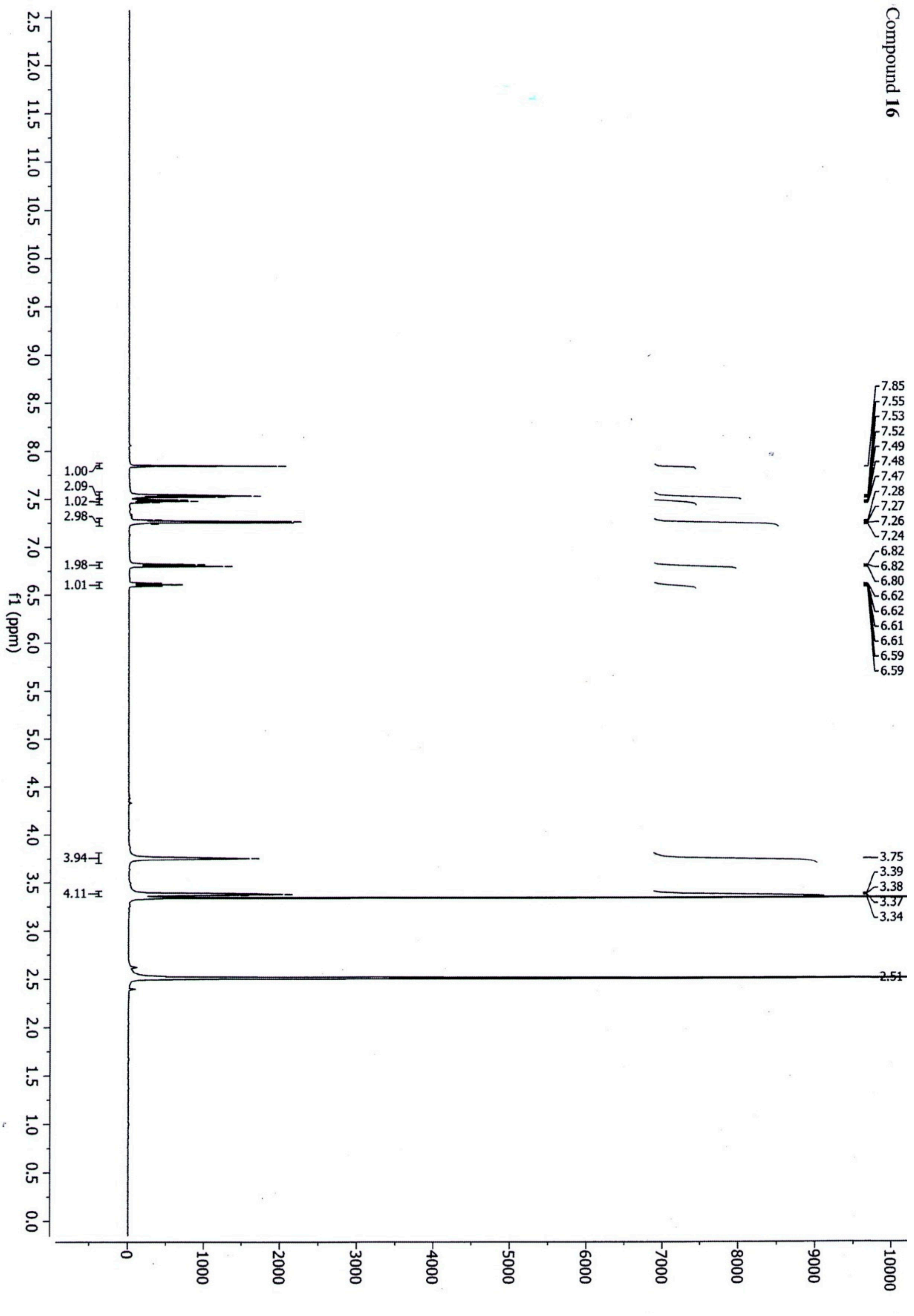

Figure S25. ^{1}H NMR spectrum of compound **16**.

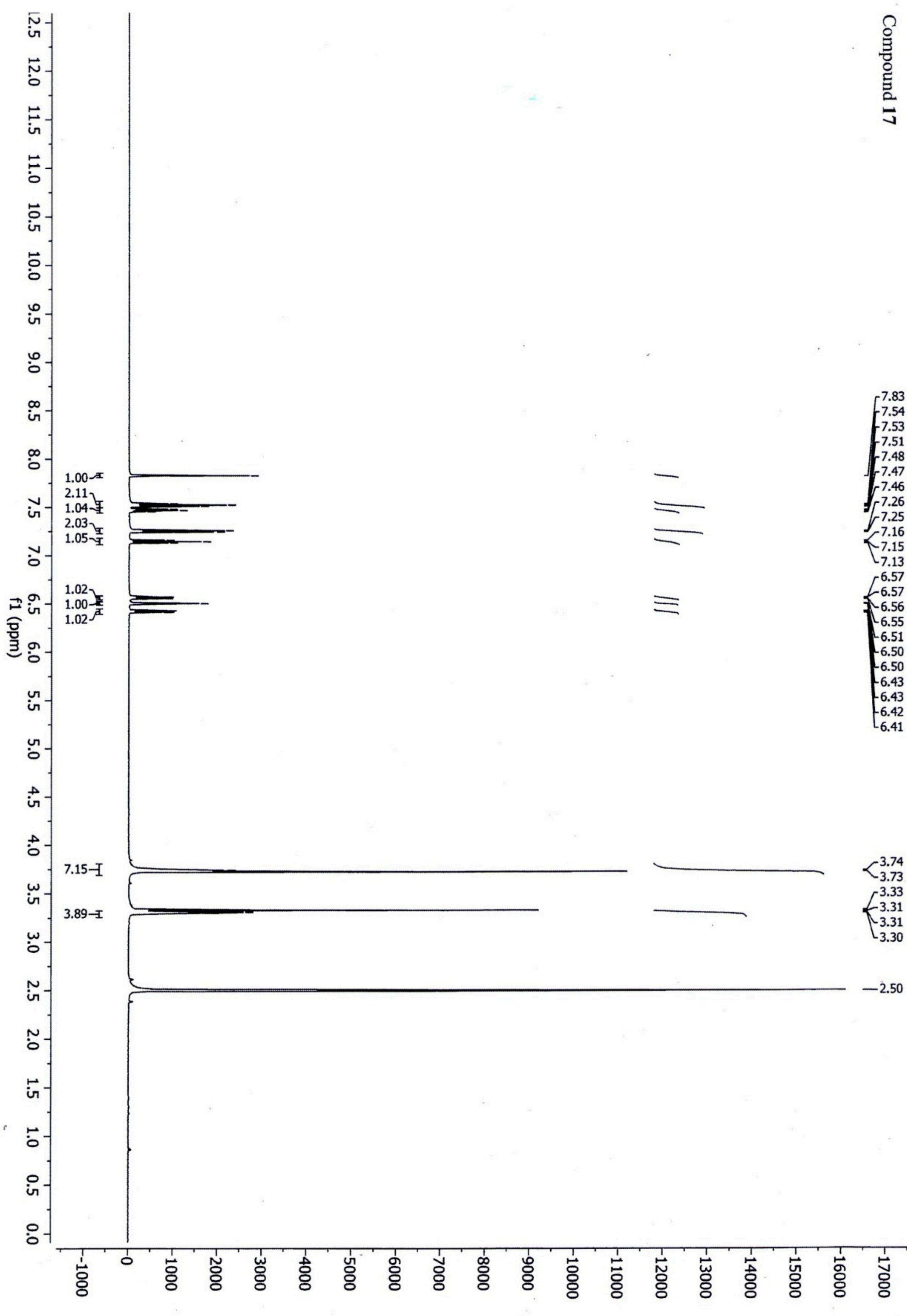

Figure S26. ^{1}H NMR spectrum of compound **17**.

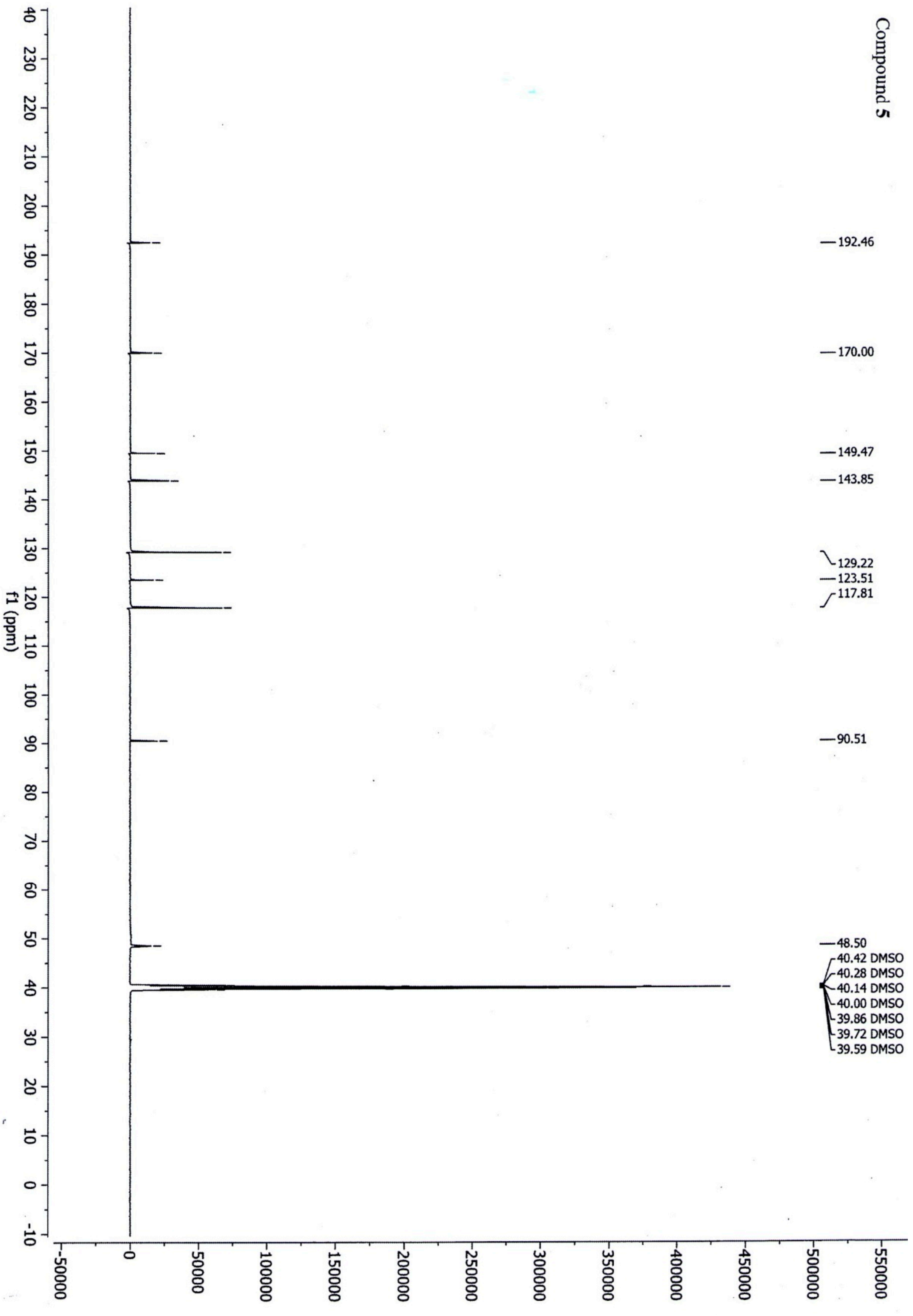

Figure S27. ^{13}C NMR spectrum of compound **5**.

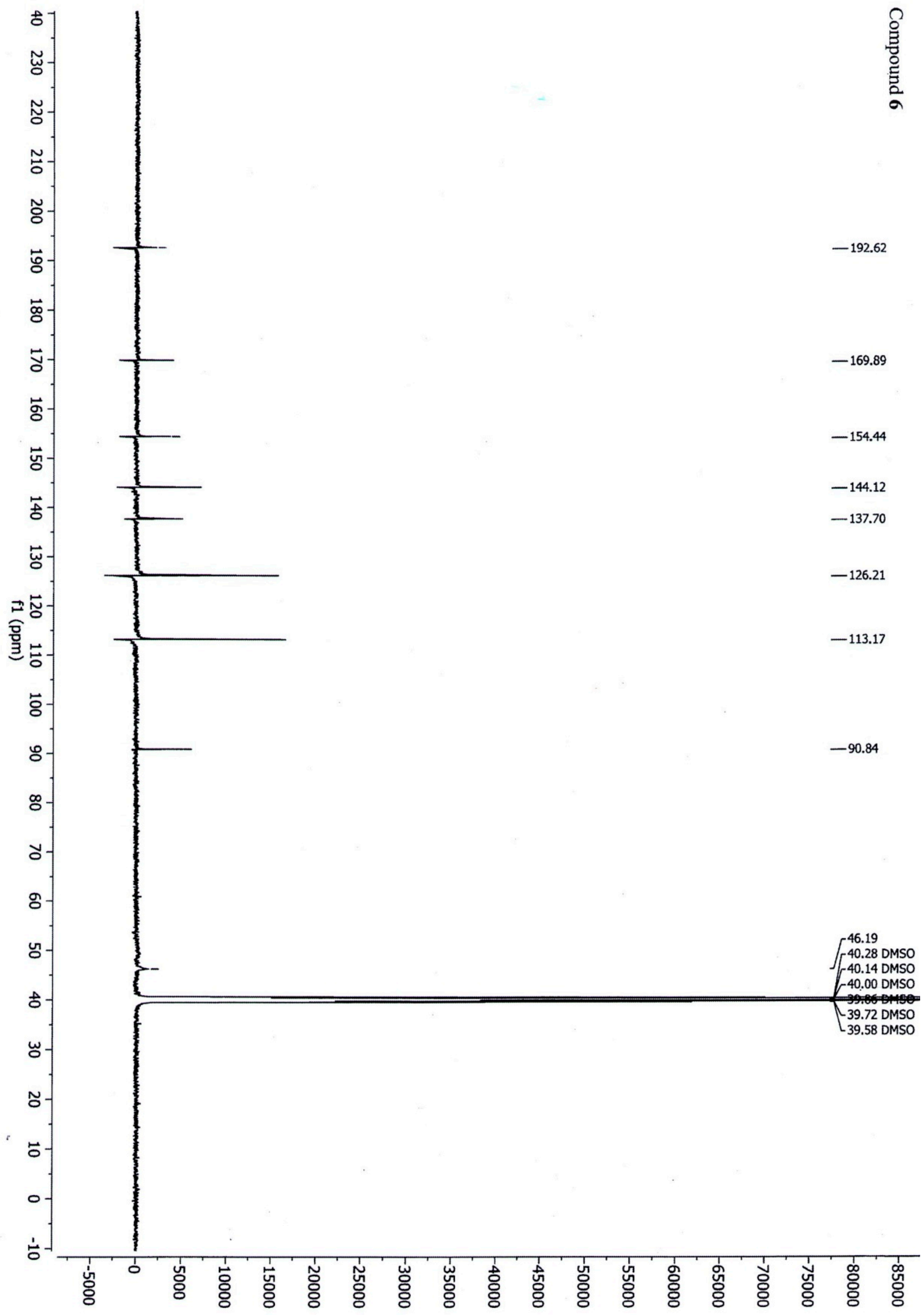

Figure S28. ^{13}C NMR spectrum of compound **6**.

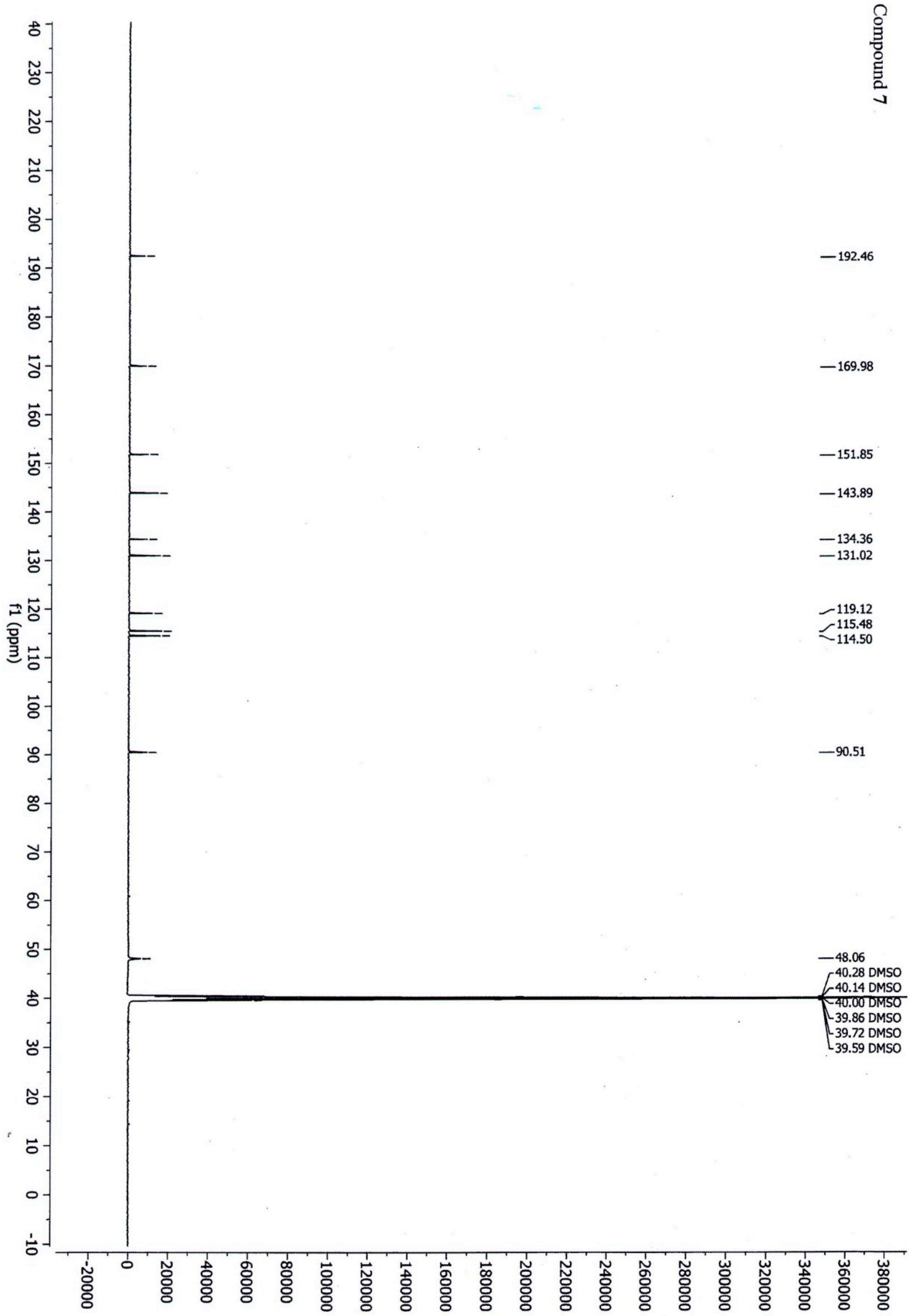

Figure S29. ^{13}C NMR spectrum of compound **7**.

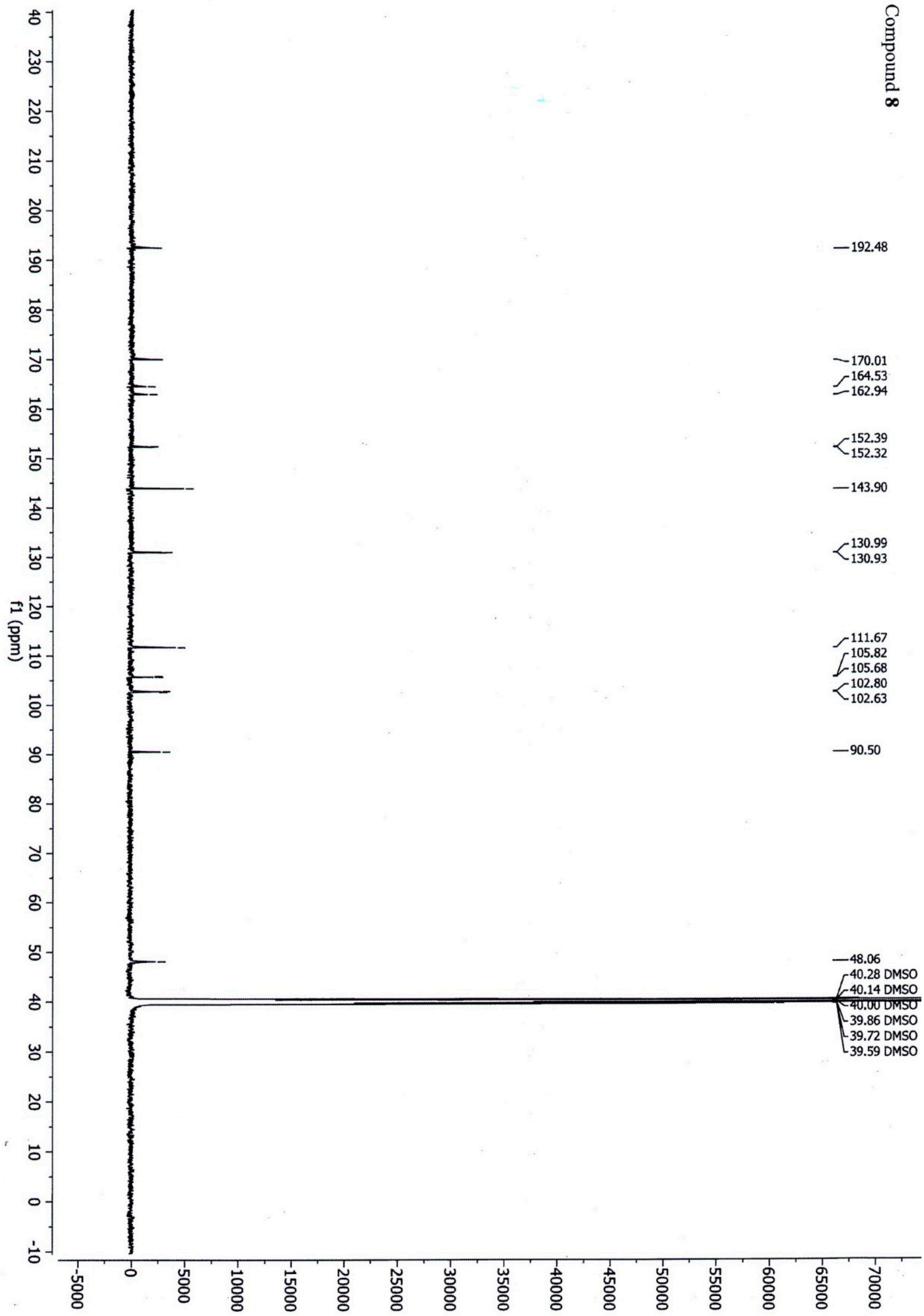

Figure S30. ^{13}C NMR spectrum of compound **8**.

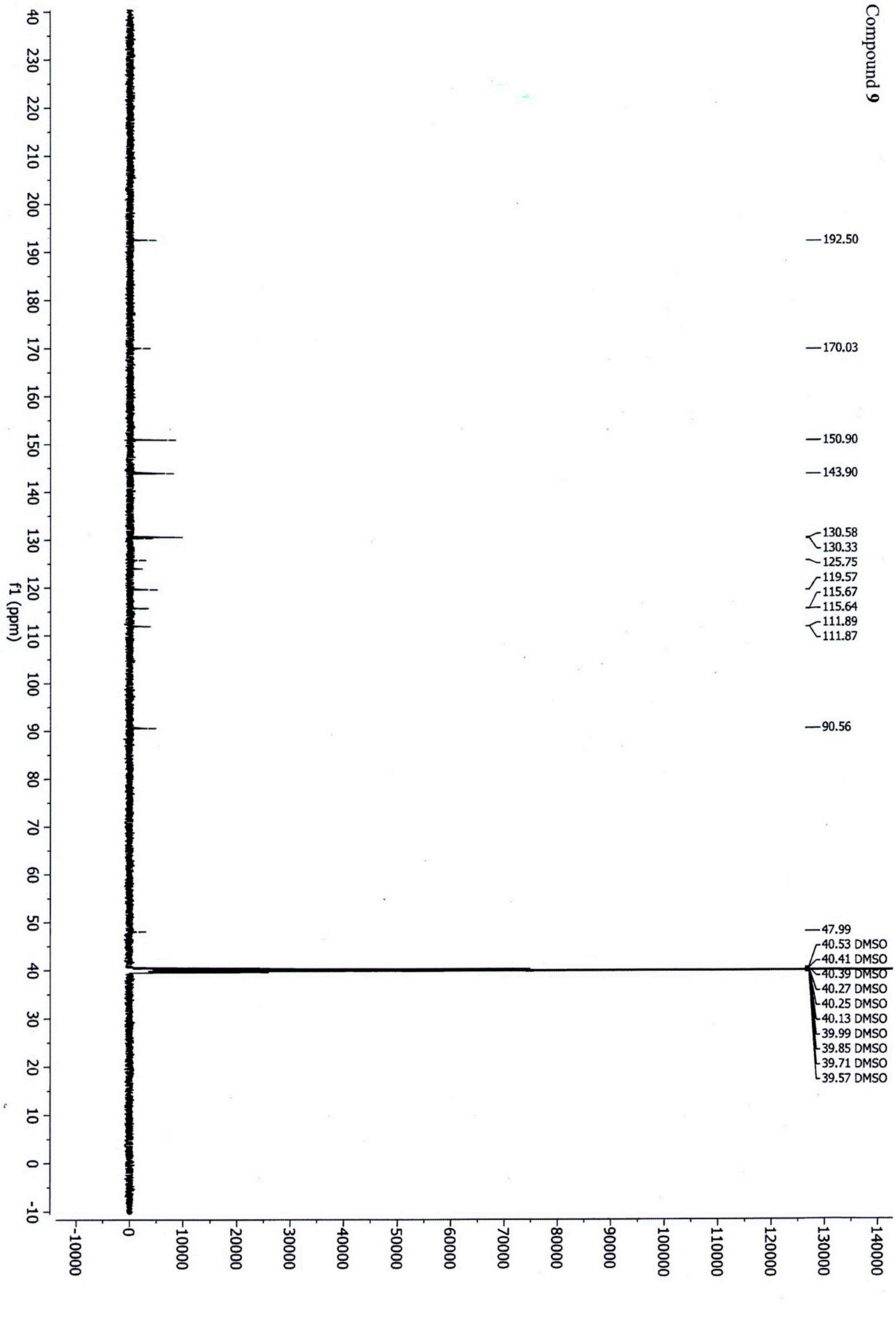

Figure S31. ^{13}C NMR spectrum of compound **9**.

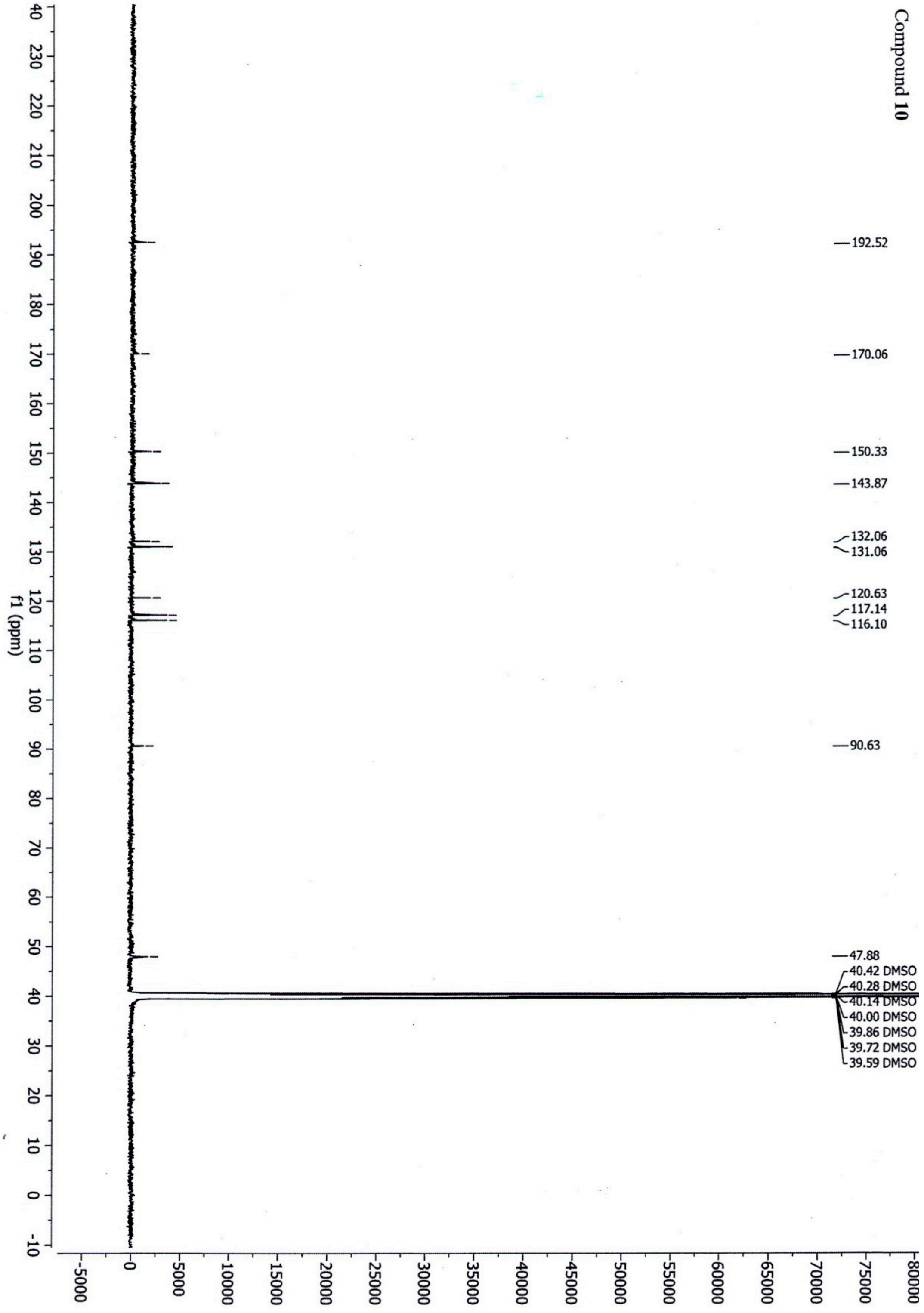

Figure S32. ^{13}C NMR spectrum of compound **10**.

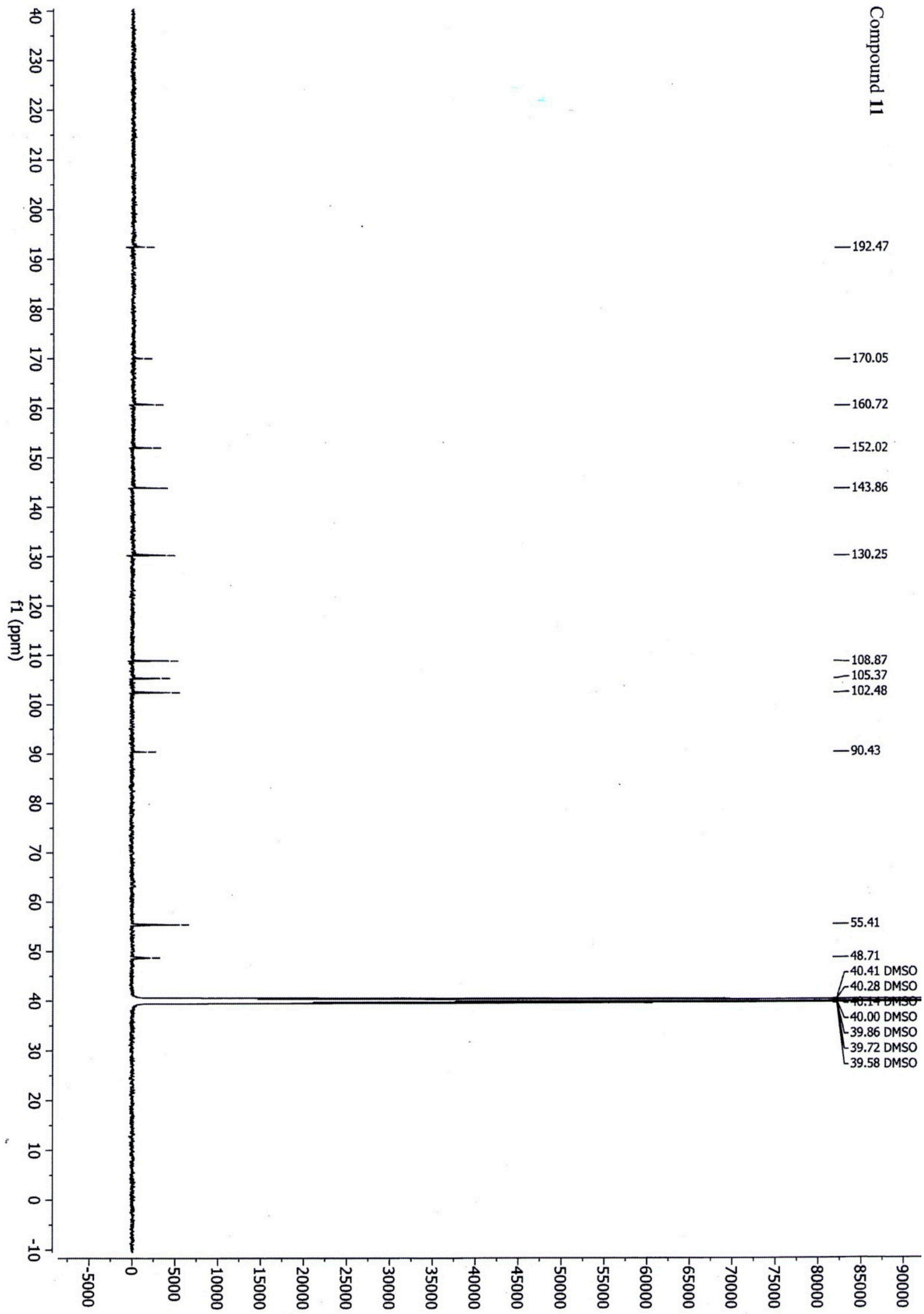

Figure S33. ^{13}C NMR spectrum of compound **11**.

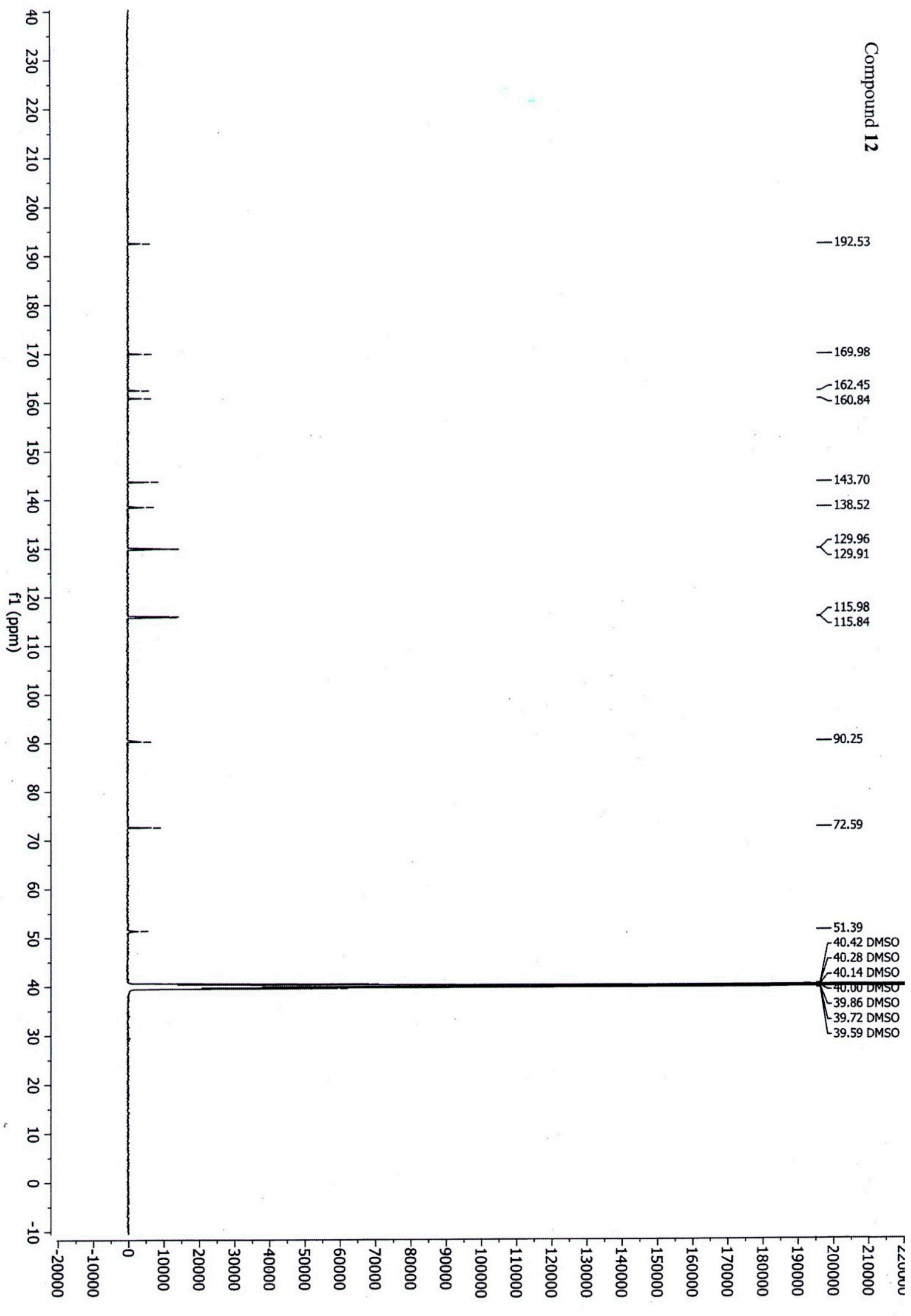

Figure S34. ^{13}C NMR spectrum of compound **12**.

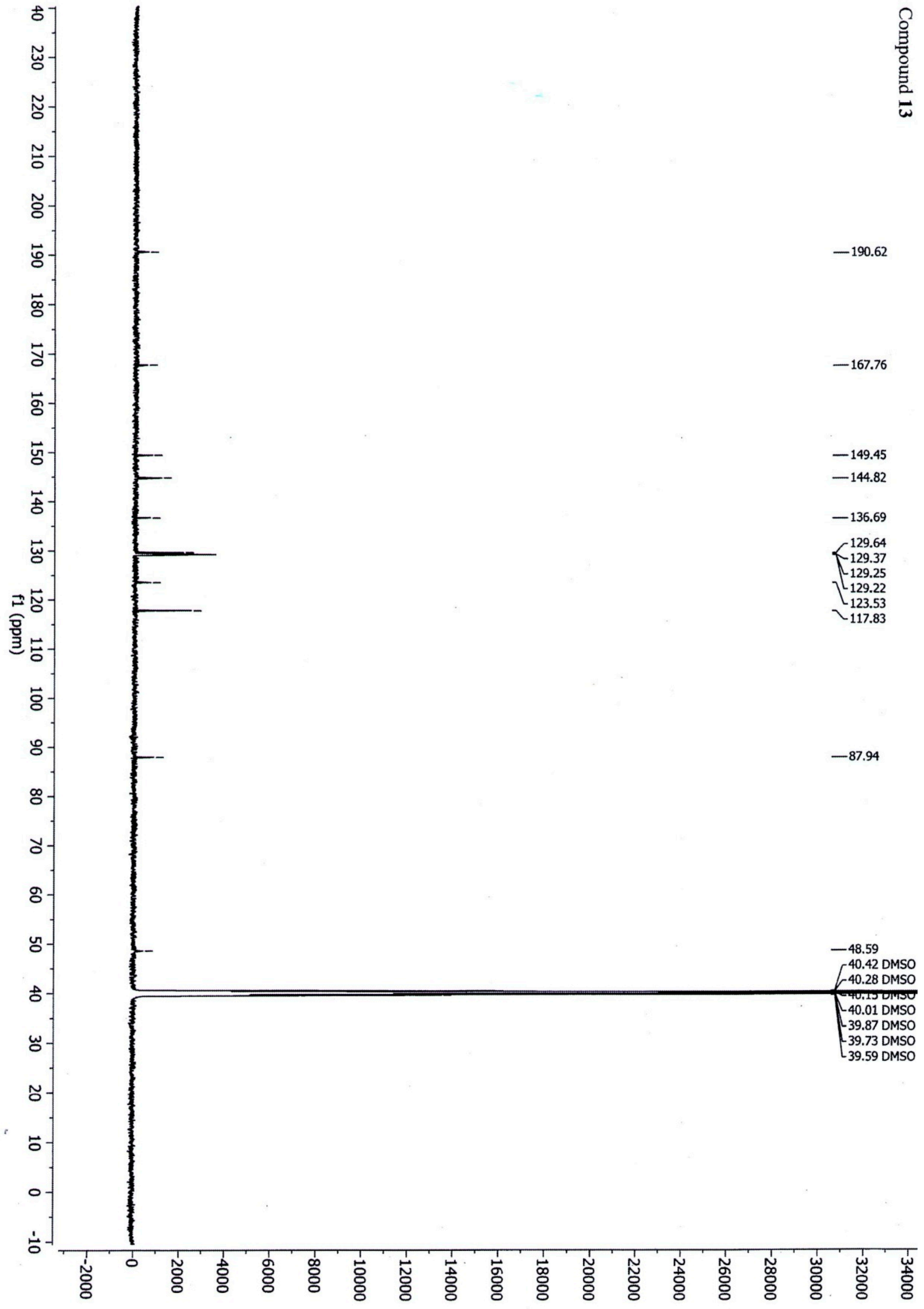

Figure S35. ^{13}C NMR spectrum of compound **13**.

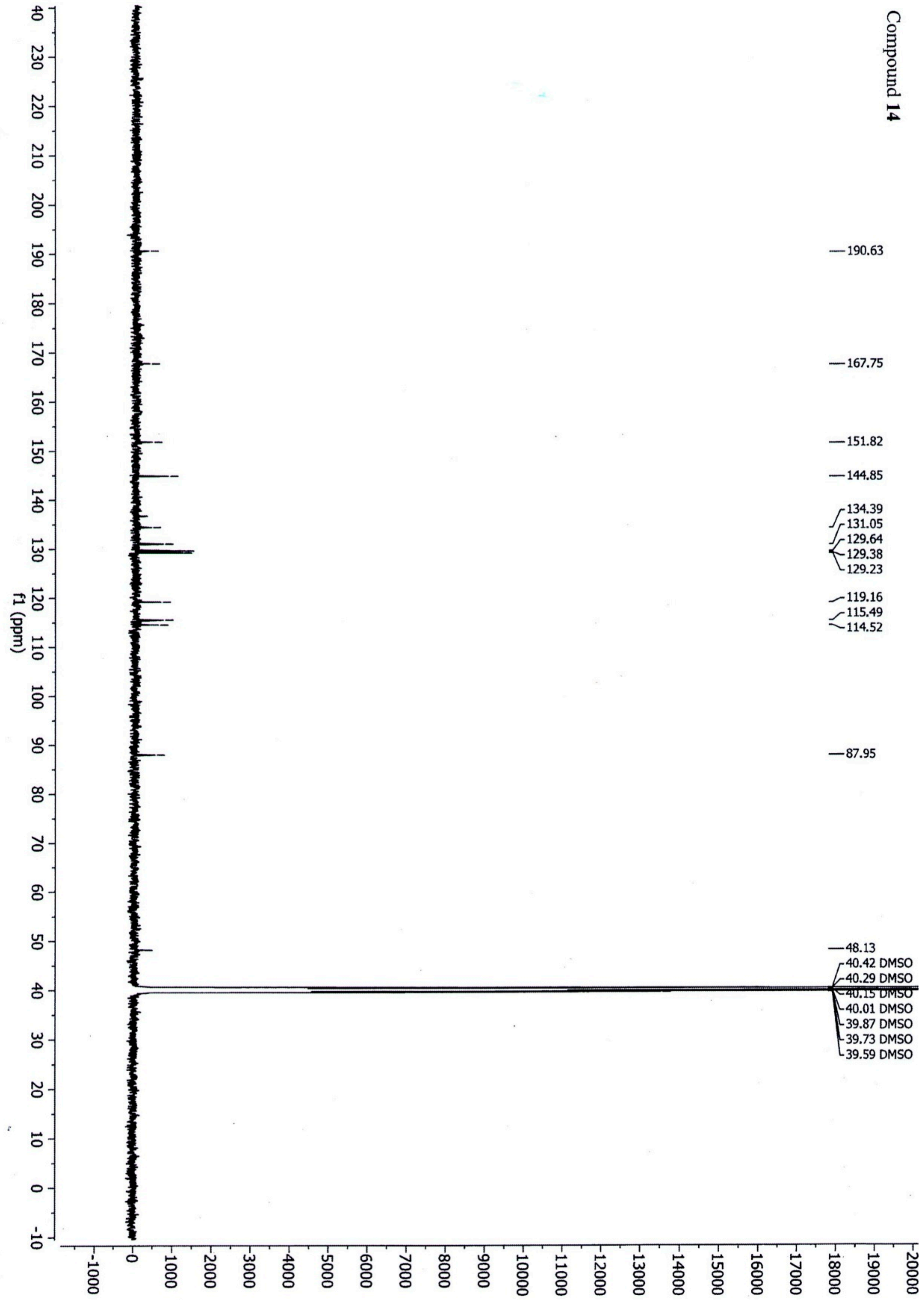

Figure S36. ^{13}C NMR spectrum of compound **14**.

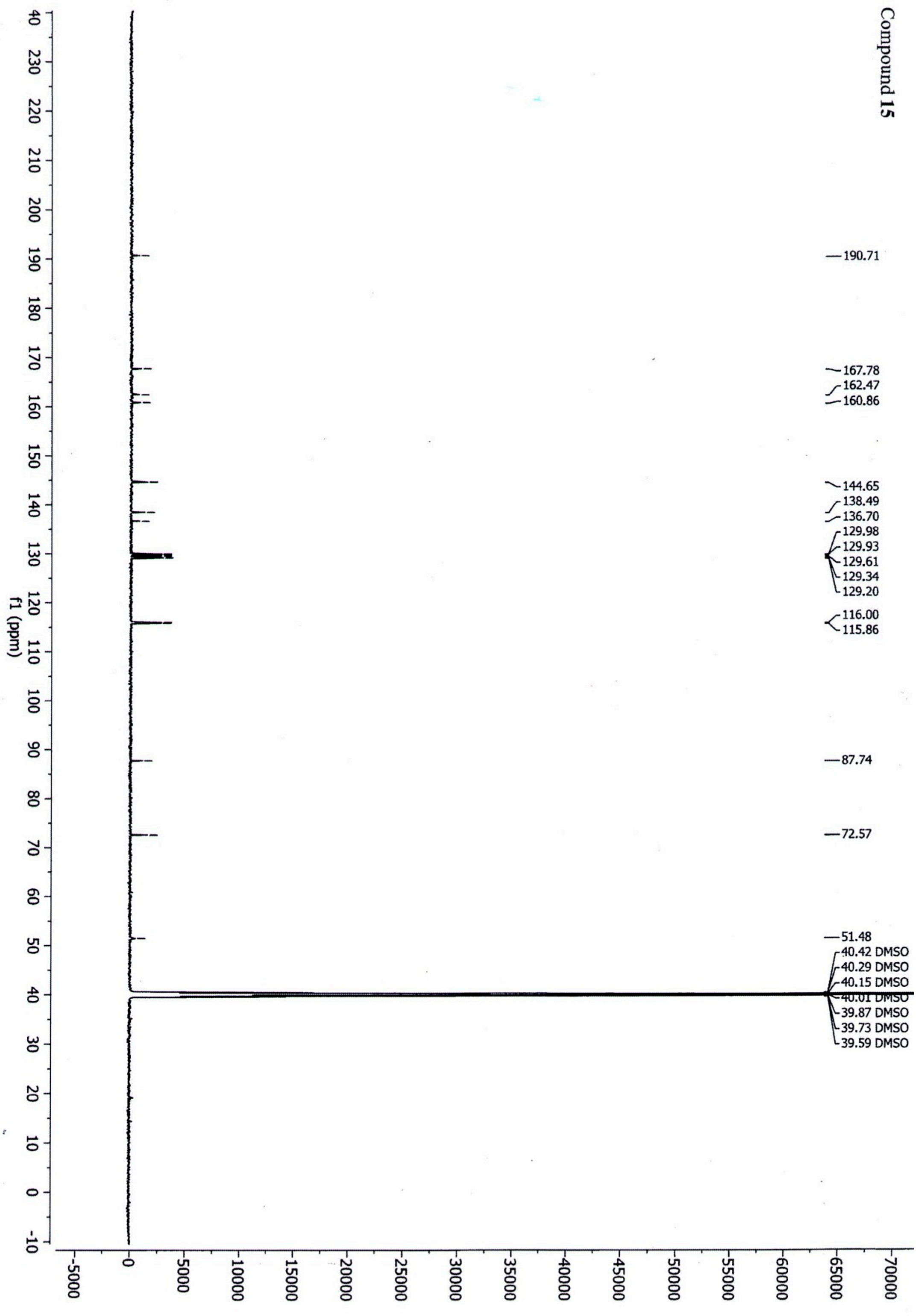

Figure S37. ^{13}C NMR spectrum of compound **15**.

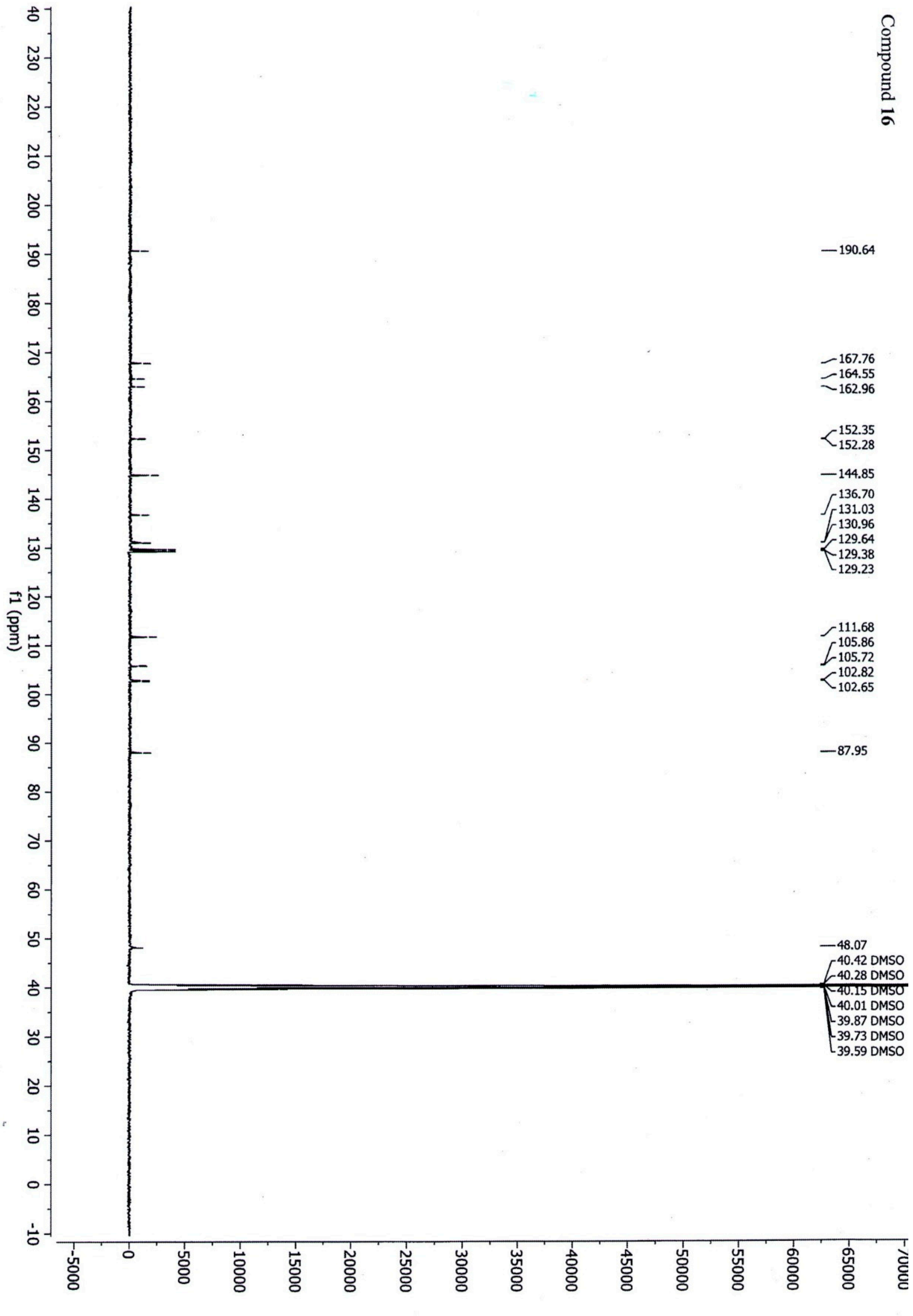

Figure S38. ^{13}C NMR spectrum of compound **16**.

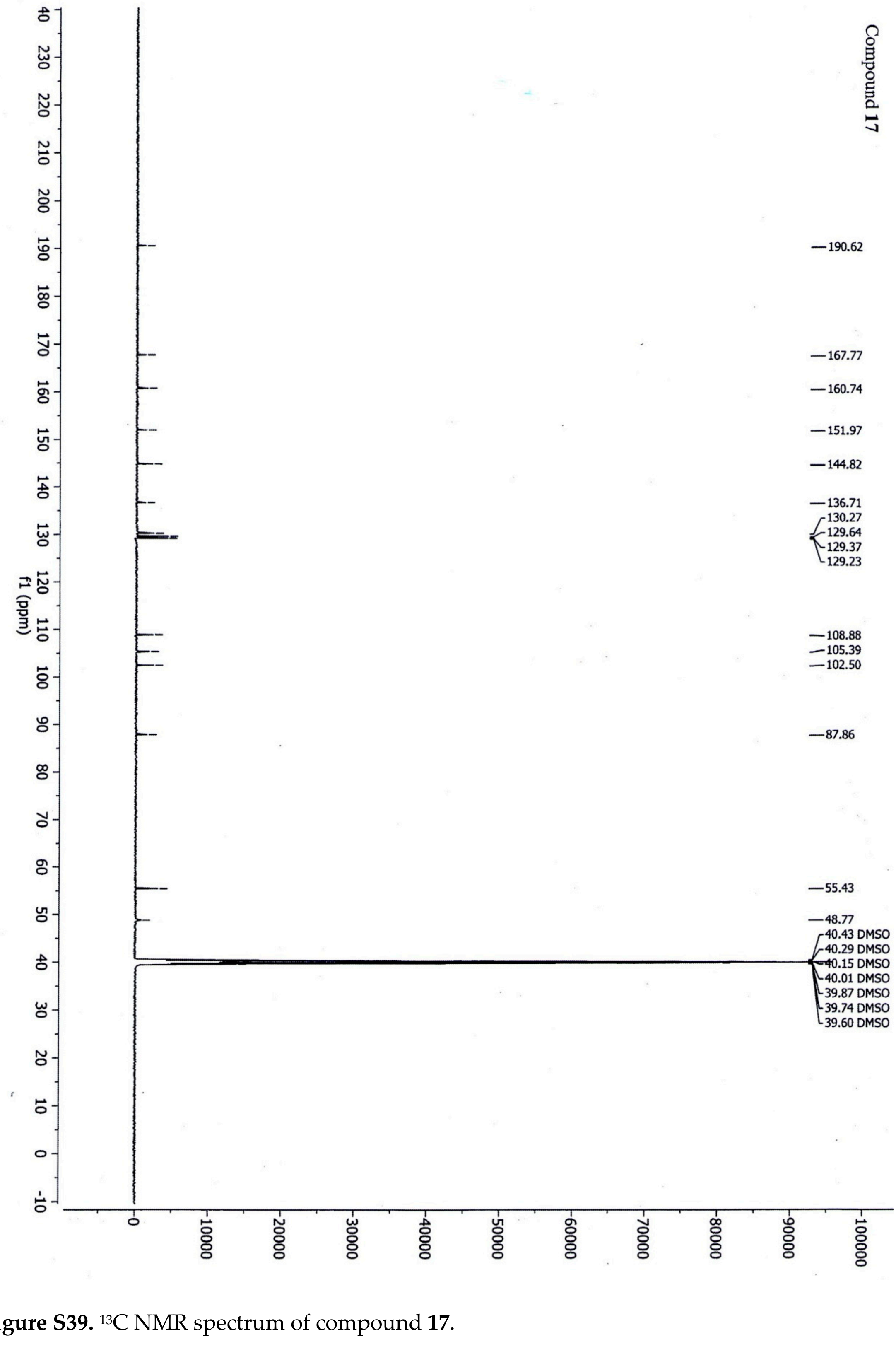

Figure S39. ^{13}C NMR spectrum of compound **17**.

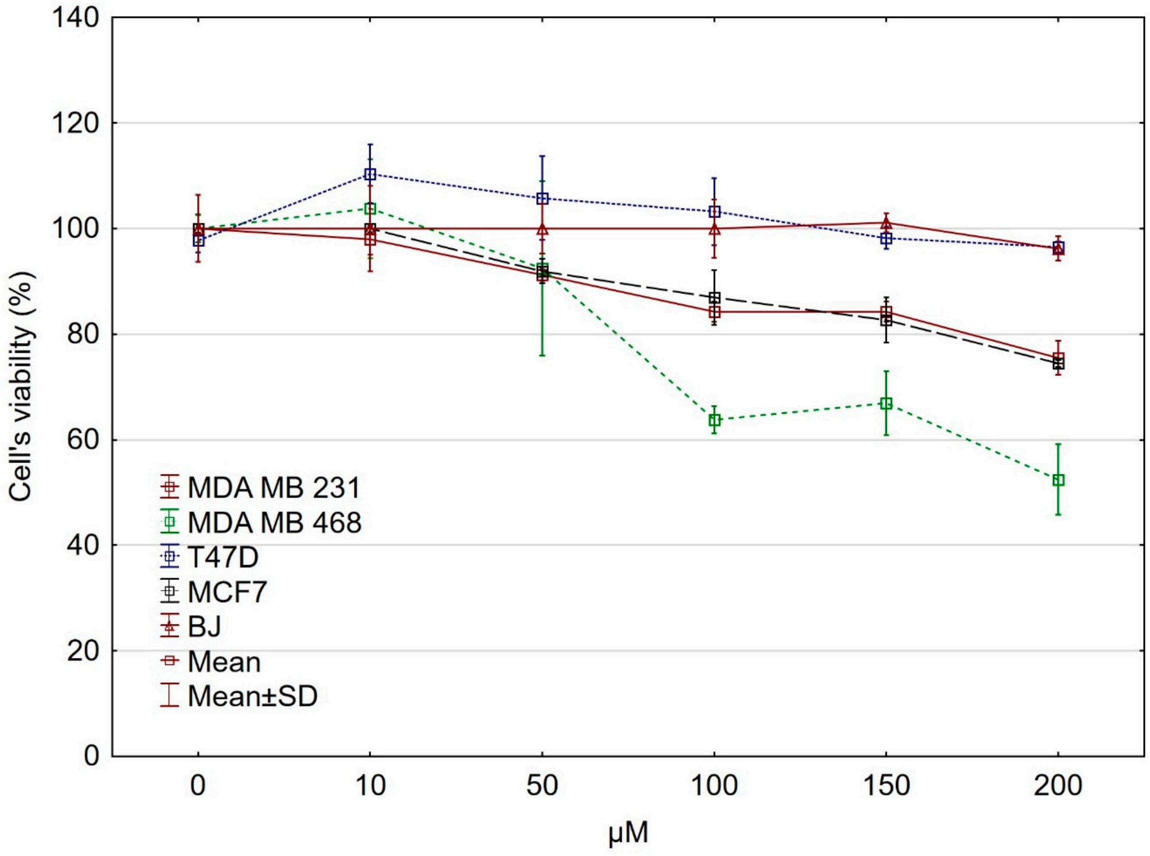

Figure S40. Toxicity curves of compound **5** based on MTT test results.

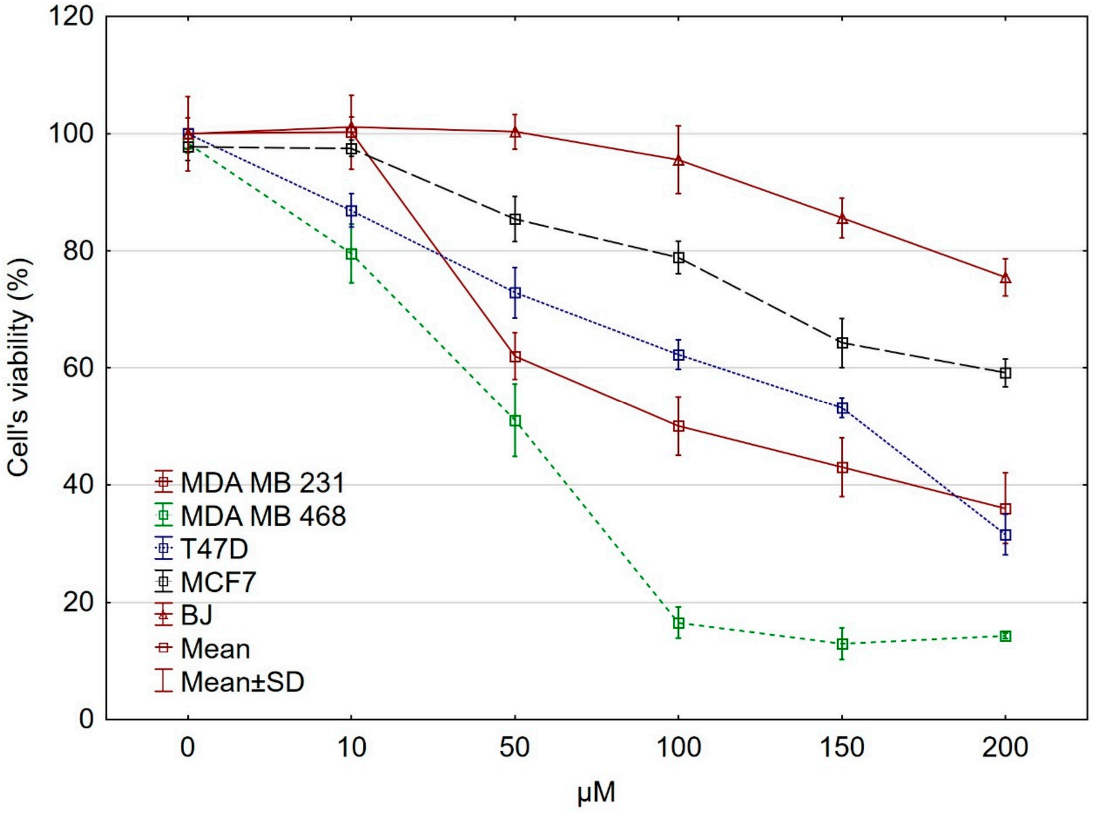

Figure S41. Toxicity curves of compound **6** based on MTT test results.

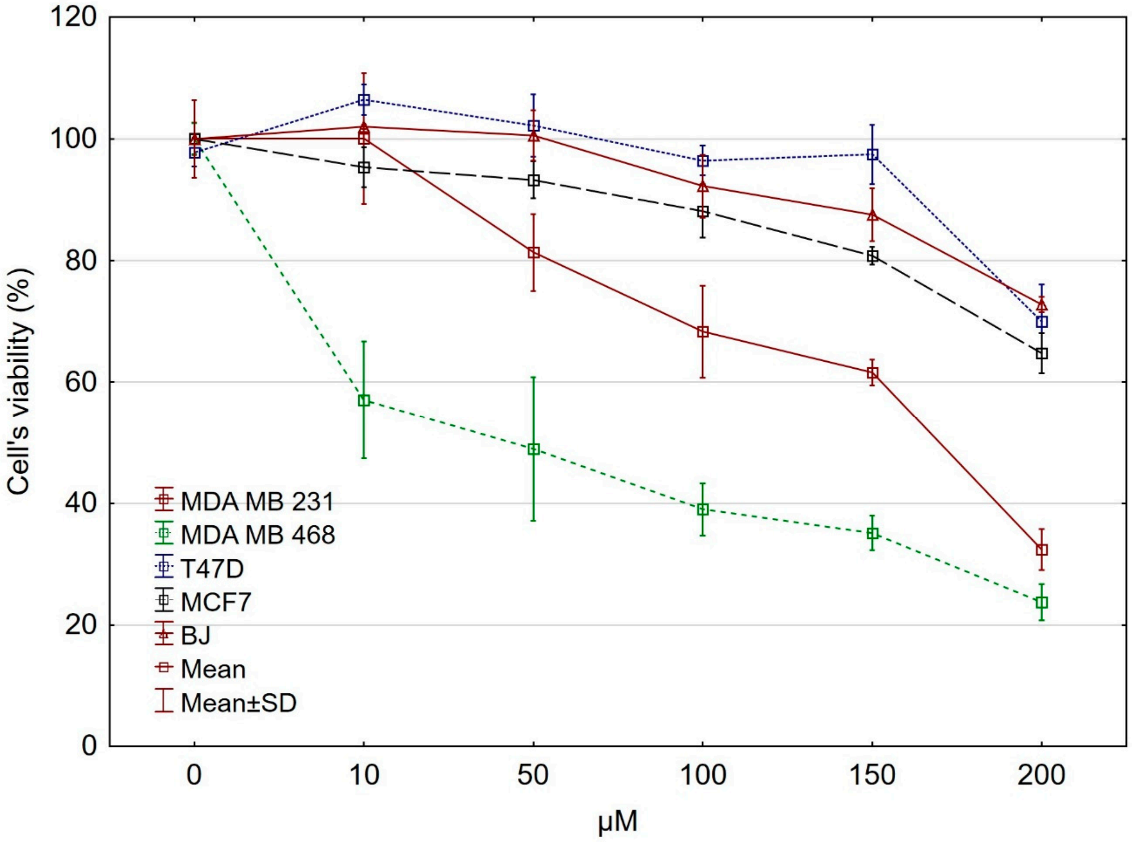

Figure S42. Toxicity curves of compound **7** based on MTT test results.

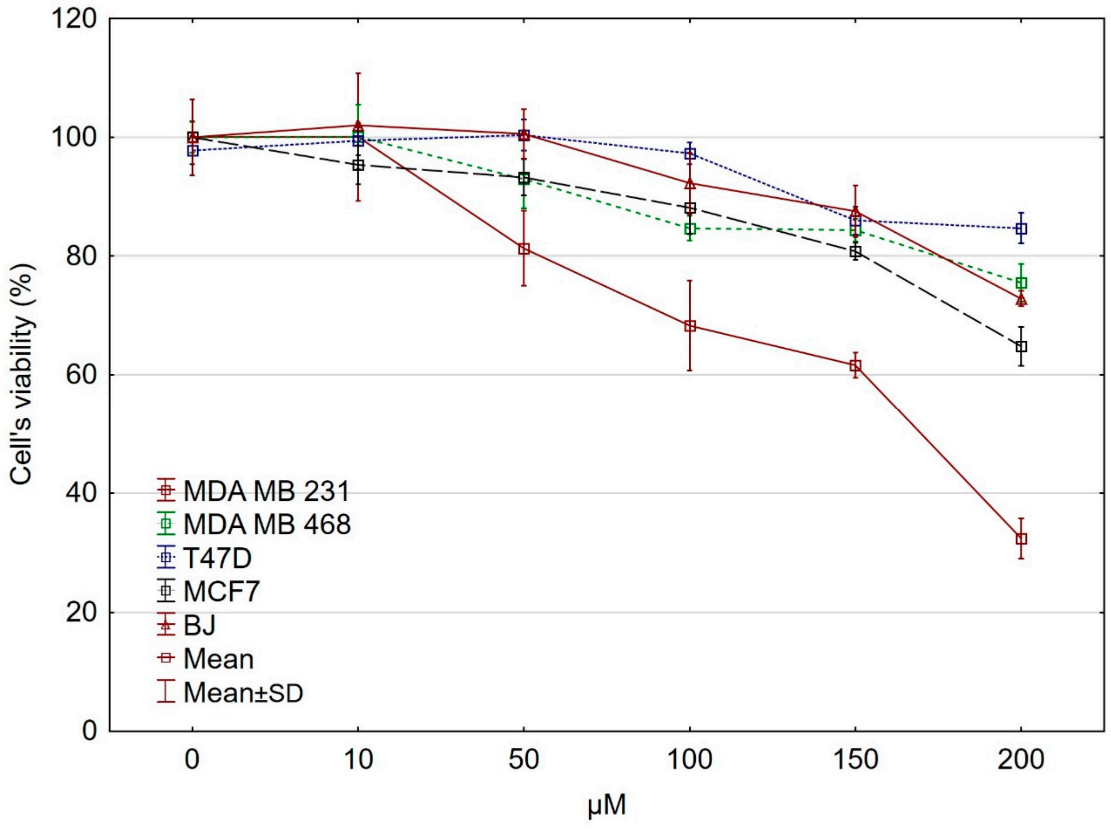

Figure S43. Toxicity curves of compound **8** based on MTT test results.

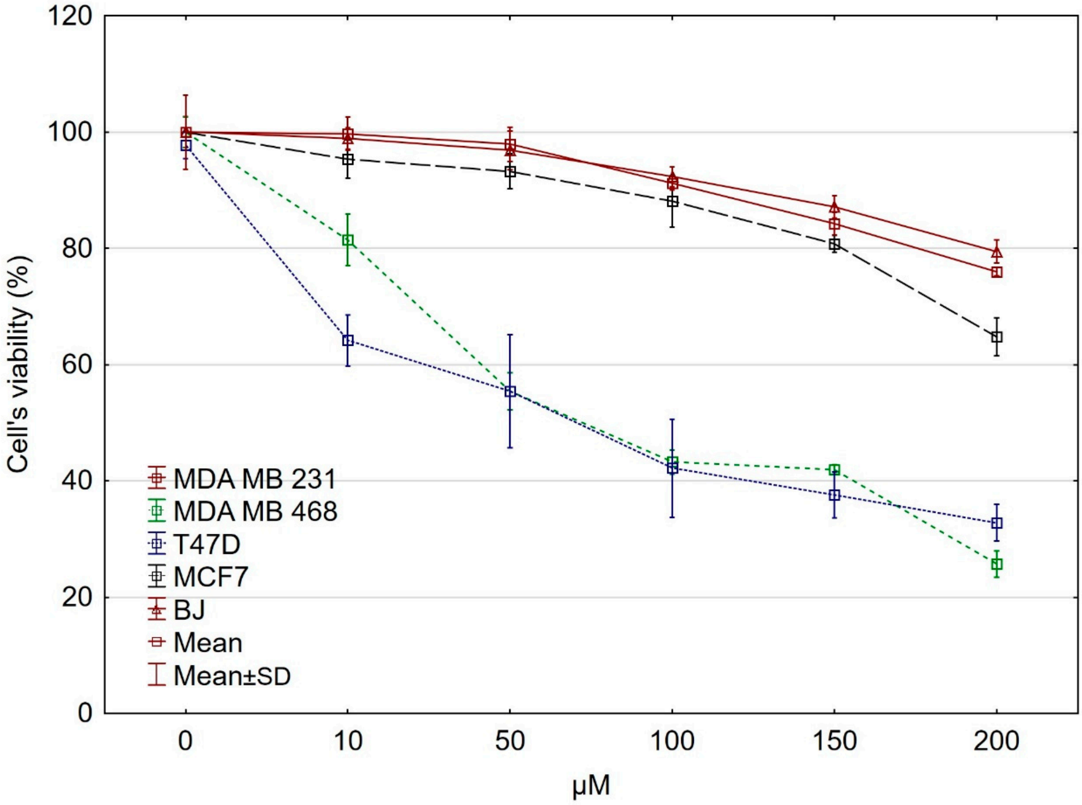

Figure S44. Toxicity curves of compound **9** based on MTT test results.

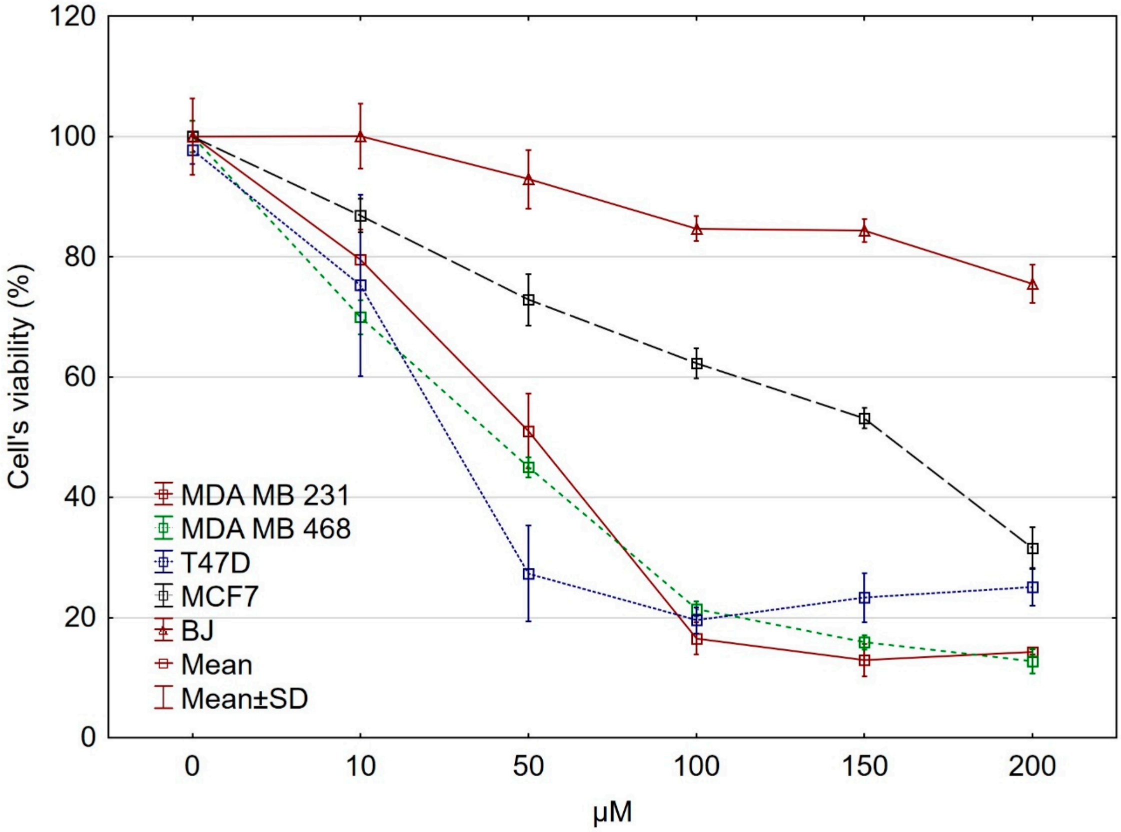

Figure S45. Toxicity curves of compound **10** based on MTT test results.

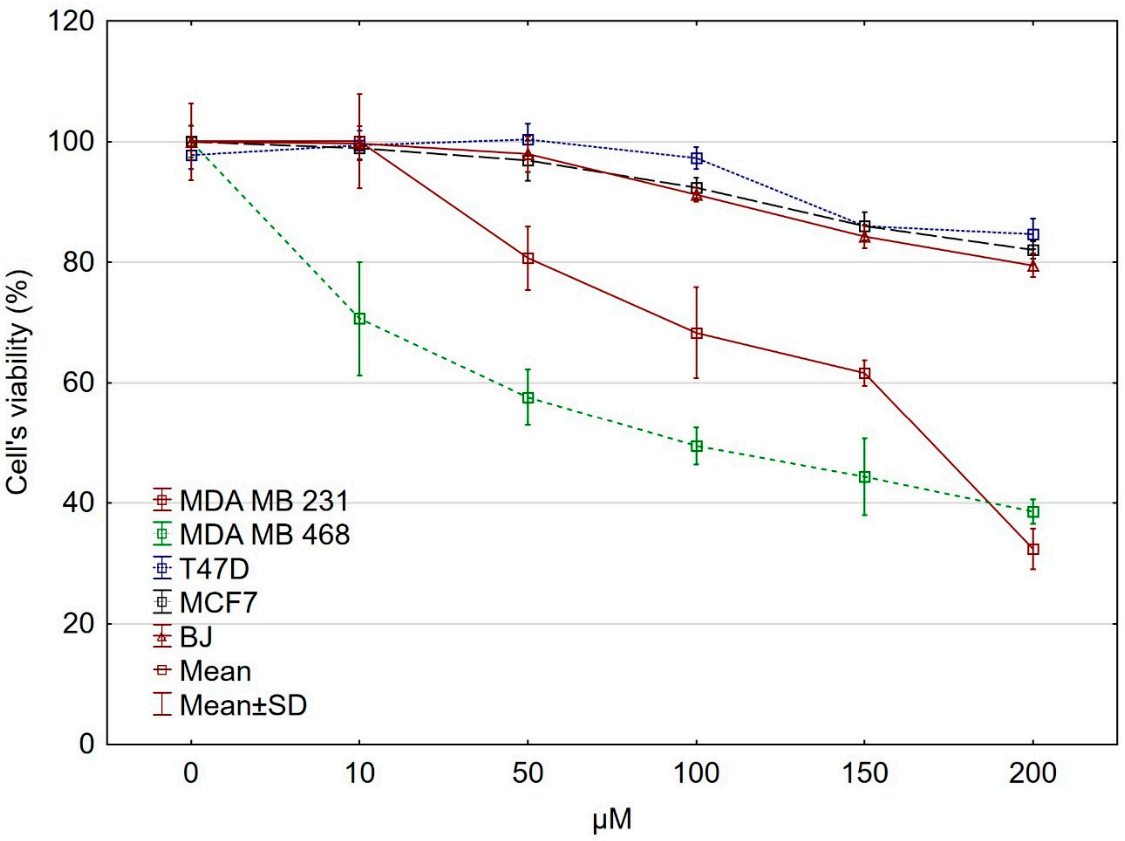

Figure S46. Toxicity curves of compound **11** based on MTT test results.

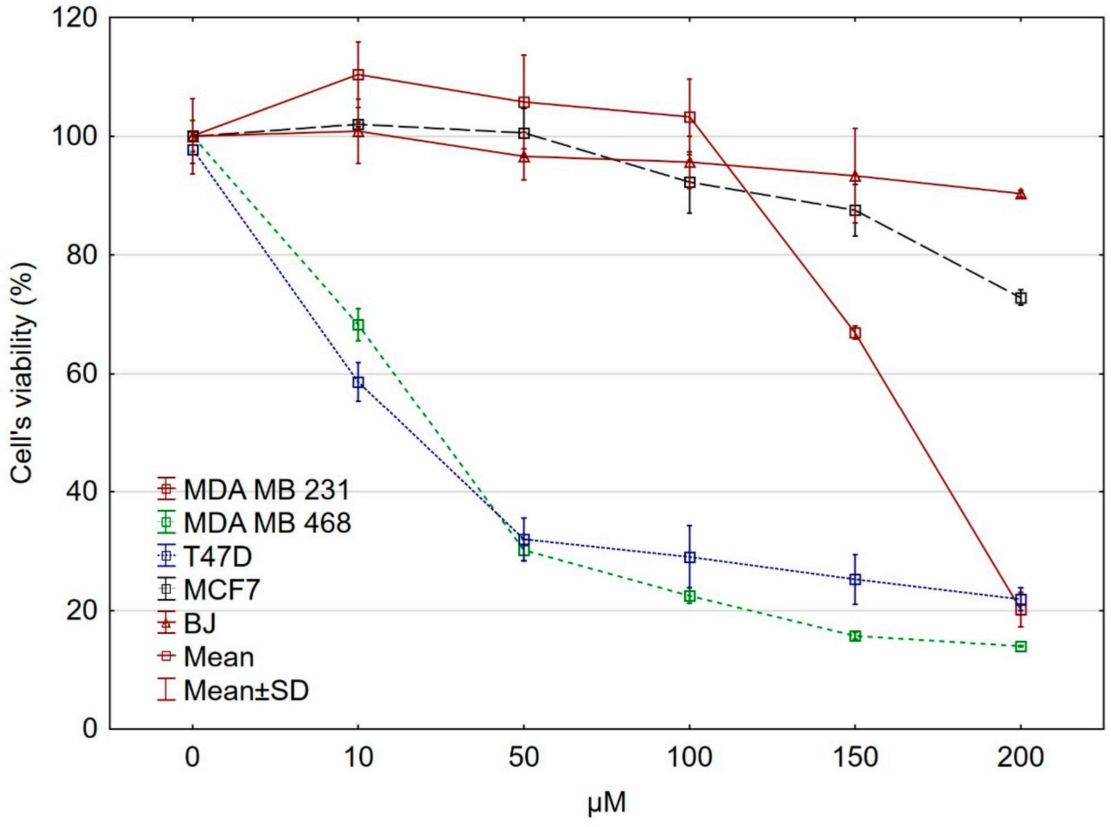

Figure S47. Toxicity curves of compound **12** based on MTT test results.

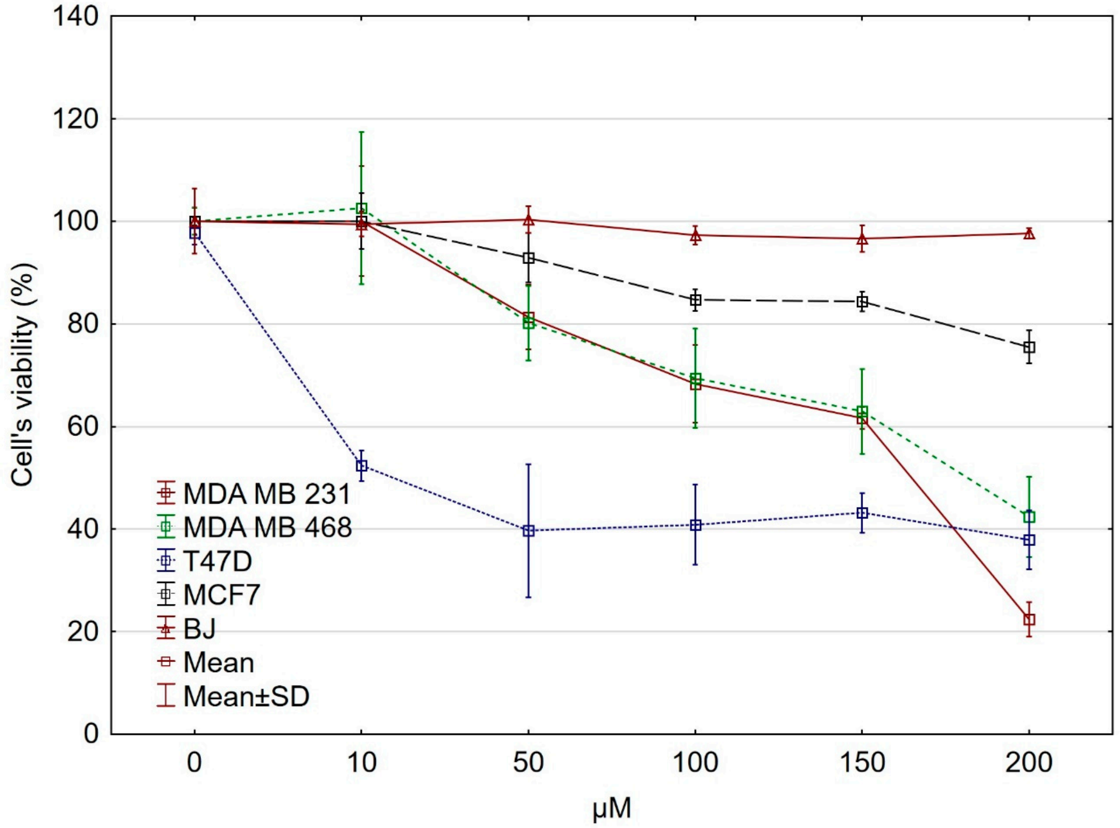

Figure S48. Toxicity curves of compound **13** based on MTT test results.

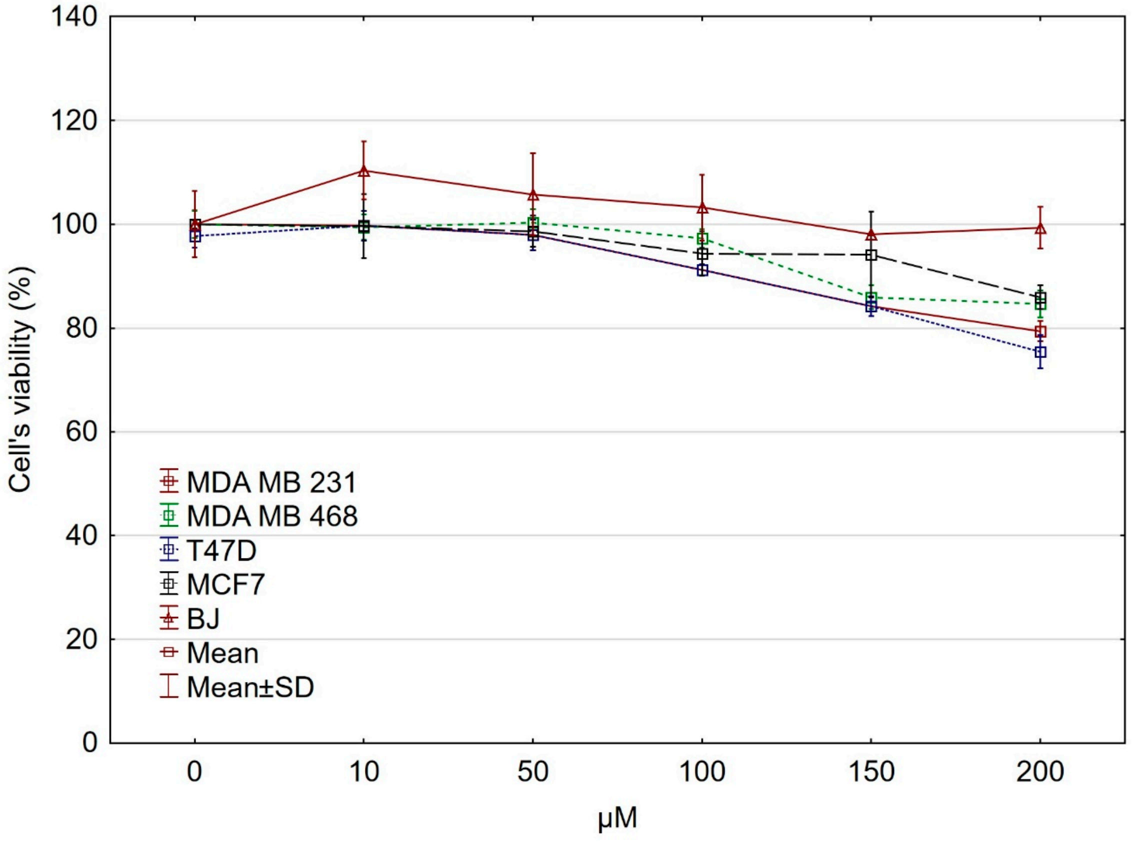

Figure S49. Toxicity curves of compound **14** based on MTT test results.

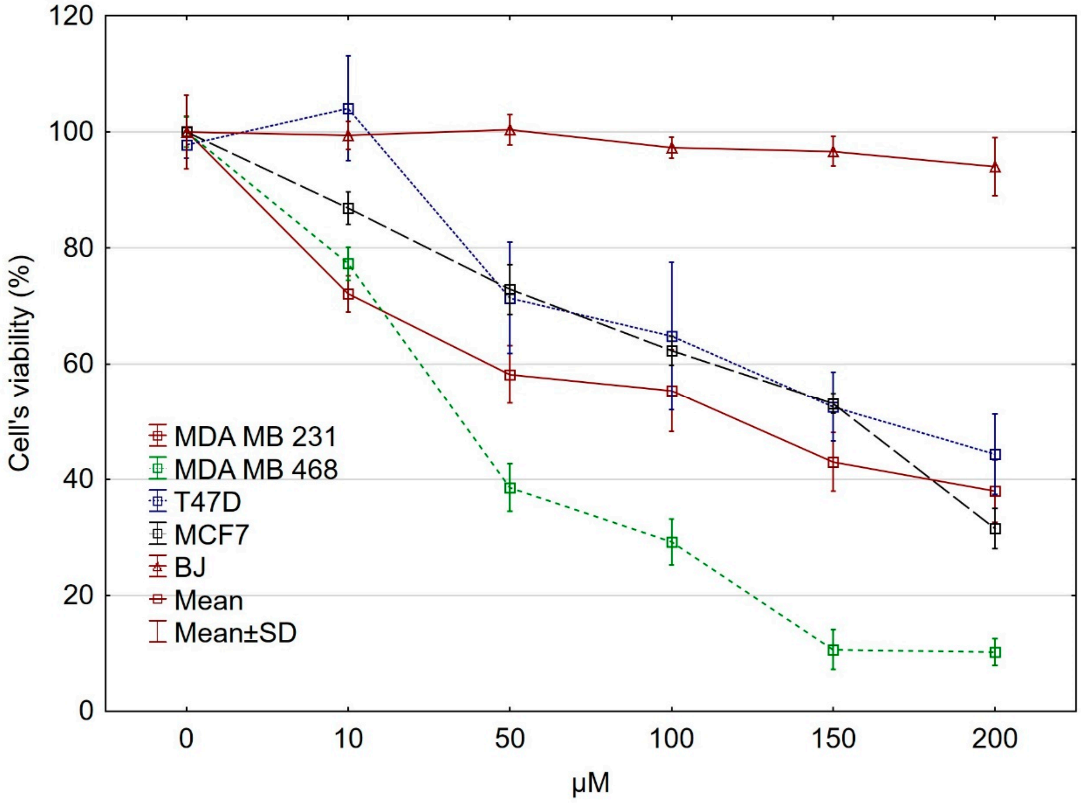

Figure S50. Toxicity curves of compound **15** based on MTT test results.

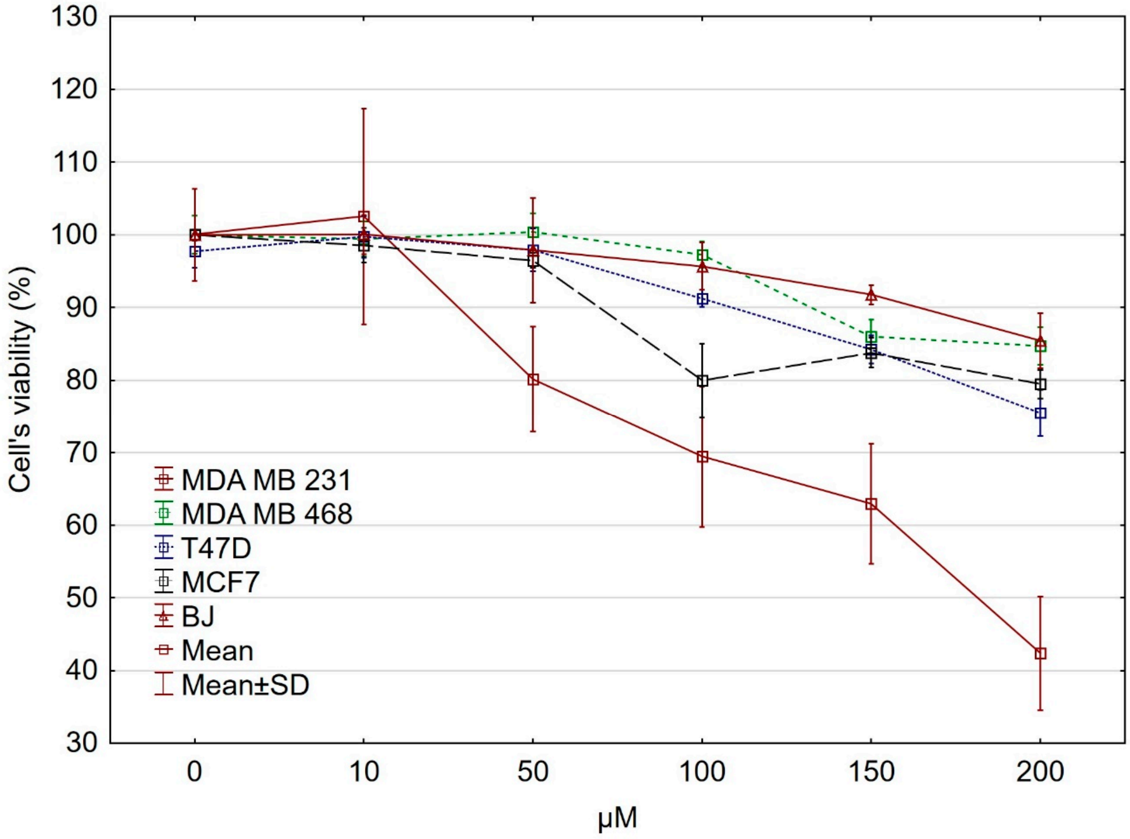

Figure S51. Toxicity curves of compound **16** based on MTT test results.

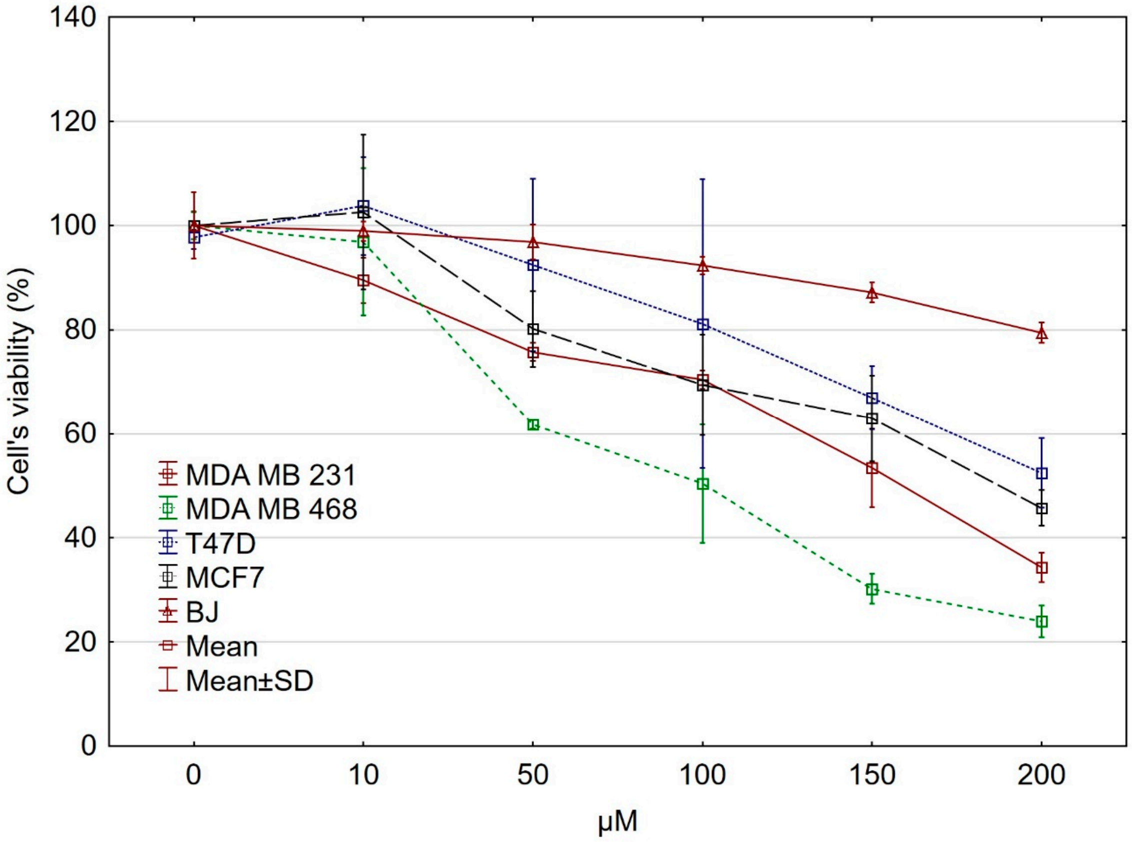

Figure S52. Toxicity curves of compound **17** based on MTT test results.
